# Emissive Liquid Crystalline Boron *C*,*N*‐Chelates: Synthesis, Self‐assembly, and Photophysical Properties

**DOI:** 10.1002/chem.202503562

**Published:** 2026-02-04

**Authors:** Franziska Müller, Falk Feucht, Alexander Beck, Ramona Seher, Anna Zens, Johannes Kästner, Yann Molard, Sabine Laschat

**Affiliations:** ^1^ Institut für Organische Chemie Universität Stuttgart Stuttgart Germany; ^2^ Institut für Theoretische Chemie Universität Stuttgart Stuttgart Germany; ^3^ Univ Rennes, CNRS, ISCR─UMR 6226, ScanMAT─UAR 2025 IETR─UMR6164 Rennes France

**Keywords:** borylation, liquid crystals, luminescence, nematic phase, phenylpyridine, smectic A phase, suzuki‐coupling, tetrahedral boron

## Abstract

A library of novel phenylpyridine‐based boron *C*,*N*‐chelates bearing a mesogenic unit was synthesized and investigated to clarify how the chain type, length, and number, as well as the boron substitution (BH_2_ vs. BMe_2_) influence the mesomorphic and photophysical behavior. All BH_2_ derivatives exhibited SmA or N phases even with short alkyl chains, whereas BMe_2_ analogues remained nonmesomorphic unless a semi‐perfluorinated chain was introduced, demonstrating the strong influence of boron substitution on mesophase formation. All compounds displayed intense blue emission in solution, with spectral properties primarily determined by the boron *C*,*N* core and only minor shifts induced by variation in the mesogenic unit in agreement with complementary DFT calculations. Quantum yields reached up to 100% in solution. These results demonstrate that mesomorphic behavior can be introduced and tuned while preserving the excellent photophysical performance of the boron‐*C*,*N*‐chelate system.

## Graphical abstract

Novel emissive liquid crystalline boron *C,N*‐chelates were prepared by integrating mesogenic units into the boron *C*,*N*‐core. Depending on the substituent at the boron, stable SmA or N mesophases were achieved while maintaining intense blue emission with excellent quantum yields (61%–100%).

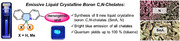

## 1 Introduction

**SCHEME 1:**
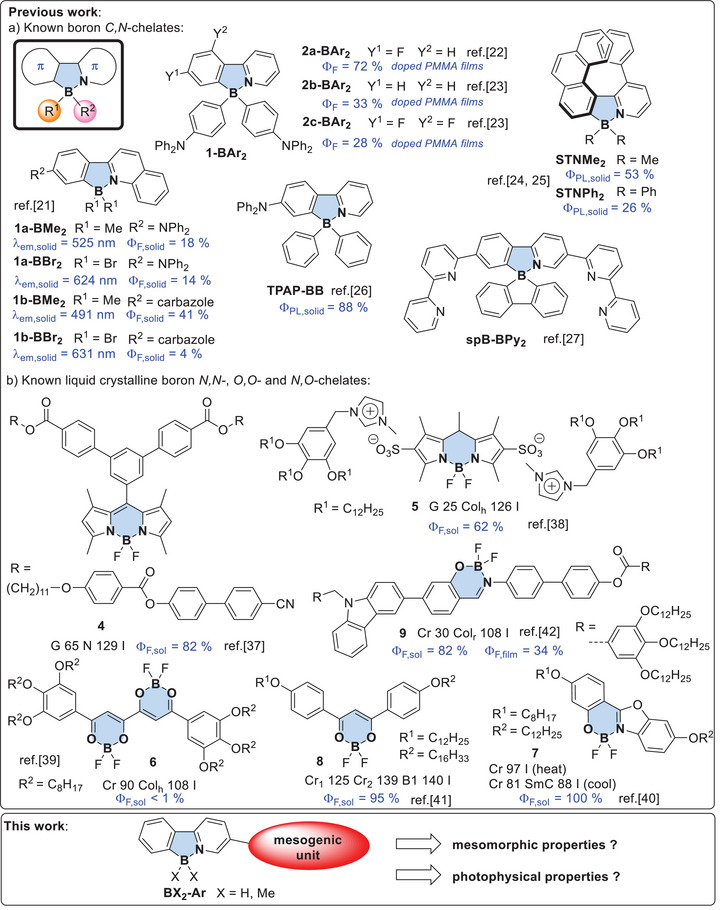
(a) Previously reported boron *C,N*‐chelates, (b) previously reported boron *N,N*‐, *O,O*‐, and *N,O*‐chelates, (c) our targeted structure for the development of novel liquid crystalline boron *C,N*‐chelates.

Tetra‐coordinated boron *O*,*O*‐, *N*,*N*‐, *O*,*N*‐, and *C*,*N*‐chelates have received increasing interest as promising photoactive materials for luminescence sensing, imaging, anticounterfeiting [1], photovoltaic devices [2], and organic light emitting‐diodes [3, 4]. The trigonal planar B‐fragment is isoelectronic with carbenium ions and possesses Lewis acidic character [5]. By proper coordination of the vacant p_Z_ orbital with a Lewis base the photoluminescence properties of the resulting tetra‐coordinated boron chelates can be tailored. Chelate ligands as well as ancillary ligands R^1^,R^2^ not only exert electronic and steric effects on the central B unit but also increase the stability as compared to the corresponding tricoordinated B compounds, in particular when π‐conjugated *C,N*‐chelate ligands were employed [4]. The photophysical properties and broad applications have stimulated much synthetic effort resulting in a huge and diverse library of boron *C,N*‐chelates [6, 7, 8, 9]. The phenylpyridine boron unit is an archaetypal example of boron *C,N*‐chelates, which is obtained by aromatic borylation [10, 11, 12] or borenium cation mediated boro‐desilylation [13]. Mechanistic and theoretical studies provided insight into the photoreactivity [14, 15, 16, 17] and formation of chiral derivatives [16], for example via S_N_2‐type chirality transfer from boron *O,N*‐chelates [18, 19]. The Lewis acidity and fluorescence properties would be tailored by employing weakly coordinating anions [20]. Boron *C,N*‐chelates containing the phenylpyridine unit possess several interesting features such as color tunability by ligand alteration reported for **1a** and **1b** (Scheme 1a) [21], or thermally activated delayed fluorescence (TADF) observed for compounds **2a**, **2b** and **2c**, respectively [22, 23] The impact of truncation on the optoelectronic properties of azaborole helicenes **STNMe_2_** and **STNPh_2_** has been studied. Compound **STNMe_2_** displayed quantum yield of Φ_PL_ = 53 % in the solid state and blue circularly polarized luminesence (CPL, ρ_PL_ ≤ 3 ^●^ 10^−3^) [24, 25]. 3‐Diphenylamino‐6‐(2‐pyridinyl)phenyldiphenylboron **TPAP‐BB** showed much higher fluorescence quantum yield in the solid state (Φ_PL_  =  88 %) than in THF solution (Φ_PL_  =  49 %) due to aggregation‐induced emission (AIE), which was applied for apoptosis monitoring and *in vivo* imaging [26]. Bis(bipyridine)‐substituted *spiro*‐boron‐*C,N*‐chelate **spB‐Bpy_2_** was used as a host in inverted OLEDs (iOLEDs), where electron injection was facilitated by hydrogen bonding between the host **spB‐Bpy_2_** and bases [27].

It has been reported by several groups, including our own, that liquid crystalline self‐assembly of emitter molecules can have beneficial effects on the photophysical properties [28]. The long‐range orientational order and fluidity of the mesophase facilitates self‐healing of defects, enables surface alignment, and improves electronic interaction between emitter molecules due to the close proximity in the mesophase [28, 29, 30, 31, 32, 33, 34]. Thus, several liquid crystalline boron *N,N*‐, *O,O*‐, and *N,O*‐chelates have been reported, showing a variety of different mesophases [35, 36]. Some selected examples are shown in Scheme 1b, for example BODIPY derivative **4** forming nematic (N) phase [37], or ionic liquid crystalline BODIPY **5** displaying hexagonal columnar (Col_h_) mesophases [38]. β‐Tetraketonates **6** self‐assembled into columnar mesophases [39]. On the other hand benzoxazoles **7** formed monotropic smectic C (SmC) phases and displayed a violet emission with a quantum yield of 100% [40]. For β‐diketonato boron complexes **8** banana (B_1_) phases and high quantum yields were reported [41]. More recently, carbazole‐capped Schiff base BF_2_ complexes **9** were disclosed, showing luminescent square columnar (Col_squ_) mesophases with high quantum yields, which were employed as turn‐on sensors for Al^3+^ and white light emitting diodes [42]. However, surprisingly no liquid crystalline boron *C,N*‐chelates were described in the literature. Therefore, we surmised that grafting of a single side chain to tetra‐coordinated boron *C,N*‐chelates might lead to calamitic emissive liquid crystals. Indeed, as detailed below the resulting monoalkoxy or monoalkyl‐substituted derivatives **BX_2_‐Ar** displayed nematic and/or smectic A mesophases and promising photophysical properties.

## 2 Results and Discussion

### 2.1 Synthesis of Boron *C*,*N*‐chelates

**SCHEME 2:**
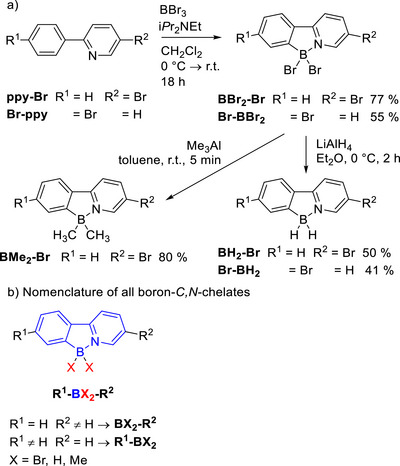
(a) Synthesis of the boron‐*C,N*‐chelates BBr_2_‐Br and Br‐BBr_2_ via electrophilic aromatic borylation and further modification with metal reagents to BMe_2_‐Br, BH_2_‐Br, and Br‐BH_2_, (b) nomenclature of all boron‐*C*,*N*‐chelates.

The synthesis of the boron *C*,*N*‐chelates **BX_2_‐Ar** and **Ar‐BX_2_** started with the introduction of the tetrahedral boron core (Scheme 2). Phenylpyridine‐based ligands **ppy‐Br** and **Br‐ppy** were treated with BBr_3_ and Hünig's base in CH_2_Cl_2_ at room temperature for 18 h, affording the chelates **BBr_2_‐Br** and **Br‐BBr_2_** in 55 % and 77 % via electrophilic aromatic borylation following the procedure from Ishida et al. [11]. The tetrahedral boron *C*,*N‐*coordination was confirmed via ^11^B‐NMR (δ  =  ‐1.1–‐1.8 ppm, Supporting Information Figures S70,S72). For further modification at the central boron atom, the chelates were subjected to reactions with metal reagents. Thus, treatment of **BBr_2_‐Br** and **Br‐BBr_2_** with LiAlH_4_ suspended in Et_2_O at 0°C for 2 h afforded the desired products **BH_2_‐Br** and **Br‐BH_2_** in 41–50 % yield. Because of the low solubility of **BBr_2_‐Br** and **Br‐BBr_2_** in Et_2_O, the reaction with LiAlH_4_ was attempted in THF, but in both cases only decomposition of the starting materials was observed. The reaction of **BBr_2_‐Br** with Me_3_Al in toluene at room temperature for 5 min provided the methylated derivative **BMe_2_‐Br** in 80% yield, which required no further purification after aqueous work‐up with saturated Rochelle salt solution.

**SCHEME 3:**
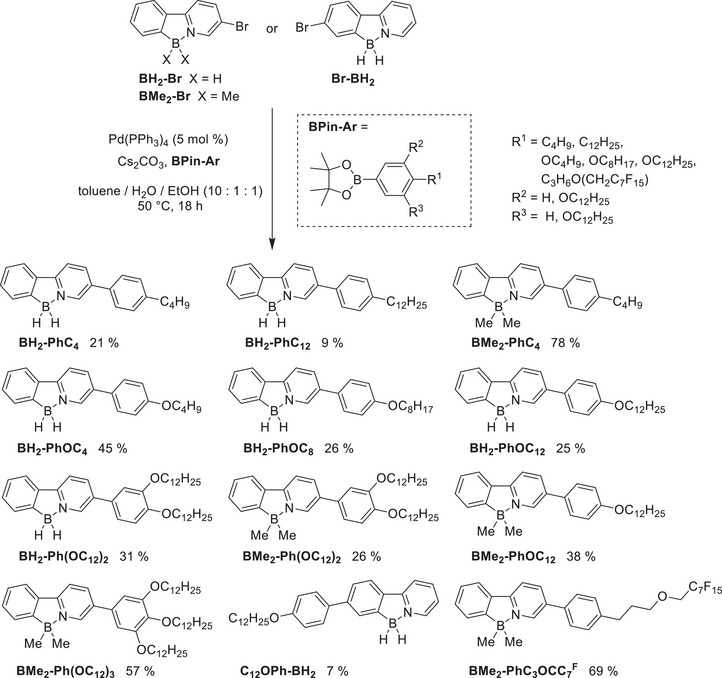
Synthesis of mesogenic boron *C,N*‐chelates BH_2_‐Ar, BMe_2_‐Ar, and C_12_OPh‐BH_2_ with different substitution pattern.

The combination of mesomorphic properties with the characteristic emission behavior of tetrahedral boron compounds was targeted through the introduction of flexible side chains and extension of the aromatic system. To achieve this with a minimum of additional steps, the chelate complexes **BH_2_‐Br, Br‐BH_2_**, and **BMe_2_‐Br** were coupled with various substituted pinacol‐borolanes **BPin‐Ar** via Suzuki‐coupling (Scheme 3). As mesogenic side groups, alkyl and alkoxy chains of different lengths as well as a semi‐perfluorinated chain were employed. To establish suitable conditions for the Suzuki coupling, the reaction of **BH_2_‐Br** and **BPin‐OC_12_** was used as a model system (Table 1).

**TABLE 1:** Optimization of Suzuki coupling of BH_2_‐Br with BPin‐OC_12_ to BH_2_‐PhOC_12_ under various conditions.

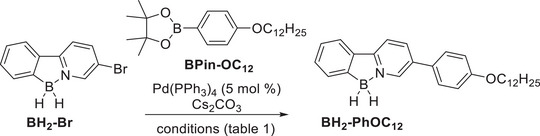				
entry	solvent	*T* /°C	*t* / h	yield / %
1	toluene / H_2_O (10 : 1)	90	24‐48	traces ^a^, decomposition
2	toluene / H_2_O (10 : 1)	30	24‐48	no conversion
3	THF / H_2_O (10 : 1)	90	18	decomposition
4	dioxane	90	18	decomposition
5	DME / H_2_O (1 : 1)	85	18	24
6	toluene / H_2_O / EtOH (10 : 1 : 1)	50	18	25
7	toluene / H_2_O / EtOH (10 : 1 : 1)	75	18	7
8	DME / H_2_O (10 : 1)	70	18	decomposition

^a^ Detected via HRMS.

Initial attempts under adapted literature conditions in toluene / H_2_O (10 : 1) at 90°C for 24–48 h afforded only traces of the product (detected via HRMS) alongside decomposition of **BH_2_‐Br** (entry 1) [11]. Lowering the temperature to 30°C resulted in no conversion and recovery of the starting material **BH_2_‐Br** (entry 2). Reactions in THF / H_2_O (10 : 1) or dioxane also led to decomposition of the B‐N moiety as confirmed via ^11^B‐NMR (entries 3, 4). Changing the solvent to DME / H_2_O (1 : 1) at 85°C for 18 h afforded **BH_2_‐PhOC_12_** in 25% yield (entry 5). A similar result was obtained in toluene / H_2_O / EtOH (10 : 1 : 1) at 50°C for 18 h, giving **BH_2_‐PhOC_12_** in 24% yield (entry 6). Increasing the reaction temperature to 70°C led to only 7 % yield (entry 7). Using DME / H_2_O with another solvent ratio (10 : 1) was also not successful (entry 8).

The optimized conditions toluene / H_2_O /EtOH (10 : 1 : 1) at 50°C for 18 h afforded a series of derivatives with varying side chains (alkyl, alkoxy and perfluorinated) and substitution patterns on the pyridine moiety (Scheme 3). **BH_2_‐Br** was coupled with **BPin‐Ar** to obtain six derivatives **BH_2_‐Ar** in 21–49 % yield, while coupling of dimethyl chelate **BMe_2_‐Br** yielded five derivatives **BMe_2_‐Ar**.

Overall, reactions with **BMe_2_‐Br** afforded higher yields (26–78 %), indicating greater stability of this compound under the applied coupling conditions compared to **BH_2_‐Br**. The phenyl‐substituted derivative **C_12_OPh‐BH_2_** was obtained in a low yield of 7%, limited by decomposition of **Br‐BH_2_** during the reaction. DME / H_2_O (10 : 1) at 70°C was not successful and only led to decomposition of **Br‐BH_2_**.

### 2.2 Mesomorphic Properties of Boron *C,N*‐chelates

**FIGURE 1:**
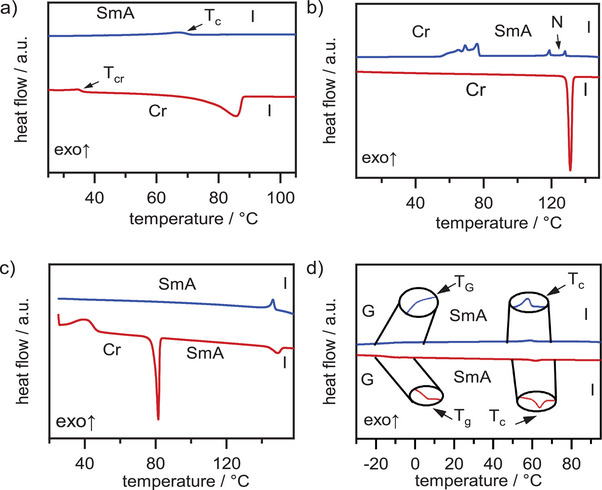
DSC thermograms (heating / cooling rate 10 K / min) of (a) BH_2_‐PhC_4_, (b) BH_2_‐PhOC_4_, (c) BH_2_‐PhOC_12_, (d) BMe_2_‐PhC_3_OCC_7_^F^.

The series of synthesized boron *C,N*‐chelates **BH_2_‐Ar**, **BMe_2_‐Ar**, and **C_12_OPh‐BH_2_** was investigated regarding the mesomorphic properties using polarizing optical microscopy (POM), differential scanning calorimetry (DSC) and small and wide‐angle X‐ray diffraction (SAXS and WAXS). All derivatives with BH_2_ unit exhibited mesomorphic behavior (for details see Supporting Information chapters 3.1–3.3). Both the butyl and butyloxy derivatives **BH_2_‐PhC_4_** and **BH_2_‐PhOC_4_**, displayed mesophases (see ESI Figures S6a,S8a) despite their relatively short chains. **BH_2_‐PhC_4_** exhibited a monotropic mesophase during the cooling cycle, with a transition from the isotropic melt to the mesophase at 71°C. Crystallization was not visible in the second cooling cycle but occurred as cold crystallization in the subsequent heating cycle (Figure 1a). The analogous alkoxy derivative **BH_2_‐PhOC_4_** also showed monotropic behavior but two mesophases were detected (Figure 1b).

The higher temperature phase occurred between 120°C and 129°C, while the lower temperature phase was observed from 78°C to 120°C (Figure 1b). All other hydrogen‐substituted derivatives possessed only one enantiotropic mesophase. A typical DSC curve for the enantiotropic mesophases is exemplary shown for **BH_2_‐PhOC_12_** in Figure 1c. All derivatives with BMe_2_ unit and alkyl or alkoxy chains (**BMe_2_‐Ar**) did not show any mesomorphic behavior (see Supporting Information, Figures S9b, S10, S11). Only the perfluorinated chain was able to induce a broad mesophase at room temperature between ‐10°C and 61°C for **BMe_2_‐PhC_3_OCC_7_^F^** (Figure 1d). The difference in planarity between the hydrogen atoms and the methyl groups on the tetrahedral boron center appears to significantly influence the formation of mesophases.

**FIGURE 2:**
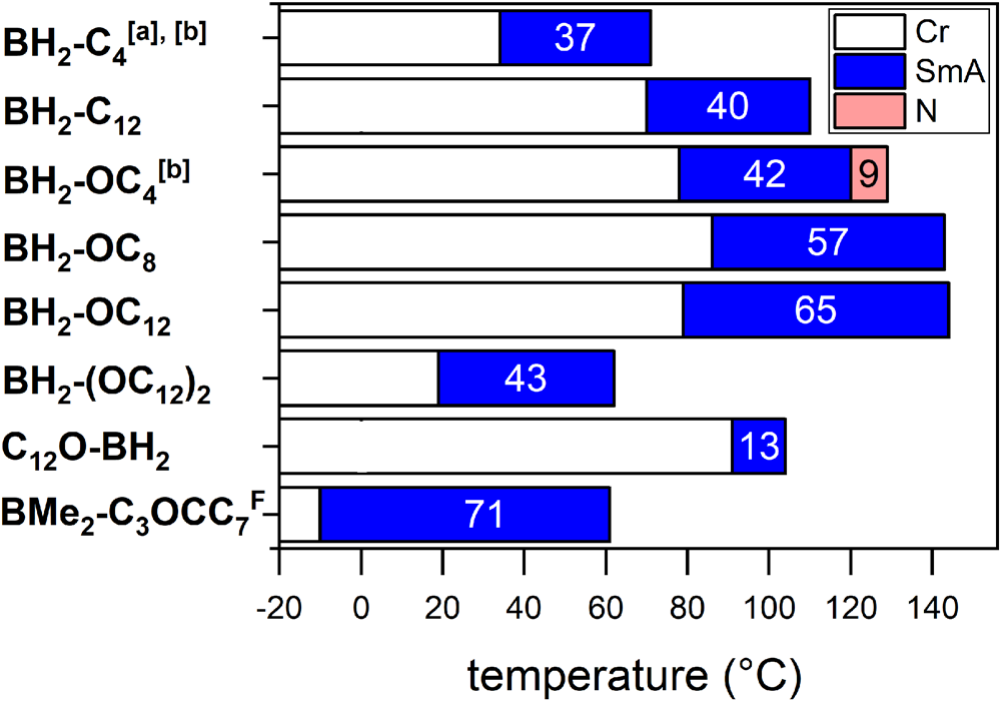
Mesophase widths and phases of calamitic boron *C,N*‐chelates [a] monotropic mesophase, [b] crystallization determined from heating cycle because of cold crystallization.

**FIGURE 3:**
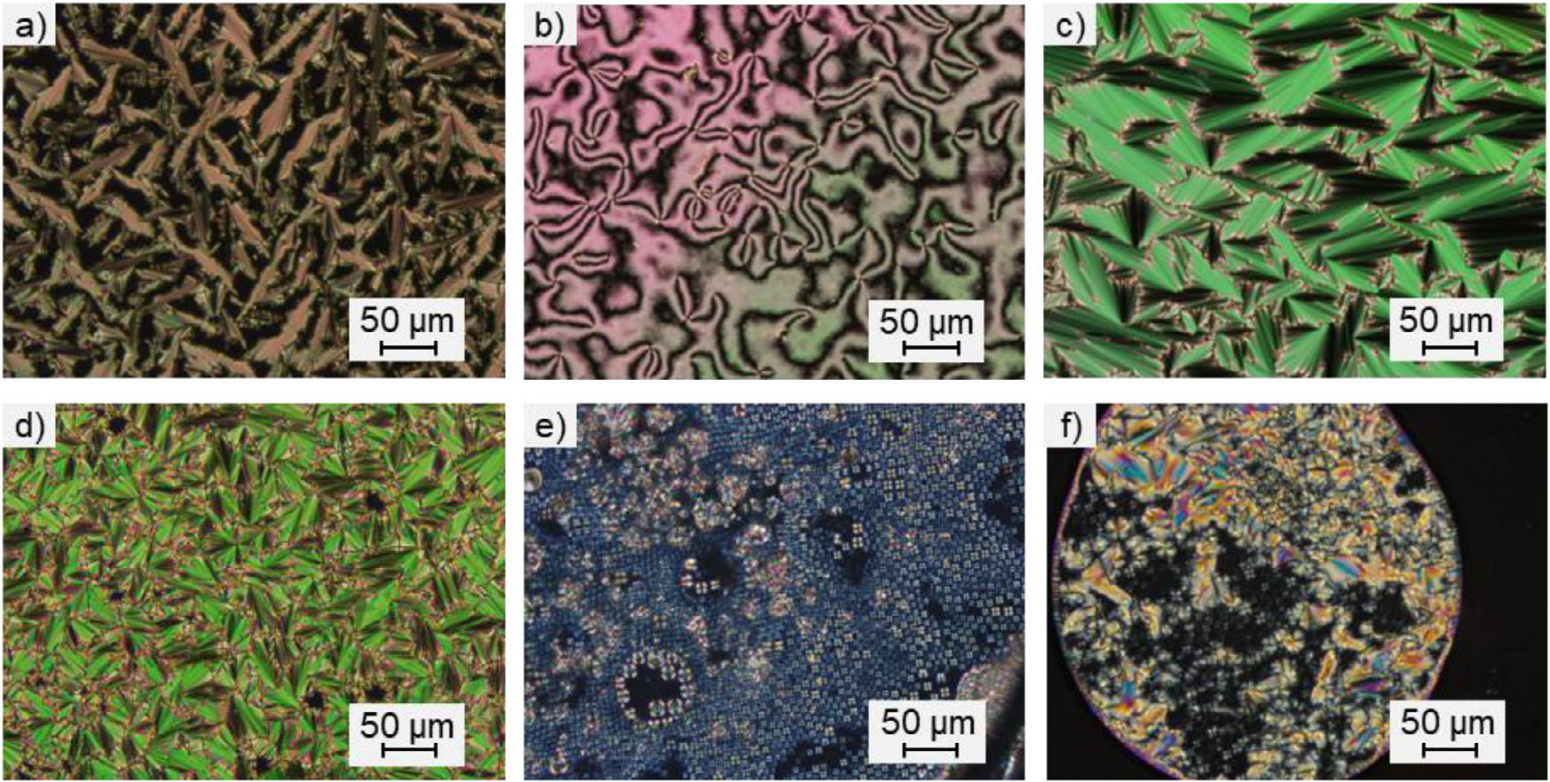
Textures obtained on the POM upon cooling (a) BH_2_‐PhC_4_ (64°C, cooling rate 5 K / min), (b) BH_2_‐PhOC_4_ (130°C, cooling rate 10 K / min), (c) BH_2_‐PhOC_4_ (120°C, cooling rate 10 K / min), (d) BH_2_‐PhOC_12_ (148°C, cooling rate 5 K / min), (e) PhOC_12_‐BH_2_ (106°C, cooling rate 5 K / min), (f) BMe_2_PhC_3_OCC_7_^F^ (50°C, cooling rate 2 K / min).

Investigation via POM showed characteristic fan‐shaped textures that only appear during the cooling for **BH_2_‐PhC_4_**, indicating a monotropic SmA phase (Figure 3a). For **BH_2_‐PhOC_4_**, Schlieren textures with twofold and fourfold defects were observed at higher temperatures, while fan‐shaped textures appeared at lower temperatures during cooling (Figure 2b, c). This is characteristic for a nematic phase above the SmA phase, both monotropic. For all other derivatives **BH_2_‐Ar** (see Supporting Information, Figures S1–S5) the phenyl‐substituted derivative **C_12_OPh‐BH_2_**, and the perfluorinated derivative **BMe_2_‐PhC_3_OCC_7_^F^**, fan‐shaped textures (Figure 3d, f) or Maltese crosses (Figure 3e) were visible under the POM, suggesting the presence of SmA phases in these compounds as well.

The detailed phase widths of all liquid crystalline derivatives (**BH_2_‐Ar, BMe_2_‐PhC_3_OCC_7_^F^,** and **C_12_OPh‐BH_2_**) are shown in Figure 2 (Phase transitions and enthalpies of the whole series are summarized in Supporting Information, Table S2).

Comparison of **BH_2_‐Ar** and **BMe_2_‐Ar** derivatives (Table S2) revealed a strong influence of the substituents H versus Me. While the whole series of **BH_2_‐Ar** derivatives showed liquid crystalline properties, the corresponding **BMe_2_‐Ar** derivatives were nonmesomorphic. Only the compound **BMe_2_‐PhC_3_OCC_7_^F^** with semiperfluorinated side chain showed Sm mesophases. Moreover, **BMe_2_‐PhC_3_OCC_7_^F^** showed the broadest mesophase (71 K, Figure 2) of all derivatives and is also the only one showing liquid crystalline behavior below room temperature. The semi‐perfluorinated chain has a significant influence not only on the presence of a mesophase but also on the phase transition temperatures. Boron C,N‐chelates with a single alkoxy chain showed higher melting and clearing temperatures as the alkyl‐substituted derivatives. Interestingly, **BH_2_‐Ph(OC_12_)_2_** with two alkoxy chains showed significantly lower phase transition temperatures than the compounds that only bear one alkoxy chain (Figure 2). The influence of the chain lengths differed between alkyl‐ and alkoxy‐substituted boron *C,N*‐chelates. Upon increasing the lengths of alkyl side chains the phase range was shifted to higher temperatures, while phase widths were maintained. Thus, **BH_2_‐PhOC_12_** possessed a higher mesophase stability than **BH_2_‐PhOC_4_**. In contrast, increased alkoxy side chain lengths resulted in considerably increased mesophase widths, while the mesophase stability was only slightly increased. In the series **BH_2_‐PhOC_4_** < **BH_2_‐PhOC_8_** < **BH_2_‐PhOC_12_** the SmA phase stability increased at the expense of the nematic phase, which disappeared already for BH_2_ chelate **BH_2_‐PhOC_8_**. When comparing regioisomers **BH_2_‐PhOC_12_** and **C_12_OPh‐BH_2_**, the results in Figure 2 revealed that the position of the aromatic ring with the alkoxy side chain either at the phenyl ring or at the pyridine unit had a large impact on the mesophase stability and temperature range. While **BH_2_‐PhOC_12_** possessed a 65 K wide mesophase with a clearing transition at 144°C, the phase width of **C_12_OPh‐BH_2_** was only 13 K and a lower clearing transition at 119°C was observed.

**FIGURE 4:**
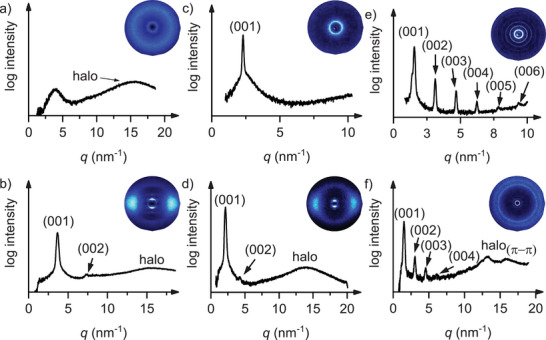
(a) WAXS diffractogram of BH_2_‐PhOC_4_ (N) at 125°C, (b) WAXS diffractogram of BH_2_‐PhOC_4_ (SmA) at 95°C (c) SAXS diffractogram of BH_2_‐PhOC_12_ at 69°C, (d) WAXS diffractogram of BH_2_‐PhOC_12_ at 72°C, (e) SAXS diffractogram of C_12_OPh‐BH_2_ at 95°C, (f) WAXS diffractogram of C_12_OPh‐BH_2_ at 90°C.

SAXS and WAXS measurements were performed to obtain a more detailed understanding of the mesophase geometries and verify the assigned mesophases based on POM observations. The XRD data are summarized in Figure 4 and Table 2. For instance, the SAXS measurement **BH_2_‐PhOC_4_** at 125°C showed a broad small angle reflex (*q*  =  4.06 nm^−1^) and the WAXS measurement showed an additional diffuse halo (*q*  =  15.49 nm [‐1]), consistent with the presence of a nematic phase (Figure 4a). Upon cooling the sample to 95°C, two sharp reflexes appeared at *q*  =  3.51 nm^−1^ (001) and *q*  =  7.83 nm^−1^ (002), confirming the formation of a SmA phase (Figure 4b). Compounds **BH_2_‐PhC_4_**–**BH_2_‐PhOC_12_** similarly exhibited characteristic reflections of SmA phases, in agreement with the POM analysis. An example is shown for **BH_2_‐PhOC_12_** in Figure 4c, d (diffractograms of all compounds are provided in the Supporting Information, Figures S12–S20).

**TABLE 2:** XRD Data of calamitic boron *C,N*‐chelates.

compound	mesophase	*q* / nm^−1^	(*hkl*)	*d*_exp_ / Å	*l* (calc.) [43] / Å
**BH_2_‐PhOC_12_**	SmA at 69°C SmA at 72°C	2.29 4.18^a^ 13.92^a^	(001) (002) halo	27.44 15.03 4.51	26.4
**BH_2_‐PhOC_4_**	N at 125°C SmA at 105°C SmA at 95°C	4.06 15.49^a^ 3.51 7.83^a^ 15.90^a^	(diffuse reflex) (halo) (001) (002) (halo)	15.47 4.06 17.90 8.03 3.95	16.6
**BMe_2_‐PhC_3_OCC_7_^F^**	SmA at 41°C SmA at 45°C	1.20 12.44^a^	(001) (halo)	31.42 5.05	25.3
**C_12_OPh‐BH_2_**	SmA at 95°C SmA at 90°C	1.56 3.12 4.68 6.24 7.86 9.44 13.30^a^ 15.96^a^	(001) (002) (003) (004) (005) (006) halo π‐π distance	40.28 20.14 13.43 10.07 7.99 6.66 4.72 3.94	26.9

^a^ Determined from WAXS measurement.

**C_12_OPh‐BH_2_** displayed six sharp SAXS reflexes from *q*  =  1.56–9.44 nm^−1^, assigned to the (001)‐(006) reflexes of a SmA phase (Figure 4e), in agreement with the observed textures. The WAXS diffractogram showed the characteristic diffuse halo together with an additional reflex attributed to π‐π‐interactions between the aromatic cores (Figure 4f).

**FIGURE 5:**
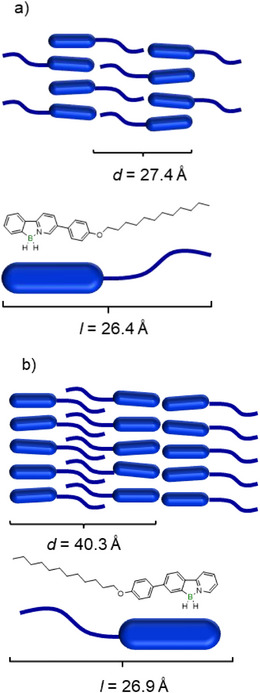
Proposed packing models for (a) a smectic monolayer of **BH_2_‐PhOC_12_**, (b) a smectic bilayer of **C_12_OPh‐BH_2_**.

Calculation of the layer spacing *d* from the diffractogram suggests a monolayer with interdigitating alkoxy chains as a possible packing model for the SmA phase of **BH_2_‐PhOC_12._** The measured spacing of *d*  =  27.4 Å (Figure 5a) is consistent with the calculated molecular length (*l*  =  26.4 Å) [43], assuming nearly complete interdigitation of the alkoxy chains.

The other liquid crystalline derivatives **BH_2_‐Ar** also follow this monolayer packing model. In contrast, **C_12_OPh‐BH_2_** and **BMe_2_‐PhC_3_OCC_7_^F^** exhibited significantly larger spacings (*d*  =  40.3 Å, *d*  =  31.4 Å) than their calculated molecular lengths (*l*  =  26.9 Å, *l*  =  25.3 Å) [43], indicating bilayer formation with partially interdigitated chains illustrated in a proposed packing model (Figure 5b). Especially for **BMe_2_‐PhC_3_OCC_7_^F^** this packing model seems favorable, because the perfluorinated parts of the side chains can form a fluorinated layer.

The larger temperature range and mesophase stability of the regioisomer **BH_2_‐PhOC_12_** as compared to **C_12_OPh‐BH_2_** might be rationalized by additional dipolar interactions between antiparallel oriented adjacent **BH_2_‐PhOC_12_** molecules within the smectic monolayer. In contrast, in the smectic bilayer of **C_12_OPh‐BH_2_** only π−π stacking interactions (Figure 4f) are possible between neighboring molecules.

### 2.3 Photophysical Properties of Boron *C*,*N*‐chelates

**FIGURE 6:**
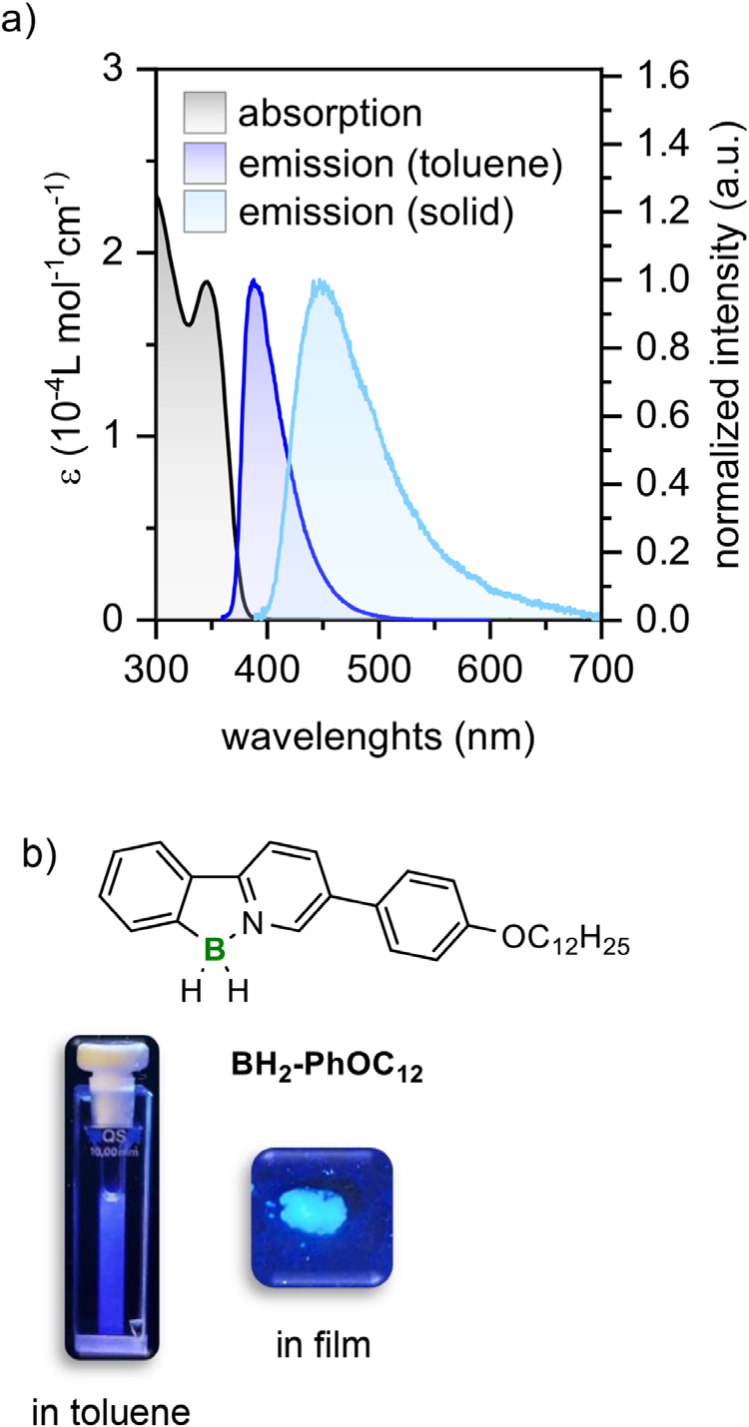
(a) Absorption and emission spectrum BH_2_‐PhOC_12_ (toluene: *c*  =  0.05 mM, *λ*_exc_  =  370 nm, solid state: *λ*_exc_  =  380 nm) and photographs of the emission in the solid and in toluene, (b) photographs of the emission color of BH_2_‐PhOC_12_ in toluene and in the film.

The photophysical properties of the boron *C,N*‐chelates **BH_2_‐Ar, BMe_2_‐Ar,** and **Ar‐BH_2_** were investigated at 23°C in toluene and as films deposited on quartz substrates. In this last case, solid samples were deposited on the substrate, heated up to the isotropic state and cooled down at 23°C. Emission decays were recorded for solutions only, and associated lifetime values were calculated using monoexponential decay models except for **BMe_2_‐PhC_4_** and **BMe_2_‐Ph(OC_12_)_3_** for which a double exponential model was necessary to obtain a valid fit. In these last cases, the average lifetime calculated on the intensity of each emissive component is given. The goodness of fits was validated looking at the χ [2] values and the distribution of residuals. Table 3 gathers all photophysical data deduced from steady state and time resolved experiments. All derivatives show very similar behavior with absorption maxima located between 336 and 352 nm and bright emission in the UV‐blue area in solution with maxima located between 384 nm‐410 nm, exemplarily shown for **BH_2_‐PhOC_12_** in Figure 6. Solvatochromism was exemplary examined for **BH_2_‐PhC_4_** in methylcyclohexane, toluene, dichloromethane, tetrahydrofuran and butyronitrile. Almost no change of the emission was observed with respect to the solvent polarity (for details see Supporting Information, Figure S64). Calculated lifetime values range between 1.8 ns and 2.7 ns and correspond to fluorescence phenomena. This is in line with the small Stokes shift values deduced from the absorption and emission spectra. Associated absolute quantum yield values in toluene solutions vary from 0.61 for **BH_2_‐PhC_12_** to 1 for the brightest emitter **C_12_OPh‐BH_2_**, with most values around 0.8 showing that all compounds are bright emitters in the UV‐blue region. Please note that the accuracy of the AQY measurements is ±10%. Hence, as expected, variations in the type, length, and number of chains had little effect on the photophysical properties, which are primarily determined by the phenyl pyridine‐borane core. Nevertheless, passing from solution to the solid state induces a bathochromic shift of the emission maxima (394 to 473 nm) and broadening of the band which significance is difficult to relate to molecular reason only but might be assessed to differences in the structural arrangement of molecules within the crystalline or liquid crystalline state. Indeed, methylated borane compounds are not liquid crystalline (except **BMe_2_‐PhC_3_OCC_7_^F^**) and show smaller differences (below 2000 cm^−1^) between the emission maximum position in solution and in the solid state, while mesomorphic compounds are much more affected with an energy difference higher than 3300 cm^−1^. Therefore, intermolecular interactions in the mesophase and subsequent crystal phases seem to show a higher tendency at lowering the excited state energy level than interactions in nonliquid crystalline phases. Emission spectra in the mesophase were measured via POM for all liquid crystalline derivatives (Supporting Information, Figures S65–S68 and Table S5). For the whole series a bathochromic shift and broadening of the emission band was observed as compared to the spectra measured in solution. The most pronounced effect was observed for the derivatives with short chain lengths. For example, **BH_2_‐PhOC_4_** possessed emission maxima at *λ*_em_ =  389 nm (toluene), 473 nm (solid) and 537 nm (SmA) respectively, whereas the corresponding emission maxima of **BH_2_‐PhOC_12_** were found at *λ*_em_ =  389 nm (toluene), 447 nm (solid), and 514 nm (SmA). Presumably, the SmA mesophase favors excimer formation as compared to the solid state, which would agree with the experimentally observed π−π distances following the trend: **BH_2_‐PhOC_4_** 3.95 Å (SmA), 4.06 Å (N) < **BH_2_‐PhOC_12_** 4.51 Å (SmA).

**TABLE 3:** Detailed Photophysical properties (absorption maxima *λ*_max_ and emission maxima *λ*_em_ with FWHM_,_ stokes shifts Δ𝑣̅, quantum yields Φ, lifetimes τ, χ [2] and *k* values) of boron *C,N*‐chelates BH_2_‐Ar, BMe_2_‐Ar, Ar‐BH_2_.

compound	λ_max_ (toluene) /nm (ε / mol^−1^ cm^−1^)	λ_em_ (toluene) /nm FWHM / [nm], (cm^−1^)	λ_em_ (solid) /nm FWHM / [nm], (cm^−1^)	Δ𝑣̅ / cm^−^	Φ_tol_ / %	Φ_solid_ / %	τ / ns	χ [2]	*k*_r_ / 10^7^ s^−1^	*k*_nr_ / 10^7^ s^−1^
**BH_2_‐PhC_4_**	342 (17000)	385 [34] (2283)	473 [69] (3485)	3266	66	7	2.33	1.08	28.33	14.59
**BH_2_‐PhC_12_**	338 (13000)	385 [39] (2679)	445 [68] (3377)	3612	61	10	2.27	1.07	26.87	17.18
**BH_2_‐PhOC_4_**	346 (19000)	386 [41] (2577)	444 [77] (3636)	2995	86	8	2.47	1.17	34.82	5.67
**BH_2_‐PhOC_8_**	346 (17000)	391 [39] (2449)	452[86] (4009)	3326	69	10	2.53	0.98	27.38	12.30
**BH_2_‐PhOC_12_**	346 (18000)	389 [41] (2577)	447 [85] (4009)	3195	79	14	2.46	1.01	32.11	8.54
**BH_2_‐Ph(OC_12_)_2_**	346 (16000)	395 [56] (3293)	462 [101] (4423)	3585	75	9	2.73	1.11	27.47	9.16
**C_12_OPh‐BH_2_**	346 (21000)	393 [54] (3285)	473 [107] (4498)	3456	100	10	2.11	1.19	47.14	0.48
**BMe_2_‐PhC_4_** ^a^	336 (18000)	384 [38] (2555)	414 [62] (3477)	3720	77	29	1.79	1.04	43.02	12.85
**BMe_2_‐PhOC_12_**	342 (20000)	385 [40] (2619)	416 [64] (3634)	3266	81	5	2.27	0.92	35.68	8.37
**BMe_2_‐Ph(OC_12_)_2_**	352 (19000)	392 [53] (3241)	417 [60] (3233)	2899	81	50	2.36	1.12	34.32	8.05
**BMe_2_‐Ph(OC_12_)_3_** ^a^	342 (20000)	410 [68] (3942)	431 [71] (3698)	4850	85	70	2.30	1.25	36.96	6.52
**BMe_2_‐PhC_3_OCC_7_^F^**	338 (10000)	384 [39] (2693)	394 [86] (3984)	3544	80	30	2.18	1.15	36.70	9.17

^a^ emission decays were fitted with two components. τ_av_, calculated as τ_av_ = (a_1_τ_1_^2^+a_2_τ_2_^2^)/(a_1_τ_1_+a_2_τ_2_) is given.

The majority of **BH_2_Ar** compounds seem more sensitive to aggregation than the **BMe_2_Ar** one. Indeed, except for **BMe_2_‐PhOC_12_** where AQY passes from 0.81 in solution to 0.05 in the solid state, all other methylated borane compounds show a lower decrease of their emission efficiency when passing from solution to the solid state, while for **BH_2_Ar** compounds, the emission efficiency is drastically reduced in the solid state. This loss in efficiency might be imparted to some aggregation‐caused quenching (ACQ), somehow minimized when the boron atom is substituted by methyl groups instead of protons and when bulky substituents are linked to the emissive core. This is clearly seen for the series **BMe_2_‐Ph(OC_12_)_n_** with *n* = 1, 2, or 3 (*n*  =  number of alkoxy chains) with AQY values in the solid state of 0.05, 0.5, and 0.7, respectively.

Moreover, in case of **BMe_2_‐Ph(OC_12_)_3_** out‐of‐plane rotation of the trisalkoxyphenyl moiety further reduces aggregation‐caused quenching (ACQ) resulting in the highest AQY values for bulk samples within the whole series of tested boron *C,N*‐chelates.

### 2.4 Computational Investigations

To rationalize the photophysical properties, density functional theory (DFT) calculations were performed at the M06‐2X [44]/def2‐TZVP level [45, 46] of theory. The resulting optimized bond lengths and HOMO–LUMO energy gaps for the studied compounds are summarized in Table 4. Although not synthesized in this study, the compounds **BMe_2_‐PhC_4_**, **C_4_OPh‐BH_2_**, and **C_4_OPh‐BMe_2_** were included to maintain comparability across the series.

**TABLE 4:** Obtained B─N and B─C bond lengths in Å of the investigated compounds as well as the respective HOMO‐LUMO gap obtained with M06‐2X in eV.

Compound	B‐N [Å]	B‐C [Å]	HOMO [eV]	LUMO [eV]	HOMO‐LUMO gap [eV]
**BH_2_‐PhC_4_**	1.605	1.599	−7.382	−1.104	6.277
**BMe_2_‐PhC_4_**	1.634	1.610	−7.409	−1.125	6.284
**BH_2_‐PhOC_4_**	1.605	1.599	−7.224	−1.037	6.186
**BMe_2_‐PhOC_4_**	1.632	1.610	−7.240	−1.054	6.186
**BH_2_‐PhOC_12_**	1.605	1.599	−7.209	−1.036	6.173
**C_4_OPh‐BH_2_**	1.603	1.598	−6.911	−1.062	5.849
**C_4_OPh‐BMe_2_**	1.633	1.610	−6.931	−1.075	5.856
**C_12_OPh‐BH_2_**	1.603	1.599	−6.908	−1.050	5.858

**FIGURE 7:**
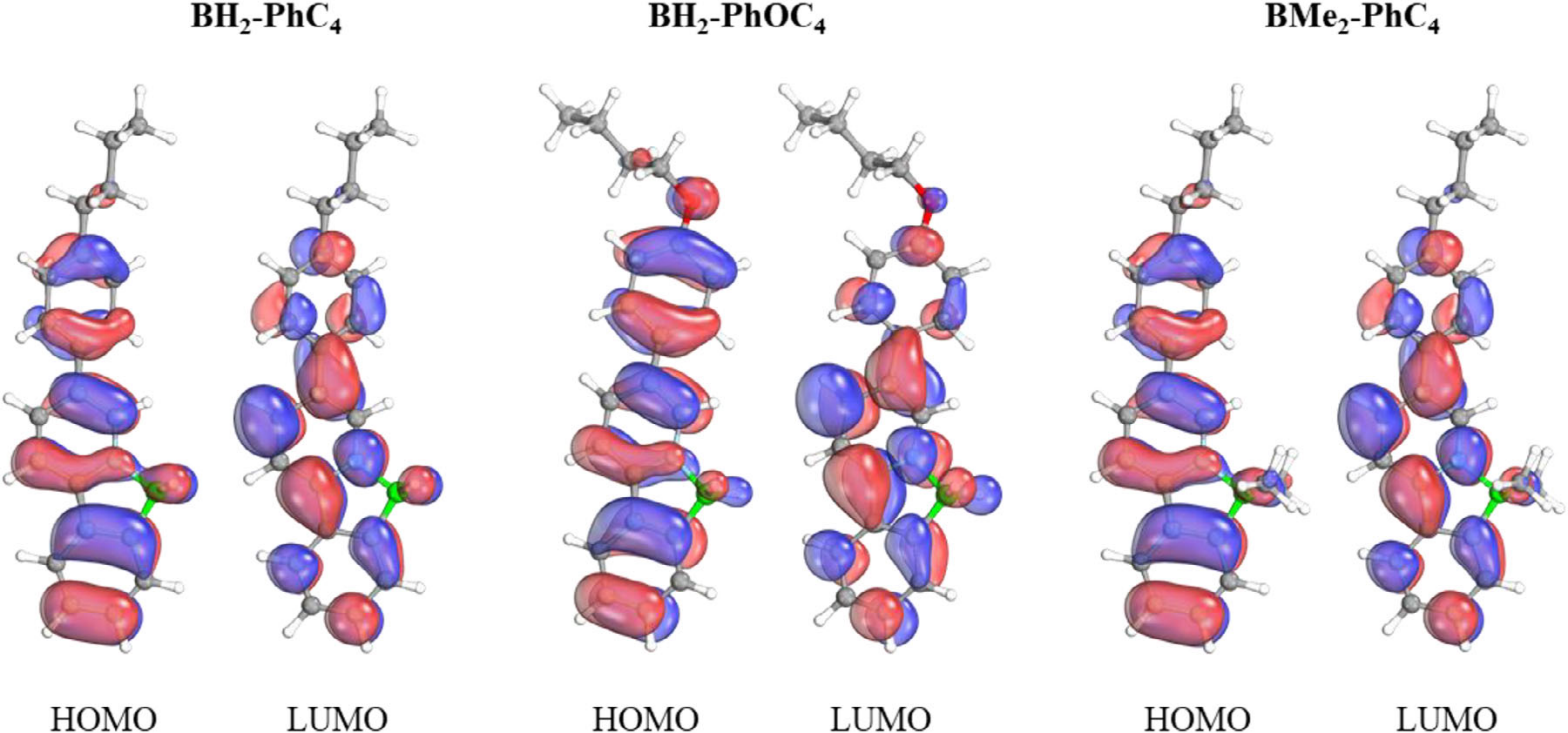
Calculated HOMO and LUMO for the **BH_2_‐PhC_4_**, **BH_2_‐PhOC_4_**, and **BMe_2_‐PhC_4_ s**pecies. H: white, C: grey, O: red, N: blue, B: green.

One of the most notable observations from these investigations is that the HOMO‐LUMO energy gaps remain nearly identical with respect to variations of the substituents at the boron atom (BH_2_ vs. BMe_2_) and the length of the side chain. Variations of side chain type, that is alkyl versus alkoxy resulted in a slight decrease of the HOMO‐LUMO gap by about 0.1 eV. The position of the sidechain, on the other hand, has a notable influence on the HOMO‐LUMO energy gap. The boron *C,N*‐chelates carrying the side chain at the phenyl unit possessed a HOMO‐LUMO gap, which was 0.31–0.34 eV smaller than those of the regioisomeric boron *C,N*‐chelates carrying the side chain at the pyridine unit. Furthermore, we also computed natural bond orbitals at the M06‐2X [44]/def2‐TZVP level [45, 46] level of the **BH_2_‐PhC_4_**, **BH_2_‐PhOC_4_**, and **BMe_2_‐PhC_4_** species. These resulting HOMO and LUMO are displayed in Figure 7. Analysis of these orbitals indicates that, while the different substitutions influence the shape of these orbitals, their influence on the electron density is rather marginal.

Overall, the DFT calculated HOMO‐LUMO gap values of 5.85–6.28 eV correspond to electronic transitions in the UV region (198–212 nm). In contrast, the experimentally measured emission wavelengths are in the range of 384–473 nm, corresponding to blue light emission and thus smaller energy differences for the S_1_ → S_0_ transition. This discrepancy between computed DFT values and experimentally observed emission behavior is typical and well documented in the literature [47, 48]. It can be attributed to several factors including solvent or aggregation effects, exciton formation or involvement of orbitals other than the HOMO‐LUMO transition. [47, 48] Nevertheless, the marginal difference between BH_2_ and BMe_2_ species with different types of side chains is observed in both the computational data and the experimental results.

## 3 Conclusion

A series of boron‐*C*,*N*‐chelates based on phenylpyridine derivatives was synthesized to examine how structural variation affects mesomorphic and photophysical properties. Eleven pyridine‐substituted derivatives (six BH_2_, five BMe_2_) and one phenyl‐substituted BH_2_ analogue were prepared via Suzuki coupling. POM, DSC, and XRD analysis showed that all BH_2_ derivatives formed SmA or N mesophases, whereas the methylated analogues with alkyl or alkoxy chains were nonmesomorphic. Neither increasing chain number nor flexibility led to mesomorphism. Only introduction of a semi‐perfluorinated chain yielded a broad SmA phase at room temperature, highlighting the strong suppressive effect of boron methylation on mesophase formation.

All derivatives exhibited intense blue emission, prompting detailed photophysical characterization by absorption and emission spectroscopy, as well as quantum yield and lifetime measurements. The optical properties in toluene and in the solid state were only modestly influenced by chain type, length, number, or by substitution at boron, indicating that the boron‐*C*,*N* core predominantly dictates their photophysical properties in agreement with DFT calculations. All compounds showed a dominant absorption band between 336–352 nm and strong blue emission in toluene at 384–410 nm. Solid‐state emission underwent a bathochromic shift (394–473 nm) and notable broadening of the emission band. High fluorescence quantum yields of 61–100% were achieved in solution, while solid‐state values decreased due to aggregation‐caused quenching (ACQ).

The results revealed that structural parameters that favored mesomorphism such as the BH_2_ unit rather than BMe_2_ disfavored emission in the bulk, whereas derivatives with good photophysical bulk properties did not show liquid crystalline self‐assembly. These findings have revealed clear structure‐property relationships and provide a foundation for further tuning of boron‐*C*,*N*‐chelates to balance mesomorphic behavior with desirable photophysical performance.

## Author Contributions

F.M. synthesized and characterized the boron *C,N*‐chelates and performed POM, DSC and XRD measurements. F.M. and F.F. performed the photophysical experiments. A.B. and R. S. carried out computational investigations. Y.M. supervised and performed the photophysical experiments. F.M., F.F., and A.Z. checked the data and helped with the manuscript preparation. F.M., A.B., Y.M., J.K., and S.L. wrote the manuscript; S.L. coordinated the research.

## Conflicts of Interest

The authors declare no conflicts of interest.

## Supporting information



The supporting information (SI) contains the experimental procedures, NMR spectra, DSC‐, and XRD‐data, photophysical data, computational details and supplementary experiments.
Additional references cited within the Supporting Information [49, 50, 51, 52, 53, 54, 55, 56, 57, 58, 59, 60, 61, 62, 63, 64, 65, 66, 67].

**Supporting Information File 1**: chem70754‐sup‐0002‐Data.zip.

## Data Availability

The data that support the findings of this study are available in the supplementary material of this article.
