# Supplementary material for: Emissive Liquid Crystalline Boron C,N‐Chelates: Synthesis, Self‐assembly, and Photophysical Properties
Source: Chemistry. 2026 Feb 4;32(15):e03562. doi: 10.1002/chem.202503562 (PMC13107509; doi:10.1002/chem.202503562)
Supplement: Supplementary file 1 — The supporting information (SI) contains the experimental procedures, NMR spectra, DSC‐, and XRD‐data, photophysical data, computational details and supplementary experiments. Additional references cited within the Supporting Information [49, 50, 51, 52, 53, 54, 55, 56, 57, 58, 59, 60, 61, 62, 63, 64, 65, 66, 67]. [file CHEM-32-e03562-s001.docx]

Table of Contents

[1. General Methods S1](#_Toc219893032)

[1.1. Synthesis S1](#_Toc219893033)

[1.2. Mesomorphic properties S2](#_Toc219893034)

[1.3. Photophysical properties S2](#_Toc219893035)

[Sample preparation S2](#_Toc219893036)

[UV/Vis spectroscopy and fluorescence spectroscopy S2](#_Toc219893037)

[Quantum yields S2](#_Toc219893038)

[Emission lifetimes S3](#_Toc219893039)

[2. Synthesis S4](#_Toc219893040)

[2.1. Compounds prepared via literature procedures S4](#_Toc219893041)

[2.2. General procedures S5](#_Toc219893042)

[2.3. Synthesis of BPin-C_3_OCC_7_^F^ S6](#_Toc219893043)

[2.4. Synthesis of boron-*C,N-*chelates S8](#_Toc219893044)

[3. Mesomorphic properties S24](#_Toc219893045)

[3.1. POM textures S24](#_Toc219893046)

[3.2. DSC S26](#_Toc219893047)

[3.3. SAXS and WAXS S29](#_Toc219893048)

[4. Computational Details S35](#_Toc219893049)

[5. Photophysical properties S35](#_Toc219893050)

[5.1. Absorption and Emission S35](#_Toc219893051)

[5.2. Lifetime measurements S47](#_Toc219893052)

[5.3. Solvatochromism S53](#_Toc219893053)

[5.4. Emission in the Mesophase S54](#_Toc219893054)

[6. NMR Spectra S58](#_Toc219893055)

[7. References S114](#_Toc219893056)

1. General Methods
   1. Synthesis

Methods and Chemicals

Commercially available chemicals were used without further purification. Air and moisture sensitive reagents were manipulated by using Schlenk technique under N_2_ atmosphere in dry solvents. Drying of the THF and toluene was performed by distillation over sodium or potassium. Et_2_O was dried by using 4Å molecular sieves. Petroleum ether, EtOAc and CH_2_Cl_2_ were distilled before use.

Column chromatography

Purification via Column chromatography and filtration over silica gel was performed on silica gel (type F60, grain diameter 40-63 μm) from Silicycle. The columns were packed with the specified running agents and the crude products were applied as a solution in the respective running agent mixture or on Celite. Aluminum oxide 90 neutral from Machery-Nagel was used for filtration over neutral aluminum oxide.

Thin layer chromatography (TLC) and column chromatography

Alugram® Xtra SIL G/UV254 prefabricated plates from Machery-Nagel were used to track reaction processes using thin-layer chromatography. The substance stains were detected under UV light (λ= 254, 365 nm).

NMR spectroscopy

The measurement of ^1^H NMR spectra was performed at 300 MHz, 400 MHz, 500 MHz, of ^13^C NMR spectra at 75 MHz, 101 MHz, 176 MHz, of ^11^B NMR spectra at 128 MHz on AVANCE 300, AVANCE 400 and AVANCE 500 spectrometers from Bruker. The chemical shifts δ were given in parts per million (ppm) and referenced to the signal of the respective deuterated solvent.^[49]^ Signal multiplicities were abbreviated as follows: singlet (s), doublet (d), triplet (t), multiplet (m) and given with the respective coupling constants *J* in Hertz (Hz). The signals were assigned using COSY, HSQC and HMBC spectra.

Mass spectroscopy

Mass spectrometry (MS) and high-resolution mass spectrometry (HRMS) were performed using electron impact ionization (EI) on a Finnigan MAT-95 mass spectrometer or using electron spray ionization (ESI) or Liquid Injection Field Desorption ionization (LIFDI) on a Thermo Fisher Scientific Exactive Plus Orbitrap mass spectrometer.

Infrared spectroscopy

IR spectra were recorded using a Bruker Vector 22 FT-IR spectrometer with an MKII Golden Gate Single Reflection Diamond ATR system. The absorption bands are given as whole numbers in wavenumbers 𝑣̃ and their intensities are described as follows: very strong (vs), strong (s), medium (m) weak (w).

- 1. Mesomorphic properties

Polarized optical microscopy (POM)

The mesomorphic properties were examined with a polarized optical microscope from Olympus BX50 with a heating chamber HCS302 and a temperature control unit mK2000B from LN2-P4C. The microphotographs of the textures were taken with a Zeiss Axiocam 105 color camera module and the software ZEN 38. The samples were prepared on regular glass slides.

Differential scanning calorimetry (DSC)

DSC measurements were performed on a DSC3 calorimeter from Mettler-Toledo. Standard 40 µL aluminum crucibles were used for the measurements and the evaluation was carried out with the STARe 16.40 software. The temperatures of the phase transitions are given as onset values of the corresponding peaks and the enthalpies Δ*H* were calc. from the integrals.

Small- and wide-angle Xray diffraction (SAXS and WAXS)

The small- and wide-angle Xray diffraction patterns were recorded temperature dependent (Δ*T* = ±1 K) on a Bruker AXS Nanostar C with a Siemens ceramic tube generator (CuK_α_ radiation, λ = 1.5405 Å, 1500 W) and a Bruker HI-STAR detector. The evaluation of the measurements was carried out with SAXSoffline from Burker, Datasqueeze from Datasqueeze Software and OriginPro 2018 from OriginLab. The diffraction pattern of silver behenate at 25 °C was used for calibration. All samples were prepared in sealed cappilaries by Hildenberg GmbH (external diameter of 0.7 mm, wall thicknes 0.01 mm).

- 1. Photophysical properties

Sample preparation

UV/Vis spectroscopy and fluorescence spectroscopy

Absorption and emission spectra and emission/excitation maps were recorded on a Horiba Jobin Yvon Duetta spectrophotometer at concentrations of *c* = 0.2 – 0.05 mM.

Quantum yields

The absolute quantum yields were measured with a C9920–03 Hamamatsu system.

Emission lifetimes

Lifetime measurements and TRPL mapping at 296 K were realized using a picosecond laser diode (Jobin Yvon deltadiode, 375 nm) and a Hamamatsu C10910-25 streak camera mounted with a slow single sweep unit. Signals were integrated on the whole emission decay. Fits were obtained using origin software and the goodness of fit judged by the reduced χ2 value and residual plot shape.

**Emission in the mesophase**

Measurements of the mesophase emission were carried out on a Nikon 80i POM equipped with a Linkam LTS420E heating table, a Nikon Intensilight source and a ocean optics QE6500 photodetector.

1. Synthesis
   1. Compounds prepared via literature procedures

Table S1: Literature known compounds prepared for this work according to the corresponding literature.

| Substance | Literature |
| --- | --- |
|  | Prepared from 4-(4,4,5,5-tetramethyl-1,3,2-dioxaborolan-2-yl)-phenol according to literature ^[50]^ |
|  | Prepared from 4-(4,4,5,5-tetramethyl-1,3,2-dioxaborolan-2-yl)-phenol according to literature ^[50]^ |
|  | Prepared from 4-(4,4,5,5-tetramethyl-1,3,2-dioxaborolan-2-yl)-phenol according to literature ^[50]^ |
|  | Prepared from 4-dodecylaniline according to literature ^[51]^ |
|  | Prepared from 4-butylaniline according to literature ^[51]^ |
|  | Prepared from 5-bromosalicylaldehyde according to literature ^[52]^ |
|  | Prepared from **10** according to literature ^[53]^ |
|  | Prepared from **11** according to literature ^[52]^ |
|  | Prepared from 2,3,4-(trimethoxy)-bromobenzene according to literature ^[54]^ |
|  | Prepared from **12** according to literature ^[55]^ |
|  | Prepared from **13** according to literature ^[56]^ |
|  | Prepared from (Perfluoroheptyl)methanol over two steps  according to literature ^[57]^ |

- 1. General procedures

General procedure for the synthesis of phenylpyridine‑dibromborane complexes (BBr_2_-Br, Br-BBr_2_) (GP1)

According to an adapted literature procedure ^[11]^ the corresponding 2-phenylpyridine (**ppy-Br, Br-ppy**) (600 mg, 2.56 mmol, 1 eq.) and *i*Pr_2_NEt (440 µL, 2.56 mmol, 1 eq.) were dissolved in CH_2_Cl_2_ (50 mL). BBr_3_ (730 µL, 7.69 mmol, 3 eq.) was slowly added at 0 °C. The solution was warmed up to ambient temperature and stirred for 18 h. The reaction was quenched by the addition of saturated aqueous K_2_CO_3_-solution (10 mL) and deion. H_2_O (10 mL). The aqueous phase was extracted with CH_2_Cl_2_ (2 × 20 mL), the combined organic layers were washed with deion. H_2_O (25 mL) and brine (25 mL) and dried over MgSO_4_. The solvent was removed under reduced pressure. The crude product was washed with petroleum ether (50 mL).

General procedure for the synthesis of phenylpyridine-boronhydride complexes (BH_2_-Br, Br-BH_2_) (GP2)

According to an adapted literature procedure ^[11]^ the corresponding phenylpyridine‑dibromborane complex (**BBr_2_-Br, Br-BBr_2_**) (800 mg, 1.98 mmol, 1 eq.) was suspended in Et_2_O (50 mL). LiAlH_4_ (158 mg, 4.16 mmol, 2.1 eq.) was slowly added in small portions at 0°C and the suspension was stirred at this temperature for 2 h. The reaction was quenched by the addition of deion. H_2_O (10 mL) and the aqueous phase was extracted with EtOAc (2 x 20 mL). The combined organic layers were washed with brine (50 mL), dried over MgSO_4_ and the solvent was removed under reduced pressure. Further purification of the crude products can be found in the respective derivatives.

General procedure for the synthesis of Suzuki-coupled phenylpyridine-borane complexes (BH_2_-Ar, BMe_2_-Ar, Ar-BH_2_) (GP3)

**BH_2_-Br, BMe_2_-Br** or **Br-BH_2_** (60 mg, 264 µmol, 1 eq.), **BPin-Ar** (64 mg, 246 µmol, 1 eq.) and Cs_2_CO_3_ (239 mg, 732 µmol, 3 eq.) were dissolved in degassed toluene / H_2_O / EtOH (10 mL / 1 mL / 1). Pd(PPh_3_)_4_ (28 mg, 24 µmol, 0.05 eq.) was added and the reaction mixture was stirred for 18 h at 50 °C. After removal of the solvent under reduced pressure the residue was dissolved in CH_2_Cl_2_ (20 mL) and deion. H_2_O (10 mL). The aqueous phase was extracted with CH_2_Cl_2_ (3 × 20 mL) and the combined organic layers were washed with brine (25 mL), dried over MgSO_4_ and filtered over neutral aluminum oxide. The crude products were further purified via recrystallization.

- 1. Synthesis of BPin-C_3_OCC_7_^F^

1-Bromo-4-(3-((2,2,3,3,4,4,5,5,6,6,7,7,8,8,8-pentadecafluorooctyl)oxy)prop-1-yn-1-yl)benzene (15)

A Schlenk tube was charged with 4-bromoiodbenzene (1.5 g, 5.3 mmol, 1 equiv.), CuI (60.59 mg, 0.318 mmol, 0.06 equiv.) and Pd(PPh_3_)_2_Cl_2_ (126 mg, 0.16 mmol, 0.03 equiv.). Abs. Triethylamine (40 mL) was added, followed by the addition of the alkyne **14** (2.51 g, 5.73 mmol, 1.08 equiv.). The mixture was stirred for 3 h at 80 °C. petroleum ether (40 mL) was added to the mixture, the mixture was filtered over celite (eluent: petroleum ether) and the volatiles were removed under reduced pressure. The resulting crude was purified via column chromatography on silica (petroleum ether / EtOAc = 10 : 1). **15** was obtained as a colorless solid (2.05 g, 3.45 mmol, 65%).

^1^H NMR (700 MHz, CDCl_3_): δ = 3.59 – 4.27 (m, 2H, 8-H), 4.52 (s, 2H, 7-H), 7.31 (d, *J* = 8.5 Hz, 2H, 3-H), 7.47 (d, *J* = 8.5 Hz, 2H, 2-H) ppm.

^13^C NMR (176 MHz, CDCl_3_): δ = 60.4 (C-7), 66.1 (t, C-8),84.1 (C-6), 87.1 (C-5), 108.4 - 119.7 (m, CF_2_ and CF_3_), 121.1 (C-4), 123.4 (C-1), 131.9 (C-2), 133.4 (C-3) ppm.

^19^F NMR (376 MHz, CDCl_3_): δ = -126.28 – -125.86 (m, 2F, CF_2_), -123.42 – -123.02 (m, 2F, CF_2_), -122.85 – -122.45 (m, 2F, CF_2_), -122.13 – -121.83 (m, 3F, CF_3_), -119.37 – -119.11 (m, 2F, CF_2_), -80.86 – -80.63 (m, 2F, CF_2_) ppm.

FT-IR (ATR): $\tilde{\nu}$ = 1486 (w), 1359 (w), 1236 (s), 1200 (s), 1143 (s), 1072 (w), 1058 (w), 1012 (s), 824 (s), 737 (w), 711 (w), 701 (w), 660 (w), 563 (w), 524 (s) cm^-1^.

HRMS (LIFDI): calculated for C_17_H_8_Br_1_F_15_O_1_^+^: 591.9514 [M^+^]; found: 591.9508.

4,4,5,5-Tetramethyl-2-(4-(3-((2,2,3,3,4,4,5,5,6,6,7,7,8,8,8-pentadecafluorooctyl)oxy)-propyl)phenyl)-1,3,2-dioxaborolane (BPin-C_3_OCC_7_^F^)

Compound **15** (1.93 g, 3.25 mmol, 1 equiv.) was placed in a Schlenk tube. Abs THF (60 mL) was added, and the solution was cooled to -90 °C before *n*-BuLi (1.37 mL, 3.42 mmol, 1.05 equiv.) was added. After stirring for 15 min at ‑90 °C, 2-Methoxy-4,4,5,5-tetramethyl-1,3,2-dioxaborolan (1.03 g, 1.07 mL, 6.51 mmol, 2 equiv.) was added, the mixture was quickly warmed to room temperature and stirred for additional 75 min. After that the reaction was quenched by addition of an aqueous solution of NH_4_Cl (50 mL). Subsequently, the mixture was extracted with Et_2_O (3x 30 mL) and washed with brine (50 mL). The resulting crude product was filtered over silica (eluent: petroleum ether / EtOAc = 5 : 1). The resulting intermediate and Pd on activated charcoal (158 mg, 144.49 mmol, 0.05 equiv., 10 wt% Pd) were suspended in a mixture of EtOAc (20 mL) and EtOH (5 mL). The mixture was stirred for 4 days under H_2_ atmosphere (balloon, 1 atm) at room temperature. before being filtered over silica (eluent: EtOAc). The solvents were removed under reduced pressure, and the crude product was purified by column chromatography (silica, petroleum ether / EtOAc = 20 : 1) to obtain **BPin-C_3_OCC_7_^F^** as colorless solid (0.91 g, 1.41 mmol, 43%).

^1^H NMR (700 MHz, CDCl_3_): δ = 1.34 (s, 12H, 10-H) , 1.75 – 2.09 (m, 2H, 6-H), 2.42 – 2.91 (m, 2H, 5-H), 3.57 (t, *J* = 6.3 Hz, 2H, 7-H), 3.70 – 4.34 (m, 2H, 8-H),7.19 (d, *J* = 8.0 Hz, 2H, 3-H), 7.74 (d, *J* = 7.9 Hz, 2H, 2-H) ppm.

^13^C NMR (176 MHz, CDCl_3_): δ = 31.1 (C-6), 25.0 (C-10), 32.2 (C-5), 68.0 (t, C-8), 72.2 (C-7), 83.8 (C-9), 106.7 - 120.0 (m, CF_2_ and CF_3_), 126.5 (C-1), 128.1 (C-3), 135.1 (C-2), 145.0 (C-4) ppm.

^19^F NMR (376 MHz, CDCl_3_): δ = -126.66 – -125.89 (m, 2F, CF_2_), -124.65 – -123.00 (m, 2F, CF_2_), -122.90 – -122.60 (m, 2F, CF_2_), -122.41 – -121.64 (m, 3F, CF_3_), -120.20 – -118.98 (m, 2F, CF_2_), -80.95 – -80.70 (m, 2F, CF_2_) ppm.

^11^B NMR (128 MHz, CDCl_3_): δ = 30.7 ppm.

FT-IR (ATR): $\tilde{\nu}$ = 2981 (w), 2932 (w), 2880 (w), 1612 (w), 1400 (w), 1361 (s), 1321 (w), 1239 (s), 1205 (s), 1142 (s), 1089 (s), 1055 (w), 1021 (w), 962 (w), 908 (s), 859 (w), 733 (s), 710 (w), 658 (s), 563 (w), 528  (w) cm^-1^.

HRMS (LIFDI): calculated for C_23_H_24_B_1_F_15_O_3_^+^: 644.1578 [M^+^]; found: 644.1574.

- 1. Synthesis of boron-*C,N-*chelates

3,6,6-Tribromo-6*H*-5λ^4^,6λ^4^-benzo[3,4][1,2]azaborolo[1,5-a]pyridine (BBr_2_-Br)

Preparation according to GP1. The crude product was washed with petroleum ether (50 mL). Yield: off-white solid (796 mg, 1.97 mmol, 77 %).

^1^H NMR (400 MHz, CDCl_3_): δ = 7.38–7.46 (m, 1H, 8-H), 7.55–7.63 (m, 1H, 9-H), 7.70–7.76 (m, 1H, 10-H), 7.77–7.83 (m, 1H, 2-H), 7.83–7.89 (m, 1H, 7-H), 8.20–8.27 (m, 1H, 3-H), 9.00–9.05 (m, 1H, 5-H) ppm.

^11^B NMR (128 MHz, CDCl_3_): δ = -1.1 ppm.

^13^C NMR was not measured due to low solubility of the compound.

M.p.: 210 °C (decomposition).

HRMS (EI): *m/z* for C_11_H_6_BBr_3_N^-^ calc.: 401.8118 [M‑H]^‑^, found: 401.8119, for C_11_H_7_BBr_2_N calc.: 323.9015 [M‑Br]^+^, found: 323.9011.

FTIR (ATR): 𝑣̃ = 3056 (w), 2325 (vs), 2256 (w), 1611 (s), 1479 (vs), 1454 (m), 1446 (m), 1387 (m), 1326 (m), 1287 (w), 1238 (w), 1155 (m), 1134 (w), 1108 (m), 1077 (w), 1044 (m), 1012 (m), 834 (m), 769 (vs), 724 (vs), 673 (m), 459 (w) cm^−1^.

The spectral data match those reported in the literature.^[58]^

**6,6,8-Tribromo-6*H*-5λ^4^,6λ^4^-benzo[3,4][1,2]azaborolo[1,5-a]pyridine (Br-BBr_2_)**

Preparation according to GP1. The crude product was washed with petroleum ether (50 mL). Yield: off-white solid (796 mg, 1.97 mmol, 55 %).

^1^H NMR (400 MHz, CDCl_3_): δ = 7.51–7.61 (m, 1H, 4-H), 7.57–7.65 (m, 2H, 7-H, 8-H), 7.87–7.93 (m, 1H, 2-H), 7.97–8.02 (m, 1H, 3-H), 8.14–8.23 (m, 1H, 10-H), 8.91–8.97 (m, 1H, 5‑H) ppm.

^11^B NMR (128 MHz, CDCl_3_): δ = -1.8 ppm.

^13^C NMR was not measured due to low solubility of the compound.

M.p.: 192 °C (decomposition)

HRMS (EI): *m/z* for C_11_H_7_BBr_2_N^-^ calc.: 321.9038 [M‑Br]^‑^, found: 321.9033.

FTIR (ATR): 𝑣̃ = 3056 (w), 2920 (w), 2850 (w), 2833 (w), 2146 (w), 2008 (w), 1625 (w), 1594 (w), 1487 (m), 1464 (w), 1440 (w), 1308 (w), 1173 (w), 1134 (w), 880 (w), 834 (w), 790 (w), 763 (w), 728 (vs), 650 (s), 551 (w), 487 (w), 445 (w), 420 (w) cm^−1^.

The spectral data match those reported in the literature.^[59]^

**3-Bromo-6*H*-5λ^4^-benzo[3,4][1,2]azaborolo[1,5-a]pyridine (BH_2_-Br)**

Preparation according to GP2. The crude product was purified via column chromatography on silica (elutent: petroleum ether / EtOAc = 20 : 1) and recrystallized from *i*PrOH / EtOAc (10 : 1). Yield: colorless needles (245 mg, 996 mmol, 50 %).

^1^H NMR (400 MHz, CDCl_3_): δ = 3.48 (d, *J* = 167.6 Hz, 2H, BH_2_), 7.29–7.37 (m, 1H, 8‑H), 7.43–7.51 (m, 1H, 9-H), 7.75–7.81 (m, 1H, 10-H), 7.81–7.89 (m, 2H, 7-H, 2-H), 8.03–8.10 (m, 1H, 3-H), 8.73–8.77 (m, 1H, 5-H) ppm.

^13^C NMR (75 MHz, CDCl_3_): δ = 116.0 (C‑4), 118.8 (C‑2), 121.8 (C‑7), 125.6 (C‑8), 130.4 (C‑10), 130.8 (C‑9), 136.0 (C‑6), 142.1 (C‑3), 145.3 (C‑5), 157.5 (C‑1) ppm. The carbon atom next to the boron atom was not observed in the ^13^C NMR-spectrum due to quadrupole relaxation.

^11^B NMR (128 MHz, CDCl_3_): δ = -8.0 ppm.

M.p.: 120 °C.

HRMS (EI): *m/z* for C_11_H_8_BBrN^-^ calc.: 243.9930 [M‑H]^‑^, found: 243.9930.

FTIR (ATR): 𝑣̃ = 3050 (w), 2917 (w), 2849 (w),2162 (w), 2027 (w), 2010 (w), 1959 (w), 1613 (w), 1483 (m), 1446 (w), 1384 (w), 1302 (w), 1172 (w), 1112 (w), 1069 (w), 853 (w), 842 (w), 815 (w), 759 (w), 729 (vs), 644 (s), 473 (w), 417 (w) cm^−1^.

**8-Bromo-6*H*-5λ^4^-benzo[3,4][1,2]azaborolo[1,5-a]pyridine (Br-BH_2_)**

Preparation according to GP2. The crude product was recrystallized from *i*PrOH / EtOAc (10 : 1). Yield: colorless solid (128 mg, 520 µmol, 41 %).

^1^H NMR (400 MHz, CDCl_3_): δ = 2.95–3.89 (m, 2H, BH_2_), 7.32–7.40 (m, 1H, 4-H), 7.41–7.48 (m, 1H, 8-H), 7.68–7.74 (m, 1H, 7-H), 7.89–8.02 (m, 3H, 10-H, 2-H, 3-H), 8.59–8.65 (m, 1H, 5-H) ppm.

^13^C NMR (75 MHz, CDCl_3_): δ = 118.2 (C‑2), 121.3 (C‑4), 123.0 (C‑7), 126.3 (C‑9), 128.5 (C‑8), 133.3 (C‑10), 135.4 (C‑6), 139.5 (C‑3), 144.1 (C‑5), 157.8 (C‑1) ppm. The carbon atom next to the boron atom was not observed in the ^13^C NMR-spectrum due to quadrupole relaxation.

^11^B NMR (128 MHz, CDCl_3_): δ = -8.3 ppm.

M.p.: 127 °C

HRMS (EI): *m/z* for C_11_H_8_BBrN^-^ calc.: 243.9941 [M‑H]^‑^, found: 243.9931.

FTIR (ATR): 𝑣̃ = 2342 (s), 2268 (m), 1624 (vs), 1593 (m), 1567 (m), 1548 (w), 1479 (vs), 1461 (m), 1430 (m), 1395 (m), 1324 (m), 1308 (m), 1261 (m), 1155 (s), 1126 (w), 1106 (m), 1087 (w), 1063 (s), 1036 (w), 816 (s), 765 (vs), 738 (m), 469 (w), 408 (w) cm^−1^.

**3-Bromo-6,6-dimethyl-6*H*-5λ^4^,6λ^4^-benzo[3,4][1,2]azaborolo[1,5-a]pyridine (BMe_2_-Br)**

**BBr_2_-Br** (250 mg, 619 µmol, 1 eq.) was dissolved in abs. toluene (15 mL). Me_3_Al (680 µL, 1.36 mmol, 2M in toluene, 2.2 eq.) was added dropwise at ambient temperature and the reaction was stirred for 5 min. Saturated aqueous Rochelle salt solution (10 mL) was added at 0 °C to stop the reaction. After stirring for 1 h, the aqueous phase was extracted with EtOAc (2 × 25 mL), the combined organic layers were washed with brine (25 mL) and dried over MgSO_4_. After removal of the solvent under reduced pressure the product was obtained was a colorless solid (136 mg, 496 µmol, 80 %).

^1^H NMR (300 MHz, CDCl_3_): δ = 0.06 (s, 6H, CH_3_), 7.24–7.35 (m, 1H, 9-H), 7.39–7.50 (m, 1H, 10-H), 7.59–7.67 (m, 1H, 2-H), 7.77–7.87 (m, 2H, 8-H, 3-H), 8.01–8.10 (m, 1H, 7-H), 8.48–8.55 (m, 1H, 5-H) ppm.

^13^C NMR (75 MHz, CDCl_3_): δ = 116.6 (C‑4), 118.8 (C‑2), 121.7 (C‑7), 125.5 (C‑9), 129.4 (C‑8), 130.9 (C‑10), 134.4 (C‑6), 142.3 (C‑3), 143.9 (C‑5), 156.0 (C‑1) ppm. The carbon atom C-11 next to the boron atom and the CH_3_ groups were not observed in the ^13^C NMR-spectrum due to quadrupole relaxation.

^11^B NMR (128 MHz, CDCl_3_): δ = 2.3 ppm.

M.p.: 193 °C

HRMS (EI): *m/z* for C_13_H_13_BBrN^-^ calc.: 272.0246 [M‑H]^‑^, found: 272.0246.

FTIR (ATR): $\tilde{\nu}$ = 3054 (w), 2918 (s), 2886 (m), 2850 (w), 2823 (m), 2173 (w), 2136 (w), 1979 (w), 1904 (w), 1612 (m), 1478 (s), 1446 (m), 1430 (w), 1384 (m), 1326 (m), 1284 (m), 1272 (m), 1174 (m), 1157 (w), 1137 (w), 1102 (m), 1056 (m), 1028 (m), 946 (m), 908 (m), 867 (w), 842 (w), 822 (m), 770 (s), 734 (s), 726 (s), 656 (w), 547 (w), 528 (w), 496 (w), 453 (w), 420 (m) cm^-1^.

The spectral data match those reported in the literature.^[60]^

3-(4-Butylphenyl)-6*H*-5λ^4^-benzo[3,4][1,2]azaborolo[1,5-a]pyridine (BH_2_-PhC_4_)

Prepared according to GP3. The crude product was recrystallized from petroleum ether / EtOAc (10 / 1). **BH_2_-PhC_4_** was obtained as a colorless solid (25 mg, 84 µmol, 21 %).

^1^H NMR (700 MHz, CDCl_3_): δ = 0.92–0.98 (m, 3H, CH_3_), 1.36–1.43 (m, 2H, CH_3_C*H*_2_), 1.61–1.68 (m, 2H, CH_3_CH_2_C*H*_2_), 2.66–2.71 (m, 2H, CH_3_CH_2_CH_2_C*H*_2_), 3.51 (d, *J* = 102.6 Hz, 2H, BH_2_), 7.31–7.35 (m, 3H, 8-H, 14-H), 7.43–7.47 (m, 1H, 9-H), 7.52–7.56 (m, 2H, 13-H), 7.79–7.82 (m, 1H, 10-H), 7.86–7.90 (m, 1H, 7-H), 7.98–8.02 (m, 1H, 2-H), 8.13–8.17 (m, 1H, 3-H), 8.86 (d, *J* = 2.3 Hz, 1H, 5-H) ppm.

^13^C NMR (176 MHz, CDCl_3_): δ = 14.1 (CH_3_), 22.5, 33.7, 35.5 (CH_2_), 118.0 (C-2), 121.6 (C-7), 125.4 (C-14), 126.8 (C-13), 129.7 (C-8), 130.3 (C-10), 130.4 (C-9), 133.1 (C-15), 134.9 (C-4), 136.7 (C-6), 137.7 (C-3), 142.1 (C-5), 144.2 (C-12), 156.9 (C-1) ppm. The carbon atom C-11 next to the boron atom was not observed in the ^13^C NMR spectrum due to quadrupole relaxation.

^11^B NMR (128 MHz, CDCl_3_): δ = -7.5 ppm.

M.p. (DSC): 52 °C

HRMS (EI): *m/z* for C_21_H_21_BN^‑^ calc.: 298.1765 [M‑H]^-^, found: 298.1764

FTIR (ATR): 𝑣̃ = 2956 (s), 2923 (vs), 2854 (s), 2344 (s), 1642 (m), 1622 (s), 1487 (vs), 1454 (s), 1385 (m), 1328 (m), 1240 (m), 1153 (m), 1053 (w), 1012 (m), 820 (s), 773 (s), 732 (vs), 677 (m) cm^−1^.

Absorption (toluene, *c* = 0.05 mM): λ_max_ = 342 nm (ε_λ_ = 17000 L mol^-1^ cm^-1^).

Emission (toluene, λ_exc_ = 370 nm): λ_em_ = 385 nm (FWHM = 34 nm).

Emission (solid state, λ_exc_ = 370 nm): λ_em_ = 437 nm (FWHM = 69 nm).

3-(4-Dodecylphenyl)-6*H*-5λ^4^-benzo[3,4][1,2]azaborolo[1,5-a]pyridine (BH_2_-PhC_12_)

Prepared according to GP3. No further purification of the crude product was necessary. **BH_2_‑PhC_12_** was obtained as a colorless solid (41 mg, 100 µmol, 49 %).

^1^H NMR (700 MHz, CDCl_3_): δ = 0.83–0.93 (m, 3H, CH_3_), 1.19–1.43 (m, 18H, CH_2_), 1.63–1.70 (m, 2H, ArCH_2_C*H*_2_), 2.63–2.74 (m, 2H, ArCH_2_), 3.21–3.78 (m, 2H, BH_2_), 7.29–7.37 (m, 3H, 8-H, 14-H), 7.40–7.51 (m, 1H, 9-H), 7.50–7.60 (m, 2H, 13-H), 7.76–7.85 (m, 1H, 10-H), 7.84-7.92 (m, 1H, 7-H), 7.96–8.05 (m, 1H, 2-H), 8.11–8.20 (m, 1H, 3-H), 8.86 (d, *J* = 2.0 Hz, 1H, 5-H) ppm.

^13^C NMR (176 MHz, CDCl_3_): δ = 14.3 (CH_3_), 22.8, 29.4, 29.5, 29.6, 29.7, 29.8, 29.8, 29.8, 29.8, 31.5, 32.1, 35.8 (CH_2_), 118.0 (C‑2), 121.6 (C‑7), 125.4 (C‑14), 126.8 (C‑13), 129.6 (C‑8), 130.3 (C‑10), 130.4 (C‑9), 133.1 (C‑15), 134.9 (C‑4), 136.7 (C‑6), 137.7 (C‑3), 142.1 (C‑5), 144.2 (C‑12), 156.9 (C‑1) ppm. The carbon atom C-11 next to the boron atom was not observed in the ^13^C NMR spectrum due to quadrupole relaxation.

^11^B NMR (128 MHz, CDCl_3_): δ = -8.0 ppm.

M.p. (DSC): 65 °C

HRMS (EI): *m/z* for C_29_H_37_BN^-^ calc.: 410.3030 [M‑H]^‑^, found: 410.3020.

FTIR (ATR): $\tilde{\nu}$ = 3041 (w), 2955 (m), 2922 (vs), 2852 (s), 2349 (w), 2046 (w), 2016 (w), 1980 (w), 1622 (m), 1486 (s), 1466 (m), 1455 (m), 1384 (w), 1327 (m), 1282 (w), 1239 (w), 1153 (w), 1078 (w), 1053 (w), 1035 (w), 1012 (w), 919 (w), 819 (m), 775 (m), 732 (m), 677 (w), 582 (w), 542 (w), 464 (w), 409 (w) cm^-1^.

Absorption (toluene, *c* = 0.04 mM): λ_max_ = 338 nm (ε_λ_ = 13000 L mol^-1^ cm^-1^).

Emission (toluene, λ_exc_ = 360 nm): λ_em_ = 385 nm (FWHM = 39 nm).

Emission (solid state, λ_exc_ = 370 nm): λ_em_ = 445 nm (FWHM = 68 nm

3-(4-(Dodecyloxy)phenyl)-6*H*-5λ^4^-benzo[3,4][1,2]azaborolo[1,5-a]pyridine (BH_2_-PhOC_12_)

Preparation according to GP3. The crude product was recrystallized from petroleum ether / EtOAc (10 / 1). **BH_2_-PhOC_12_** was obtained as a colorless solid (36 mg, 147 µmol, 36 %).

^1^H NMR (700 MHz, CDCl_3_): δ = 0.86–0.91 (m, 3H, CH_3_), 1.25–1.38 (m, 16H, CH_2_), 1.45–1.52 (m, 2H, OCH_2_CH_2_C*H*_2_), 1.79–1.85 (m, 2H, OCH_2_C*H*_2_), 3.28–3.75 (m, 2H, BH_2_), 4.00–4.04 (m, 2H, OCH_2_), 7.01–7.05 (m, 2H, 14-H), 7.30–7.35 (m, 1H, 8-H), 7.42–7.47 (m, 1H, 9-H), 7.53–7.56 (m, 2H, 13-H), 7.78–7.82 (m, 1H, 10-H), 7.85–7.88 (m, 1H, 7-H), 7.96–8.00 (m, 1H, 2-H), 8.09–8.13 (m, 1H, 3-H), 8.81–8.84 (m, 1H, 5-H) ppm.

^13^C NMR (176 MHz, CDCl_3_): δ = 14.3 (CH_3_), 22.8, 26.2, 29.3, 29.5, 29.5, 29.7, 29.7, 29.8, 29.8, 32.1 (CH_2_), 68.4 (OCH_2_), 115.5 (C-14), 117.9 (C-2), 121.5 (C-7), 125.4 (C-8), 127.9 (C-15), 128.1 (C-13), 130.2 (C-9), 130.4 (C-10), 134.6 (C-4), 136.8 (C-6), 137.3 (C-3), 141.8 (C-5), 156.5 (C-1), 160.1 (C-12) ppm. The carbon C-11 atom next to the boron atom was not observed in the ^13^C NMR spectrum due to quadrupole relaxation.

^11^B NMR (128 MHz, CDCl_3_): δ = -8.1 ppm.

M.p. (DSC): 79 °C

HRMS (EI): *m/z* for C_29_H_37_BNO^-^ calc.: 426.2968 [M‑H]^‑^, found: 426.2964.

FTIR (ATR): 𝑣̃ = 3042 (w), 2921 (vs),2852 (s), 2348 (w), 2092 (w), 1950 (w), 1620 (m), 1606 (s), 1519 (w), 1486 (s), 1472 (m), 1455 (m), 1389 (w), 1327 (m), 1287 (m), 1252 (s), 1185 (m), 1157 (w), 1117 (w), 1077 (w), 1036 (w), 1012 (m), 913 (w), 825 (m), 776 (m), 733 (m), 677 (w), 610 (w), 581 (w), 538 (w), 478 (w), 410 (w) cm^−1^.

Absorption (toluene, *c* = 0.05mM): λ_max_ = 346 nm (ε_λ_ = 18000 L mol^-1^ cm^-1^).

Emission (toluene, λ_exc_ = 370 nm): λ_em_ = 389 nm (FWHM = 41 nm).

Emission (solid state, λ_exc_ = 380 nm): λ_em_ = 447 nm (FWHM = 85 nm).

3-(4-butylphenyl)-6,6-dimethyl-6*H*-5λ^4^,6λ^4^-benzo[3,4][1,2]azaborolo[1,5-a]pyridine (BMe_2_‑PhC_4_)

Preparation according to GP3. No further purification of the crude product was necessary. **BMe_2_‑PhC_4_** was obtained as a colorless solid (51 mg, 156 µmol, 78 %).

^1^H NMR (700 MHz, CDCl_3_): δ = 0.09 (s, 6H, B(CH_3_)), 0.93–0.99 (m, 3H, CH_3_), 1.36–1.44 (m, 2H, CH_2_CH_3_), 1.62–1.69 (m, 2H, C*H*_2_CH_2_CH_3_), 2.67–2.73 (m, 2H, C*H*_2_CH_2_CH_2_CH_3_), 7.28–7.33 (m, 1H), 7.28–7.33 (m, 1H, 8-H), 7.33–7.37 (m, 2H, 14-H), 7.41–7.45 (m, 1H, 9-H), 7.53–7.58 (m, 2H, 13-H), 7.63–7.68 (m, 1H, 10-H), 7.83–7.87 (m, 1H, 7-H), 7.96–8.00 (m, 1H, 2-H), 8.12–8.16 (m, 1H, 3-H), 8.61 (d, *J* = 2.0 Hz, 1H, 5-H) ppm.

^13^C NMR (176 MHz, CDCl_3_): δ = 9.3 (B(CH_3_), 14.1 (CH_3_), 22.5, 33.7, 35.5 (CH_2_), 117.8 (C-2), 121.6 (C-7), 125.4 (C-14), 127.0 (C-13), 129.3 (C-8), 129.6 (C-10), 130.3 (C-9), 133.5 (C-15), 135.2 (C-4), 135.4 (C-6), 137.8 (C-3), 140.4 (C-5), 144.1 (C-12), 155.4 (C-1) ppm. The carbon atom C-11 next to the boron atom was not observed in the ^13^C NMR spectrum due to quadrupole relaxation.

^11^B NMR (128 MHz, CDCl_3_): δ = 1.2 ppm.

M.p. (DSC): 48 °C.

HRMS (EI): *m/z* for C_23_H_26_BN calc.: 327.2168 [M], found: 327.2158.

FTIR (ATR): $\tilde{\nu}$ = 3037 (w), 2955 (w), 2921 (s), 2884(w), 2857 (m), 2822 (w), 2197(w), 2155 (w), 1974 (w), 1912 (w), 1619 (m), 1557 (w), 1484 (vs), 1456 (w), 1446 (w), 1380 (w), 1326 (m), 1278 (m), 1230 (w), 1188 (w), 1172 (w),1155 (w), 1121 (w),1062 (w), 1030 (w),1018 (w), 1006 (w), 947 (w), 920 (w), 871 (w), 850 (w),822 (m), 779 (m), 761 (w), 739 (s), 599 (w), 549 (w), 470 (w), 444 (w) cm^-1^.

Absorption (toluene, *c* = 0.06 mM): λ_max_ = 336 nm (ε_λ_ = 18000 L mol^-1^ cm^-1^).

Emission (toluene, λ_exc_ = 360 nm): λ_em_ = 384 nm (FWHM = 38 nm).

Emission (solid state, λ_exc_ = 370 nm): λ_em_ = 414 nm (FWHM = 62 nm).

3-(4-(Dodecyloxy)phenyl)-6,6-dimethyl-6*H*-5λ^4^,6λ^4^-benzo[3,4][1,2]azaborolo[1,5-a]pyridine (BMe_2_-PhOC_12_)

Preparation according to GP3. The crude product was recrystallized from petroleum ether / EtOAc (10 / 1) and washed with cold petroleum ether (20 mL). **BMe_2_-PhOC_12_** was obtained as a colorless solid (51 mg, 112 µmol, 38 %).

^1^H NMR (700 MHz, CDCl_3_): δ = 0.08 (s, 6H B(CH_3_)), 0.86–0.91 (m, 3H, CH_3_), 1.12–1.49 (m, 16H, CH_2_), 1.79–1.86 (m, 2H, OCH_2_CH_2_C*H*_2_), 4.00–4.05 (m, 2H, OCH_2_C*H*_2_), 7.02–7.06 (m, 2H, 14-H), 7.27–7.32 (m, 1H, 8-H), 7.40–7.44 (m, 1H, 9-H), 7.54–7.57 (m, 2H, 13-H), 7.63–7.66 (m, 1H, 10-H), 7.82–7.86 (m, 1H, 7-H), 7.93–7.98 (m, 1H, 2-H), 8.08–8.12 (m, 1H, 3-H), 8.57 (d, *J* = 2.0 Hz, 1H, 5-H) ppm.

^13^C NMR (176 MHz, CDCl_3_): δ = 9.3 (BCH_3_), 14.3 (CH_3_), 22.8, 26.2, 29.4, 29.5, 29.5, 29.7, 29.8, 29.8, 29.8, 32.1 (CH_2_), 68.4 (OCH_2_), 115.5 (C-14), 117.8 (C-2), 121.5 (C-7), 125.4 (C-8), 128.2 (C-12), 128.3 (C-13), 129.3 (C-9), 130.2 (C-10), 135.1 (C-4), 135.2 (C-6), 137.4 (C-3), 140.1 (C-5), 155.0 (C-1), 160.0 (C-15) ppm. The carbon atom C-11 next to the boron atom was not observed in the ^13^C NMR spectrum due to quadrupole relaxation.

^11^B NMR (128 MHz, CDCl_3_): δ = 1.4 ppm.

M.p. (DSC): 79 °C.

FTIR (ATR): $\tilde{\nu}$ = 3039 (w), 2921 (vs), 2852 (s), 2823 (w), 1607 (s), 1579 (w), 1485 (vs), 1387 (w), 1328 (w), 1285 (s), 1252 (vs), 1181 (m), 1155 (w), 1116 (w), 1032 (m), 946 (w), 826 (s), 779 (w), 738 (s), 547 (w), 441 (w) cm^−1^.

HRMS (EI): *m/z* for C_31_H_42_BNO calc.: 455.3359 [M], found: 455.3360.

Absorption (toluene, *c* = 0.05 mM): λ_max_ = 342 nm (ε_λ_ = 20000 L mol^-1^ cm^-1^).

Emission (toluene, λ_exc_ = 365 nm): λ_em_ = 385 nm (FWHM = 40 nm).

Emission (solid state, λ_exc_ = 365 nm): λ_em_ = 416 nm (FWHM = 64 nm).

3-(4-(Octyloxy)phenyl)-6*H*-5λ^4^-benzo[3,4][1,2]azaborolo[1,5-a]pyridine (BH_2_-PhOC_8_)

Preparation according to GP3. The crude product was recrystallized from petroleum ether / EtOAc (10 / 1). **BH_2_-PhOC_8_** was obtained as a colorless solid (31 mg, 83 µmol, 26 %).

^1^H NMR (700 MHz, CDCl_3_): δ = 0.85–0.93 (m, 3H, CH_3_), 1.24–1.41 (m, 8H, CH_2_), 1.44–1.51 (m, 2H, OCH_2_CH_2_C*H*_2_), 1.79–1.85 (m, 2H, OCH_2_C*H*_2_), 3.21–3.79 (m, 2H, BH_2_), 4.00–4.07 (m, 2H, OCH_2_), 7.01–7.08 (m, 2H, 14-H), 7.31–7.35 (m, 1H, 8-H), 7.42–7.48 (m, 1H, 9-H), 7.50–7.60 (m, 2H, 13-H), 7.78–7.82 (m, 1H, 10-H), 7.85–7.89 (m, 1H, 7-H), 7.97–8.01 (m, 1H, 2-H), 8.10–8.14 (m, 1H, 3-H), 8.82–8.84 (m, 1H, 5-H) ppm.

^13^C NMR (176 MHz, CDCl_3_): δ = 14.3 (CH_3_), 22.8, 26.2, 29.4, 29.4, 29.5, 32.0 (CH_2_), 68.4 (OCH_2_), 115.5 (C-14), 118.0 (C-2), 121.5 (C-7), 125.4 (C-8), 127.9 (C-15), 128.1 (C-13), 130.2 (C-9), 130.4 (C-10), 134.6 (C-4), 136.8 (C-6), 137.4 (C-3), 141.8 (C-5), 156.6 (C-1), 160.1 (C-12) ppm. The carbon atom C-11 next to the boron atom was not observed in the ^13^C NMR spectrum due to quadrupole relaxation.

^11^B NMR (128 MHz, CDCl_3_): δ = -8.1 ppm.

M.p. (DSC): 86 °C

FTIR (ATR): $\tilde{\nu}$ = 3048 (w), 2925 (vs), 2856 (s), 2346 (vs), 2209 (w), 2185 (w), 2160 (w), 2119 (w), 2085 (w), 2054 (w), 2034 (w), 2021 (w), 1985 (w), 1952 (w), 1622 (m), 1607 (m), 1489 (s), 1469 (m), 1454 (m), 1387 (w), 1328 (m), 1289 (m), 1257 (s), 1185 (m), 1142 (w), 1036 (w), 1012 (m), 824 (vs), 775 (vs), 732 (vs), 677 (m), 539 (w), 477 (w), 424 (w), 412 (w) cm^−1^.

HRMS (EI): *m/z* for C_31_H_41_BNO^-^ calc.: 370.2342 [M-H]^-^, found: 370.2343.

Absorption (toluene, *c* = 0.05 mM): λ_max_ = 346 nm (ε_λ_ = 19000 L mol^-1^ cm^-1^).

Emission (toluene, λ_exc_ = 380 nm): λ_em_ = 391 nm (FWHM = 39 nm).

Emission (solid state, λ_exc_ = 370 nm): λ_em_ = 452 nm (FWHM = 86 nm).

3-(4-(Butyloxy)phenyl)-6*H*-5λ^4^-benzo[3,4][1,2]azaborolo[1,5-a]pyridine (BH_2_-PhOC_4_)

Preparation according to GP3. The crude product was recrystallized from petroleum ether / EtOAc (10 / 1). **BH_2_-PhOC_4_**was obtained as a colorless solid (52 mg, 165 µmol, 45 %).

^1^H NMR (700 MHz, CDCl_3_): δ = 0.97–1.03 (m, 3H, CH_3_), 1.48–1.55 (m, 2H, OCH_2_CH_2_C*H*_2_), 1.77–1.85 (m, 2H, OCH_2_C*H*_2_), 3.50 (d, *J* = 99.4 Hz, 2H, BH_2_), 4.00–4.05 (m, 2H, OCH_2_), 7.01–7.05 (m, 2H, 14-H), 7.31–7.35 (m, 1H, 8-H), 7.42–7.47 (m, 1H, 9-H), 7.53–7.57 (m, 2H, 13-H), 7.78–7.82 (m, 1H, 10-H), 7.85–7.89 (m, 1H, 7-H), 7.97–8.00 (m, 1H, 2-H), 8.10–8.14 (m, 1H, 3-H), 8.82–8.84 (m, 1H, 5-H) ppm.

^13^C NMR (176 MHz, CDCl_3_): δ = 14.0 (CH_3_), 19.4, 31.4 (CH_2_), 68.1 (OCH_2_), 115.5 (C-14), 118.0 (C-2), 121.5 (C-7), 125.4 (C-8), 127.9 (C-15), 128.1 (C-13), 130.2 (C-9), 130.4 (C-10), 134.6 (C-4), 136.8 (C-6), 137.4 (C-3), 141.8 (C-5), 156.6 (C-1), 160.1 (C-12) ppm. The carbon atom C-11 next to the boron atom was not observed in the ^13^C NMR spectrum due to quadrupole relaxation.

^11^B NMR (128 MHz, CDCl_3_): δ = -7.5 ppm.

FTIR (ATR): $\tilde{\nu}$ = 3045 (w), 2958 (s), 2935 (m), 2872 (m), 2348 (vs), 2036 (w), 1622 (s), 1607 (s), 1489 (vs), 1454 (m), 1385 (m), 1328 (m), 1289 (s), 1255 (vs), 1185 (m), 1144 (w), 1036 (m), 1012 (m), 971 (w), 826 (vs), 775 (vs), 732 (vs), 677 (m), 539 (w), 479 (w), 410 (w) cm^−1^.

HRMS (EI): *m/z* for C_21_H_21_BNO^-^ calc.:314.1716 [M-H]^-^, found: 314.1721.

M.p.: (DSC): 129 °C

Absorption (toluene, *c* = 0.04 mM): λ_max_ = 346 nm (ε_λ_ = 19000 L mol^-1^ cm^-1^).

Emission (toluene, λ_exc_ = 370 nm): λ_em_ = 386 nm (FWHM = 41 nm).

Emission (solid state, λ_exc_ = 390 nm): λ_em_ = 444 nm (FWHM = 77 nm).

3-(3,4-Bis(dodecyloxy)phenyl)-6,6-dimethyl-6*H*-5λ^4^,6λ^4^-benzo[3,4][1,2]azaborolo[1,5-a]pyridine (BMe_2_-Ph(OC_12_)_2_)

Preparation according to GP3. The crude product was recrystallized from petroleum ether / EtOAc (10 / 1). **BMe_2_-Ph(OC_12_)**_2_ was obtained as a colorless solid (31 mg, 48 µmol, 26 %).

^1^H NMR (700 MHz, CDCl_3_): δ = 0.09 (s, 6H, B(CH_3_)), 0.86–0.91 (m, 6H, CH_3_), 1.23–1.43 (m, 32H, CH_2_), 1.47–1.53 (m, 4H, OCH_2_CH_2_C*H*_2_), 1.84–1.90 (m, 4H, OCH_2_C*H*_2_), 4.04–4.12 (m, 4H, OCH_2_), 6.98–7.03 (m, 1H, 16-H), 7.09–7.12 (m, 1H, 13-H), 7.13–7.18 (m, 1H, 17-H), 7.27–7.32 (m, 1H, 8-H), 7.41–7.45 (m, 1H, 9-H), 7.61–7.67 (m, 1H, 10-H), 7.82–7.87 (m, 1H, 7-H), 7.92–7.98 (m, 1H, 2-H), 8.07–8.11 (m, 1H, 3-H), 8.53–8.58 (m, 1H, 5-H) ppm.

^13^C NMR (176 MHz, CDCl_3_): δ = 9.3 (BCH_3_), 14.3 (CH_3_), 22.8, 26.2, 26.2, 29.4, 29.5, 29.5, 29.6, 29.6, 29.8 ,29.8, 29.8, 29.9, 32.1 (CH_2_), 69.5, 69.9 (OCH_2_), 112.9 (C-13), 114.2 (C-16), 117.7 (C‑3), 120.0 (C-17), 121.5 (C-7), 125.4 (C-8), 128.9 (C-12), 129.3 (C-10), 130.3 (C-9), 135.2 (C‑4), 135.4 (C-6), 137.6 (C-3), 140.2 (C-5), 149.9 (C-14), 150.3 (C-15), 155.1 (C-1 ) ppm. The carbon atom next to the boron atom was not observed in the ^13^C NMR spectrum due to quadrupole relaxation.

^11^B NMR (128 MHz, CDCl_3_): δ = 2.3 ppm.

M.p. (DSC): 71 °C

FTIR (ATR): $\tilde{\nu}$ = 3048 (w), 2921 (vs), 2852 (s), 2056 (w), 1970 (w), 1620 (w), 1603 (w), 1585 (w), 1520 (m), 1489 (s), 1469 (m), 1379 (w), 1324 (m), 1271 (m), 1252 (s), 1214 (m), 1171 (w), 1142 (m), 1061 (w), 1028 (w), 949 (w), 842 (w), 806 (w), 779 (w), 738 (m), 638 (w), 528 (w), 496 (w), 437 (w) cm^−1^.

HRMS (EI): *m/z* for C_42_H_66_BNO_2_ calc.: 639.5187 [M], found: 639.5189.

Absorption (toluene, *c* = 0.03 mM): λ_max_ = 352 nm (ε_λ_ = 19000 L mol^-1^ cm^-1^).

Emission (toluene, λ_exc_ = 365 nm): λ_em_ = 392 nm (FWHM = 53 nm).

Emission (solid state, λ_exc_ = 370 nm): λ_em_ = 417 nm (FWHM = 60 nm).

6,6-Dimethyl-3-(3,4,5-tris(dodecyloxy)phenyl)-6*H*-5λ^4^,6λ^4^-benzo[3,4][1,2]azaborolo[1,5-a]pyridine (BMe_2_-Ph(OC_12_)_3_)

Preparation according to GP3. The crude product was recrystallized from petroleum ether. **BMe_2_‑Ph(OC_12_)_3_** was obtained as a colorless solid (114 mg, 138 µmol, 57 %).

^1^H NMR (500 MHz, CDCl_3_): δ = 0.09 (s, 7H, B(CH_3_)), 0.85–0.92 (m, 9H, CH_3_), 1.21–1.43 (m, 48H, CH_2_), 1.45–1.55 (m, 6H, OCH_2_CH_2_C*H*_2_), 1.74–1.82 (m, 2H, C-15(OCH_2_C*H*_2_)), 1.82–1.90 (m, 4H, C-14(OCH_2_C*H*_2_)), 4.00–4.04 (m, 2H, C-15(OCH_2_)), 4.05–4.10 (m, 4H, C-14(OCH_2_)), 6.74 (s, 2H, 13-H),7.28–7.33 (m, 1H, 8-H), 7.40–7.47 (m, 1H, 9-H), 7.60–7.68 (m, 1H, 10-H), 7.83–7.87 (m, 1H, 7-H), 7.94–7.98 (m, 1H, 2-H), 8.06–8.11 (m, 1H, 3-H), 8.51–8.56 (m, 1H, 5-H) ppm.

^13^C NMR (126 MHz, CDCl_3_) δ = 8.1 (B(CH_3_), 14.1 (CH_3_), 22.7, 26.1, 26.2, 29.4, 29.4, 29.5, 29.6, 29.7, 29.7, 29.7, 29.8, 29.8, 30.4, 32.0, 32.0 (CH_2_), 69.5, 73.7 (OCH_2_), 105.9 (C-13), 117.6 (C-2), 121.5 (C-7), 125.3 (C-8), 129.2 (C-10), 130.2 (C-9), 131.4 (C-12), 135.0 (C-6), 135.6 (C-4), 137.9 (C-3), 139.2 (C-, 140.3 (C-5), 153.9 (C-14), 155.4 (C-1) ppm. The carbon atom next to the boron atom was not observed in the ^13^C NMR spectrum due to quadrupole relaxation.

^11^B NMR (160 MHz, CDCl_3_): δ = 1.3 ppm.

M.p. (DSC): 46 °C

FTIR (ATR): $\tilde{\nu}$ = 2921 (vs), 2852 (s), 2134 (w), 1620 (w), 1583 (m), 1485 (s), 1467 (s), 1432 (m), 1377 (m), 1346 (m), 1326 (w), 1283 (w), 1250 (m), 1173 (w), 1155 (w), 1116 (s), 1061 (w), 1030 (w), 1006 (w), 949 (w), 824 (w), 779 (w), 761 (w), 738 (m), 649 (w), 565 (w), 528 (w), 496 (w), 430 (w) cm^−1^.

HRMS (EI): *m/z* for C_55_H_90_BNO_3_^+^ calc.: 824.7087 [M+H]^+^, found: 824.7090.

Absorption (toluene, *c* = 0.03 mM): λ_max_ = 342 nm (ε_λ_ = 20000 L mol^-1^ cm^-1^).

Emission (toluene, λ_exc_ = 380 nm): λ_em_ = 410 nm (FWHM = 68 nm).

Emission (solid state, λ_exc_ = 365 nm): λ_em_ = 431 nm (FWHM =71 nm).

3-(3,4-Bis(dodecyloxy)phenyl)-6*H*-5λ^4^-benzo[3,4][1,2]azaborolo[1,5-a]pyridine (BH_2_-Ph(OC_12_)_2_)

Preparation according to GP3. The crude product was recrystallized from petroleum ether. **BH_2_-Ph(OC_12_)_2_** was obtained as a: pale yellow solid (52 mg, 85 µmol, 31 %).

^1^H NMR (500 MHz, CDCl_3_): δ = 0.85–0.91 (m, 6H, CH_3_), 1.18–1.41 (m, 32H, CH_2_), 1.45–1.55 (m, 4H, OCH_2_CH_2_C*H*_2_), 1.77–1.92 (m, 4H, OCH_2_C*H*_2_), 3.19–3.83 (m, 2H, BH_2_), 4.03–4.12 (m, 4H, OCH_2_), 6.97–7.02 (m, 1H, 16-H), 7.09–7.13 (m, 1H, 13-H), 7.13–7.18 (m, 1H, 17-H), 7.30–7.36 (m, 1H, 8-H), 7.41–7.48 (m, 1H, 9-H), 7.78–7.83 (m, 1H, 10-H), 7.85–7.89 (m, 1H, 7-H), 7.95–8.01 (m, 1H, 2-H), 8.09–8.14 (m, 1H, 3-H), 8.80–8.84 (m, 1H, 5-H) ppm.

^13^C NMR (126 MHz, CDCl_3_): δ = 14.3 (CH_3_), 22.8, 26.2, 29.4, 29.5, 29.6, 29.8, 29.8, 29.9, 32.1 (CH_2_), 69.4, 69.8 (OCH_2_), 112.5 (C-13), 114.2 (C-16) 117.9 (C-2), 119.8 (C-17), 121.5 (C-7), 125.4 (C-8), 128.5 (C-12), 130.2 (C-9), 130.4 (C-10), 134.8 (C-4), 136.8 (C-6), 137.5 (C-3), 141.9 (C-5), 150.0 (C-14), 150.3 (C-15), 156.6 (C-1) ppm. The carbon atom next to the boron atom was not observed in the ^13^C NMR spectrum due to quadrupole relaxation.

^11^B NMR (160 MHz, CDCl_3_): δ = -3.33 ppm.

FTIR (ATR): $\tilde{\nu}$ = 2923 (vs), 2852 (s), 2346 (w), 2160 (w), 2101 (w), 1724 (w), 1622 (w), 1601 (w), 1522 (w), 1491 (m), 1467 (m), 1456 (m), 1379 (w), 1324 (w), 1257 (m), 1214 (m), 1144 (m), 1040 (w), 1014 (w), 840 (w), 806 (w), 775 (m), 732 (m), 677 (w), 545 (w), 441 (w) cm^−1^.

HRMS (EI): *m/z* for C_41_H_61_BNO_2_^-^ calc.: 610.4795 [M-H]^-^, found: 610.4800.

M.p. (DSC): 61 °C

Absorption (toluene, *c* = 0.03 mM): λ_max_ = 346 nm (ε_λ_ = 16000 L mol^-1^ cm^-1^).

Emission (toluene, λ_exc_ = 380 nm): λ_em_ = 395 nm (FWHM = 56 nm).

Emission (solid state, λ_exc_ = 370 nm): λ_em_ = 462 nm (FWHM = 101 nm).

6,6-Dimethyl-3-(4-(3-((2,2,3,3,4,4,5,5,6,6,7,7,8,8,8-pentadecafluorooctyl)oxy)propyl)phenyl)-6*H*-5λ^4^,6λ^4^-benzo[3,4][1,2]azaborolo[1,5-a]pyridine (BMe_2_-PhC_3_OCC_7_^F^)

Preparation according to GP3. The crude product was recrystallized from petroleum ether / EtOAc (10 / 1). **BMe_2_-PhC_3_OCC_7_^F^** was obtained as a colorless solid (63 mg, 89 µmol, 69 %).

^1^H NMR (400 MHz, CDCl_3_): δ = 0.09 (s, 6H, B(CH_3_)), 1.93–2.04 (m, 2H, 17-H), 2.76–2.84 (m, 2H, 16-H), 3.60–3.68 (m, 2H, 18-H), 3.90–4.01 (m, 2H, 19-H), 7.27–7.33 (m, 1H, 8-H), 7.33–7.38 (m, 2H, 14-H), 7.39–7.50 (m, 1H, 9-H), 7.53–7.60 (m, 2H, 13-H), 7.62–7.72 (m, 1H, 10-H), 7.82–7.88 (m, 1H, 7-H), 7.95–8.02 (m, 1H, 2-H), 8.10–8.18 (m, 1H, 3-H), 8.58–8.63 (m, 1H, 5-H) ppm.

^13^C-NMR (176 MHz, CDCl_3_) δ = 29.7 (CH_3_), 31.0 (C‑17), 31.6 (C‑16), 67.9 (C-19), 72.0 (C-18), 117.7 (C‑2), 121.5 (C‑9), 125.3 (C‑7), 127.0 (C‑13), 129.2 (C‑8), 129.6 (C‑14), 130.3 (C‑4), 133.8 (C‑12), 135.0 (C‑3), 135.1 (C‑10), 137.7 (C‑15), 140.3 (C‑6), 142.5 (C‑5), 155.4 (C‑1) ppm. The carbon atom next to the boron atom was not observed in the ^13^C NMR spectrum due to quadrupole relaxation.

^11^B NMR (128 MHz, CDCl_3_): δ = 0.64 ppm.

^19^F-NMR (376 MHz, CDCl_3_): δ = -126.04, -122.67, -121.96, -119.51– -119.42 (m), -80.71 (t, *J* = 10.0 Hz) ppm.

FTIR (ATR): $\tilde{\nu}$ = 2919 (m), 2852 (w), 2823 (w), 2152 (w), 1991 (w), 1738 (w), 1620 (w), 1485 (m), 1456 (w), 1367 (w), 1326 (w), 1238 (vs), 1204 (vs), 1146 (vs), 1028 (m), 959 (w), 910 (w), 883 (w), 838 (m), 808 (m), 779 (m), 738 (s), 710 (m), 702 (m), 659 (m), 547 (m), 469 (w), 443 (w) cm^−1^.

HRMS (EI): *m/z* for C_30_H_26_BNO_2_^+^ calc.: 712.1862 [M+H]^+^, found: 712.1862.

M.p. (DSC): 58 °C

Absorption (toluene, *c* = 0.02 mM): λ_max_ = 338nm (ε_λ_ = 10000 L mol^-1^ cm^-1^).

Emission (toluene, λ_exc_ = 3650nm): λ_em_ = 384 nm (FWHM = 39 nm).

Emission (solid state, λ_exc_ = 370 nm): λ_em_ = 394 nm (FWHM = 68 nm).

8-(4-(Dodecyloxy)phenyl)-6*H*-5λ^4^-benzo[3,4][1,2]azaborolo[1,5-a]pyridine (C_12_OPh-BH_2_)

Preparation according to GP3. The crude product was recrystallized from petroleum ether / EtOAc (10 / 1). **C_12_OPh-BH_2_** was obtained as a colorless solid (7 mg, 16 µmol, 7 %).

^1^H NMR (700 MHz, CDCl_3_): δ = 0.89–0.93 (m, 3H, CH_3_), 1.25–1.43 (m, 16H, CH_2_), 1.47–1.52 (m, 2H, OCH_2_CH_2_C*H*_2_), 1.80–1.87 (m, 2H, OCH_2_C*H*_2_), 3.28–3.72 (m, 2H, BH_2_), 4.01–4.06 (m, 2H, OCH_2_), 6.99–7.03 (m, 2H, 14-H), 7.31–7.35 (m, 1H, 4-H), 7.53–7.57 (m, 1H, 8-H), 7.64–7.68 (m, 2H, 13-H), 7.91–7.95 (m, 1H, 7-H), 7.97–8.03 (m, 3H, 2-H, 3-H, 10-H), 8.65–8.68 (m, 1H, 5-H) ppm.

^13^C NMR (176 MHz, CDCl_3_) δ = 14.3 (CH_3_), 22.8, 26.2, 29.5, 29.5, 29.6, 29.7, 29.8, 29.8, 29.8, 32.1 (CH_2_), 68.2 (OCH_2_), 114.9 (C-14), 118.1 (C-2), 120.7 (C-4), 122.0 (C-7), 124.4 (C-8), 128.5 (C-10), 128.6 (C-13), 134.1 (C-12), 135.5 (C-6), 139.3 (C-3), 142.8 (C-8), 144.2 (C-5), 158.6 (C‑1)f, 159.0 (C-15) ppm. The carbon atom next to the boron atom was not observed in the ^13^C NMR spectrum due to quadrupole relaxation.

^11^B NMR (128 MHz, CDCl_3_) δ = -7.68 ppm.

M.p. (DSC): 92 °C

FTIR (ATR): $\tilde{\nu}$ = 2952 (w), 2920 (vs), 2852 (m), 2332 (w), 2280 (w), 1620 (m), 1600 (m), 1513 (m), 1478 (m), 1465 (w), 1439 (w), 1390 (w), 1332 (w), 1302 (w), 1277 (w), 1247 (m), 1181 (m), 1159 (w), 1113 (w), 1080 (w), 1028 (w), 1006 (w), 965 (w), 842 (w), 825 (w), 810 (w), 771 (s), 745 (m), 723 (w), 683 (w), 614 (w), 539 (w), 482 (w) cm^-1^.

HRMS (ESI): *m/z* für C_29_H_37_BNO^-^ calc.: 426.2968 [M-H]^-^, found: 426.2944.

Absorption (toluene, *c* = 0.04 mM): λ_max_ = 346 nm (ε_λ_ = 21000 L mol^-1^ cm^-1^).

Emission (toluene, λ_exc_ = 360 nm): λ_em_ = 393 nm (FWHM = 54 nm).

Emission (solid state, λ_exc_ = 370 nm): λ_em_ = 473 nm (FWHM = 107 nm).

1. Mesomorphic properties
   1. POM textures


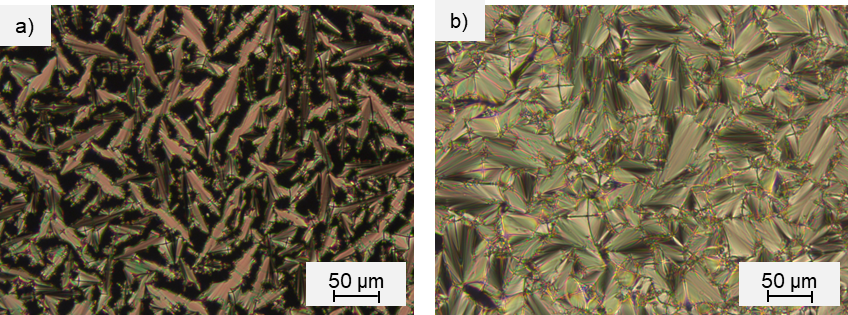


Figure S1: Textures obtained on the POM upon cooling a) BH_2_-PhC_4_ (64 °C, cooling rate 5 K / min), b) BH_2_-PhC_12_ (100 °C, cooling rate 5 K / min).


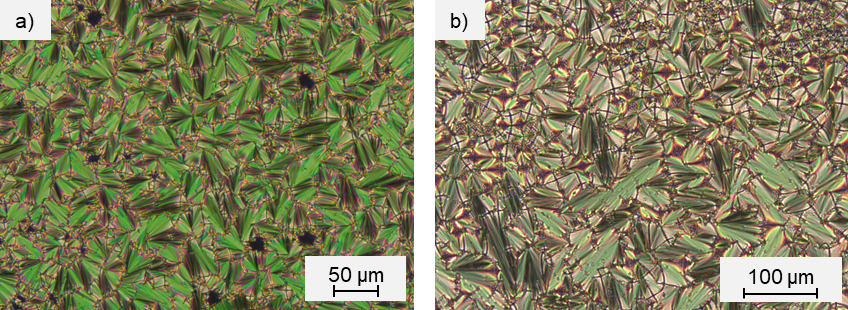


Figure S2: Textures obtained on the POM upon cooling a) BH_2_-PhOC_12_ (148 °C, cooling rate 5 K / min), b) BH_2_-PhOC_8_ (135 °C, cooling rate 5 K / min).


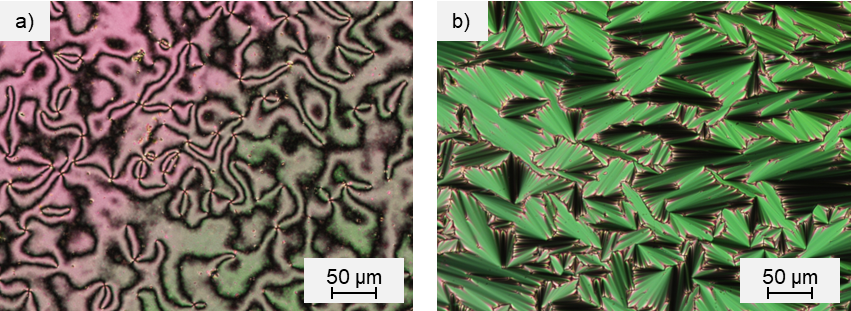


Figure S3: Textures obtained on the POM upon cooling a) BH_2_-PhOC_4_ (130 °C, cooling rate 10 K / min), b) BH_2_-PhOC_4_ (120 °C, cooling rate 10 K / min).


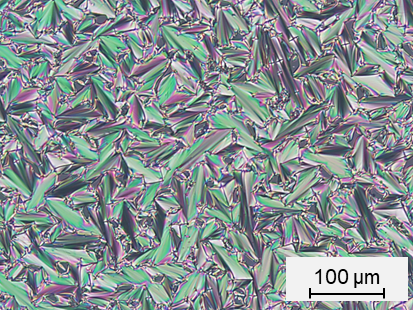


Figure S4: Texture obtained on the POM upon cooling BH_2_-Ph(OC_12_)_2_ (56 °C, cooling rate 5 K / min).


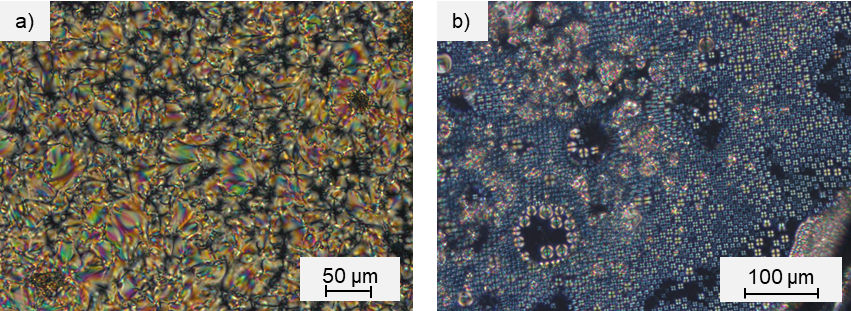


Figure S5: Textures obtained on the POM upon cooling a) BMe_2_-PhC_3_OCC_7_^F^ (50 °C, cooling rate 2 K / min), b) C_12_OPh-BH_2_ (106 °C, cooling rate 5 K / min).

- 1. DSC

Figure S6: DSC thermograms (heating / cooling rate 10 K / min) of a) BH_2_-PhC_4_, b) BH_2_-PhC_12_.

Figure S7: DSC thermograms (heating / cooling rate 10 K / min) of a) BH_2_-PhOC_12_, b) BH_2_-PhOC_8_.

Figure S8: DSC thermograms (heating / cooling rate 10 K / min) of a) BH_2_-PhOC_4_, b) BH_2_-Ph(OC_12_)_2_.

Figure S9: DSC thermograms (heating / cooling rate 10 K / min) of a) C_12_O-BH_2_, b) BMe_2_-PhC_3_OCC_7_^F^.

Figure S10: DSC thermograms (heating/ cooling rate 10 K / min) of a) BMe_2_-PhC_4_, b) BMe_2_-PhOC_12_.

Figure S11: DSC thermograms (heating / cooling rate 10 K / min) of a) BMe_2_-Ph(OC_12_)_2_, b) BMe_2_-Ph(OC_12_)_3_.

Table S2: Phase transition temperatures with respective enthalpies Δ*H* of compounds BH_2_-Ar, BMe_2_-Ar, Ar-BH_2_.

| Substance | Phase transition / °C (Δ*H /*kJ/mol) | | Cycle | |  |
| --- | --- | --- | --- | --- | --- |
| **BH_2_-PhC_4_** | [SmA 30 (0.5)] Cr 76 (–9.0) I  I 71 (0.3) SmA ^[a], [b]^ | | 2^nd^ heating  2^nd^ cooling | |  |
| **BH_2_-PhC_12_** | [SmA 27 (6.0)] Cr_1_ 65 (-12.9) Cr_2_ 70 (-1.7) SmA 110 I ^[c]^  I 107 (0.9) SmA ^[b]^ | | 2^nd^ heating  2^nd^ cooling | |  |
| **BH_2_-PhOC_4_** | Cr 129 (-25.2) I  I 129 (0.6) N 120 (0.8) SmA 78 (15.5) Cr ^[a])^ | | 2^nd^ heating  2^nd^ cooling | |  |
| **BH_2_-PhOC_8_** | [SmA 40 (1.7)] Cr 86 (-5.7) SmA 143 (-3.2) I ^[b]^  I 147 (3.4) SmA | | 2^nd^ heating  2^nd^ cooling | |  |
| **BH_2_-PhOC_12_** | [SmA 29 (16.9)] Cr 79 (-30.4) SmA 144 (-4.6) I  I 148 (5.4) SmA ^b)^ | | 2^nd^ heating  2^nd^ cooling | |  |
| **BH_2_-Ph(OC_12_)_2_** | Cr 61 I  I 62 (2.6) SmA 19 (13.4) Cr ^[a]^ | | 2^nd^ heating  2^nd^ cooling | |  |
| **BMe_2_-PhC_4_** | Cr_1_ 48 (-1.1) Cr_2_ 83 (-14.4) I ^[d]^ | | 1^st^ heating | |  |
| **BMe_2_-PhOC_12_** | Cr_1_ 18 (29.7) Cr_2_ 79 (-42.9) I  I 27 (0.5) Cr_1_ | | 2^nd^ heating  2^nd^ cooling | |  |
| **BMe_2_-Ph(OC_12_)_2_** | Cr_1_ 6.5 Cr_2_ 40 (3.0) Cr_3_ 70 (-52.9) I  I 6 (4.8) Cr_1_ | | 2^nd^ heating  2^nd^ cooling | |  |
| **BMe_2_-Ph(OC_12_)_3_** | [I 21 (33.8)] Cr 46 (-52.9) I  I ^[b]^ | | 2^nd^ heating  2^nd^ cooling | |  |
| **C_12_OPh-BH_2_** | Cr 92 (-4.1) SmA 119 (-14.7) I  I 104 (14.5) SmA 91 (2.5) Cr | | 2^nd^ heating  2^nd^ cooling | |  |
| **BMe_2_-PhC_3_OCC_7_^F^** | | G -16 Sm 58 (-0.94) I  I 61 (0.69) Sm -10 G | | 2^nd^ heating  2^nd^ cooling | |

[a] monotropic mesophase, [b] cold crystallization in the next heating cycle, [c] clearing point peak too small for determination of Δ*H*, temperature taken from the POM measurement*,* [d] decomposition during the first heating cycle. Square brackets mark phases that show before cold crystallization in the heating.

- 1. SAXS and WAXS

Figure S12: Diffractograms of BH_2_-PhC_4_ with inserted 2D patterns, a) SAXS, measured at 55 °C, b) WAXS, measured at 65 °C.

Figure S13: Diffractograms of BH_2_-PhC_12_ with inserted 2D patterns, a) SAXS, measured at 98 °C, b) WAXS, measured at 100 °C.

Figure S14: Diffractograms of BH_2_-PhOC_12_ with inserted 2D patterns, a) SAXS, measured at 69 °C, b) WAXS, measured at 72 °C.

Figure S15: Diffractograms of BH_2_-PhOC_8_ with inserted 2D patterns, a) SAXS, measured at 135 °C, b) WAXS, measured at 120 °C.

Figure S16: WAXS Diffractograms of BH_2_-PhOC_4_ with inserted 2D patterns, a) Nematic phase, measured at 125 °C, b) SmA phase, measured at 95 °C.

Figure S17: SAXS Diffractograms of BH_2_-PhOC_4_ with inserted 2D patterns, a) Nematic phase, measured at 127 °C, b) SmA Phase, measured at 105 °C.

Figure S18: Diffractograms of BH_2_-Ph(OC_12_)_2_ with inserted 2D patterns, a) SAXS, measured at 46 °C, b) WAXS, measured at 46 °C.

Figure S19: Diffractograms of BMe_2_-PhC_3_OCC_7_^F^ with inserted 2D patterns, a) SAXS, measured at 41 °C, b) WAXS, measured at 45 °C.

Figure S20: Diffractograms of C_12_OPh-BH_2_ with inserted 2D patterns, a) SAXS, measured at 95 °C, b) WAXS, measured at 90 °C.

Figure S21: Mesophase widths and phases of calamitic boron *C,N*-chelates [a] monotropic mesophase, [b] crystallization determined from heating cycle because of cold crystallization.

Table S3: Detailed XRD data of BH_2_-PhC_4_- C_12_O-BH_2_.

| Substance | Mesophase | *q* / nm^-1^ | (*hkl*) | *d*_exp_ / Å |
| --- | --- | --- | --- | --- |
| **BH_2_-PhC_4_** | SmA at 55 °C  SmA at 65 °C | 3.45  16.76 | (001)  halo | 18.21  3.75 |
| **BH_2_-PhC_12_** | SmA at 98 °C  SmA at 100 °C | 2.32  13.97 | (001)  halo | 27.08  4.50 |
| **BH_2_-PhOC_12_** | SmA at 69 °C  SmA at 72 °C | 2.30  4.18  13.92 | (001)  (002)  halo | 27.32  15.03  13.92 |
| **BH_2_-PhOC_8_** | SmA at 135 °C  SmA at 120 °C | 2.58  15.73 | (001)  halo | 24.35  3.99 |
| **BH_2_-PhOC_4_** | N at 125 °C  SmA at 105 °C  SmA at 95 °C | 4.06  15.49  3.51  7.83  15.90 | (diffuse reflex)  (halo)  (001)  (002)  (halo) | 15.47  4.06  17.90  8.03  3.95 |
| **BH_2_-Ph(OC_12_)_2_** | SmA at 46 °C  SmA at 46 °C | 0.94  1.87  2.79  4.71  7.15  14.61 | (001)  (002)  (003)  (005)  (008)  halo | 66.84  33.60  22.52  13.35  8.79  4.30 |
| **BMe_2_-PhC_3_OCC_7_^F^** | SmA at 41 °C  SmA at 45 °C | 1.20  12.44 | (001)  (halo) | 52.36  5.05 |
| **C_12_OPh-BH_2_** | SmA at 95 °C  SmA at 90 °C | 1.56  3.12  4.68  6.24  7.86  9.44  13.30 | (001)  (002)  (003)  (004)  (005)  (006)  halo | 40.28  20.14  13.43  10.07  7.99  6.66  4.72 |

1. Computational Details

All structures relevant to the reaction mechanism were first built manually and explored in their conformational space using metadynamic simulations, based on tight-binding quantum chemical calculations as implemented in CREST^[61,62]^. All minimum structures and transition states were pre-optimized using GFN2-xTB^[63]^ with the Turbomole V7.8.1 program package^[64,65]^ and subsequently re-optimized at the DFT level using the MO6-2x^[66]^ functional and the def2-TZVP basis set^[45,46]^. The HOMO and LUMO were displayed using the IBOview^[67]^ software.

An additional xyz-file has been provided, which contains cartesian coordinates for all calculated species.

1. Photophysical properties
   1. Absorption and Emission

Figure S22: Absorption and emission spectra of a) BH_2_-PhC_4_ (toluene: *c* = 0.05 mM, λ_exc_ = 370 nm, solid state: λ_exc_ = 370 nm), b) BH_2_-PhC_12_ (toluene: *c* = 0.04 mM, λ_exc_ = 360 nm, solid state: λ_exc_ = 370 nm).

Figure S23: Absorption and emission spectra of a) BMe_2_-PhC_4_ (toluene: *c* = 0.06 mM, λ_exc_ = 360 nm, solid state: λ_exc_ = 370 nm), b) BH_2_-OC_12_ (toluene: *c* = 0.05 mM, λ_exc_ = 370 nm, solid state: λ_exc_ = 380 nm).

Figure S24: Absorption and emission spectra of a) BMe_2_-PhOC_12_ (toluene: *c* = 0.05 mM, λ_exc_ = 365 nm, solid state: λ_exc_ = 365 nm), b) BH_2_-PhOC_8_ (toluene: *c* = 0.05 mM, λ_exc_ = 380 nm, solid state: λ_exc_ = 370 nm).

**Figure S25:** Absorption and emission spectra of a) **BH_2_-PhOC_4_** (toluene: *c* = 0.04 mM, λ_exc_ = 370 nm, solid state: λ_exc_ = 390 nm), b) **BMe_2_-Ph(OC_12_)_2_** (toluene: *c* = 0.03 mM, λ_exc_ = 365 nm, solid state: λ_exc_ = 370 nm).

Figure S26: Absorption and emission spectra of a) BMe_2_-Ph(OC_12_)_3_ (toluene: *c* = 0.03 mM, λ_exc_ = 380 nm, solid state: λ_exc_ = 365 nm), b) BH_2_-Ph(OC_12_)_2_ (toluene: *c* = 0.03 mM, λ_exc_ = 380 nm, solid state: λ_exc_ = 370 nm).

Figure S27: Absorption and emission spectra of a) BMe_2_-PhC_3_OCC_7_^F^ (toluene: *c* = 0.02 mM, λ_exc_ = 360 nm, solid state: λ_exc_ = 370 nm), b) C_12_OPh-BH_2_ (toluene: *c* = 0.04 mM, λ_exc_ = 360 nm, solid state: λ_exc_ = 370 nm).


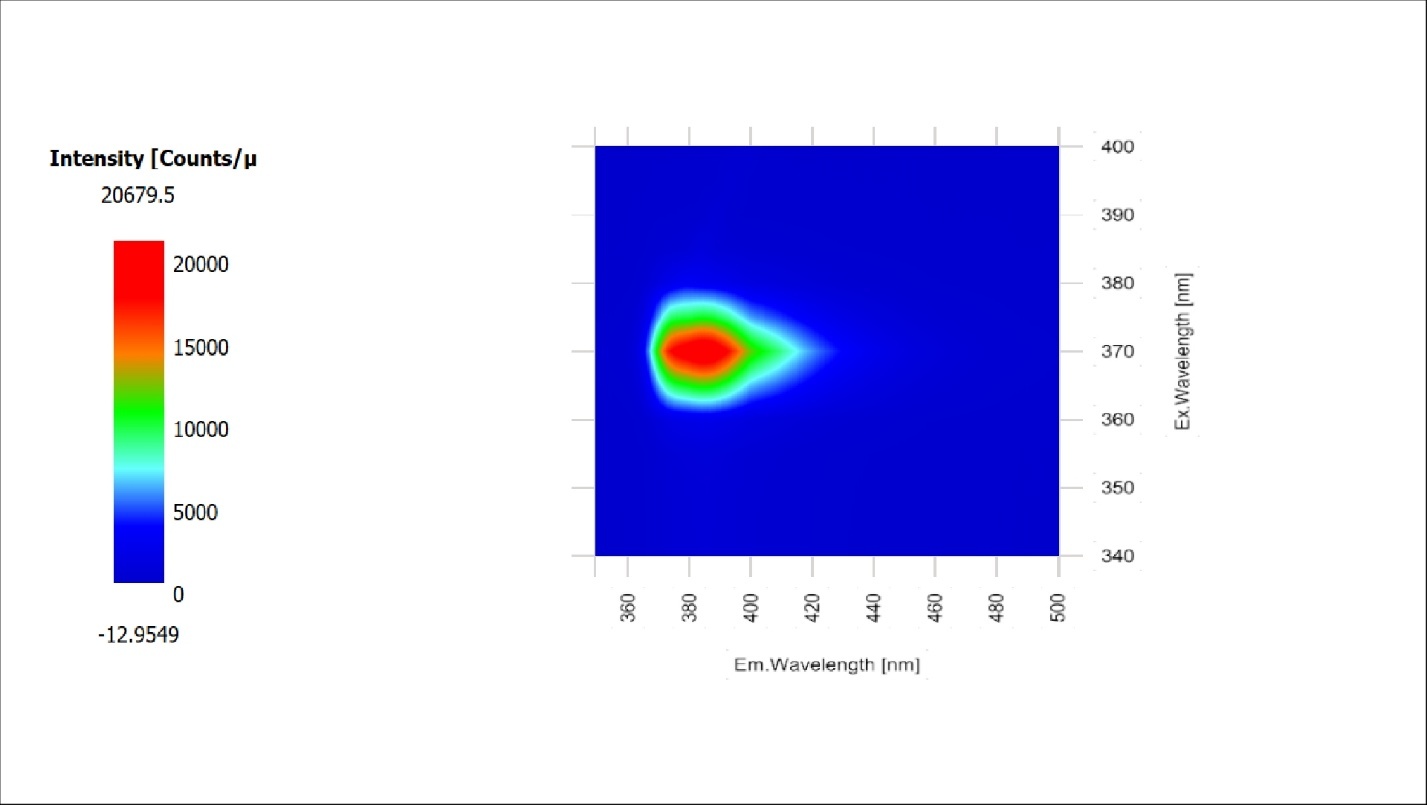


Figure S28: Excitation-emission map of BH_2_-PhC_4_ (toluene).


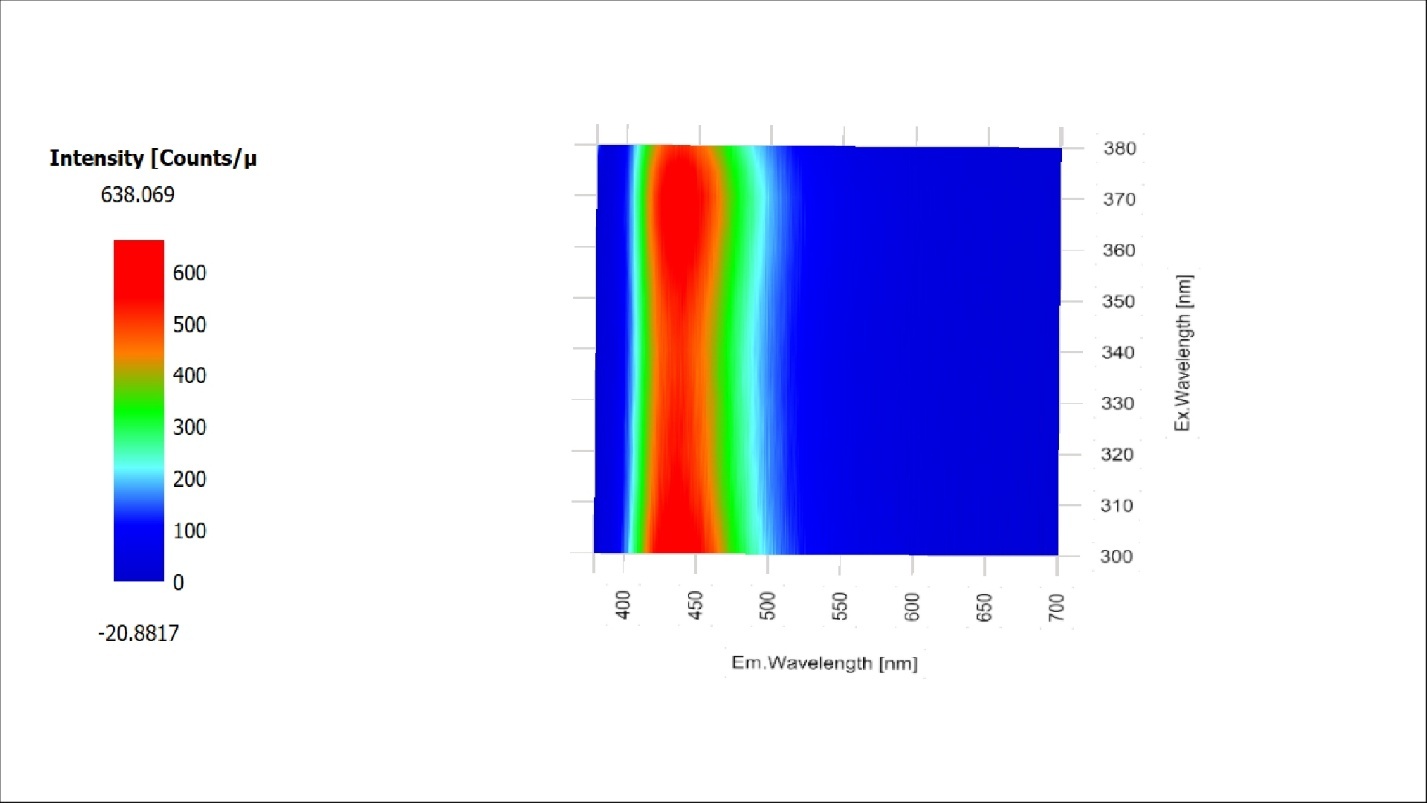


Figure S29: Excitation-emission map of BH_2_-PhC_4_ (solid).


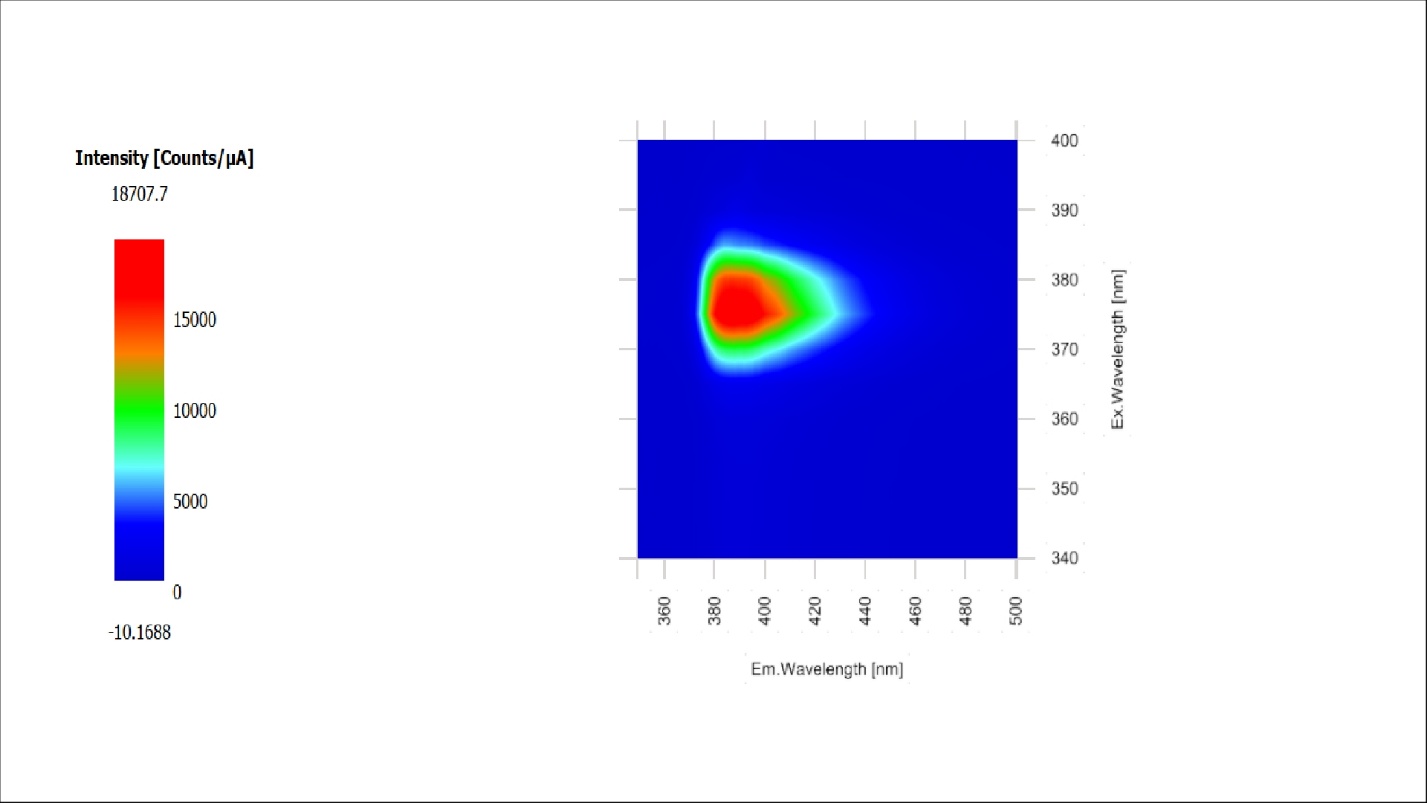


Figure S30: Excitation-emission map of BH_2_-PhOC_12_ (toluene).


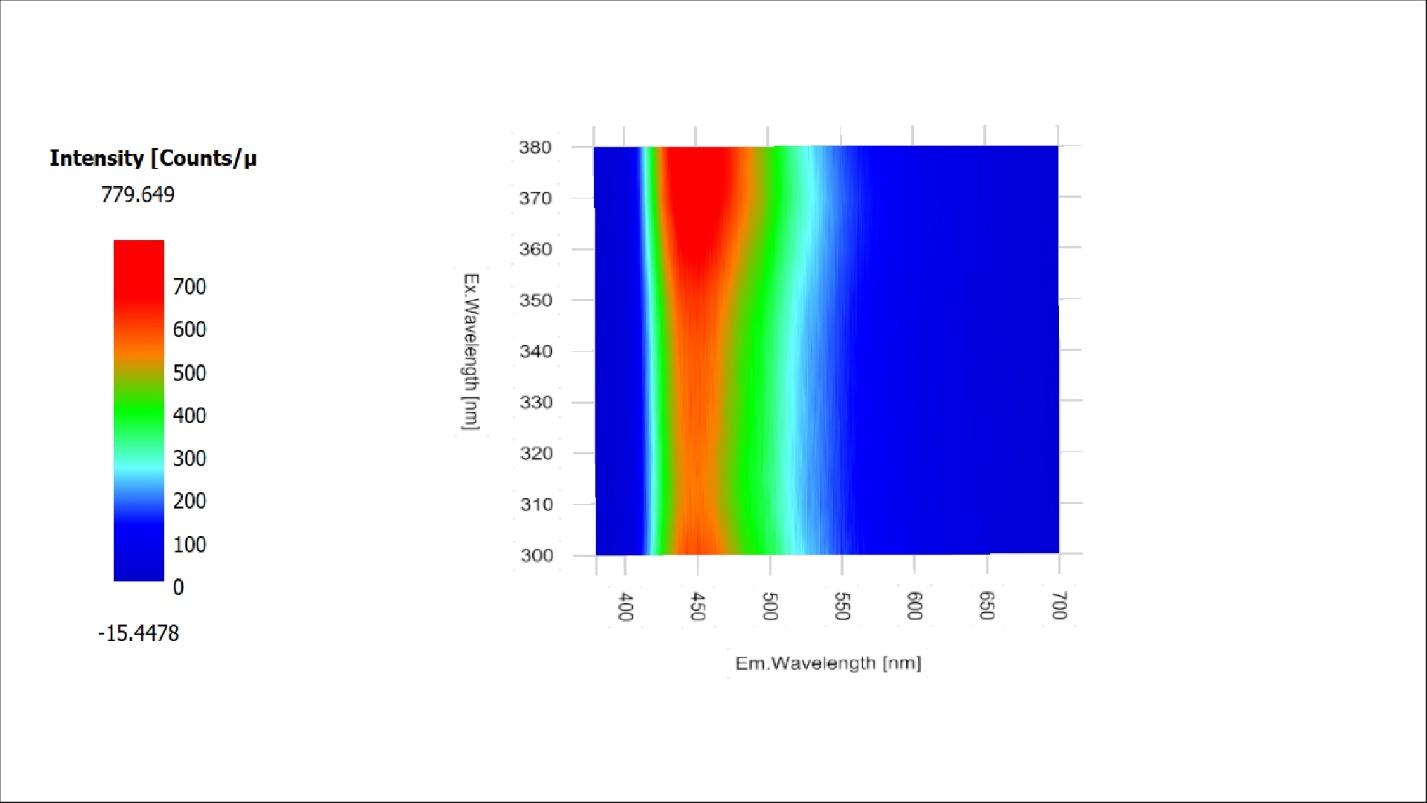


Figure S31: Excitation-emission map of BH_2_-PhOC_12_ (solid).


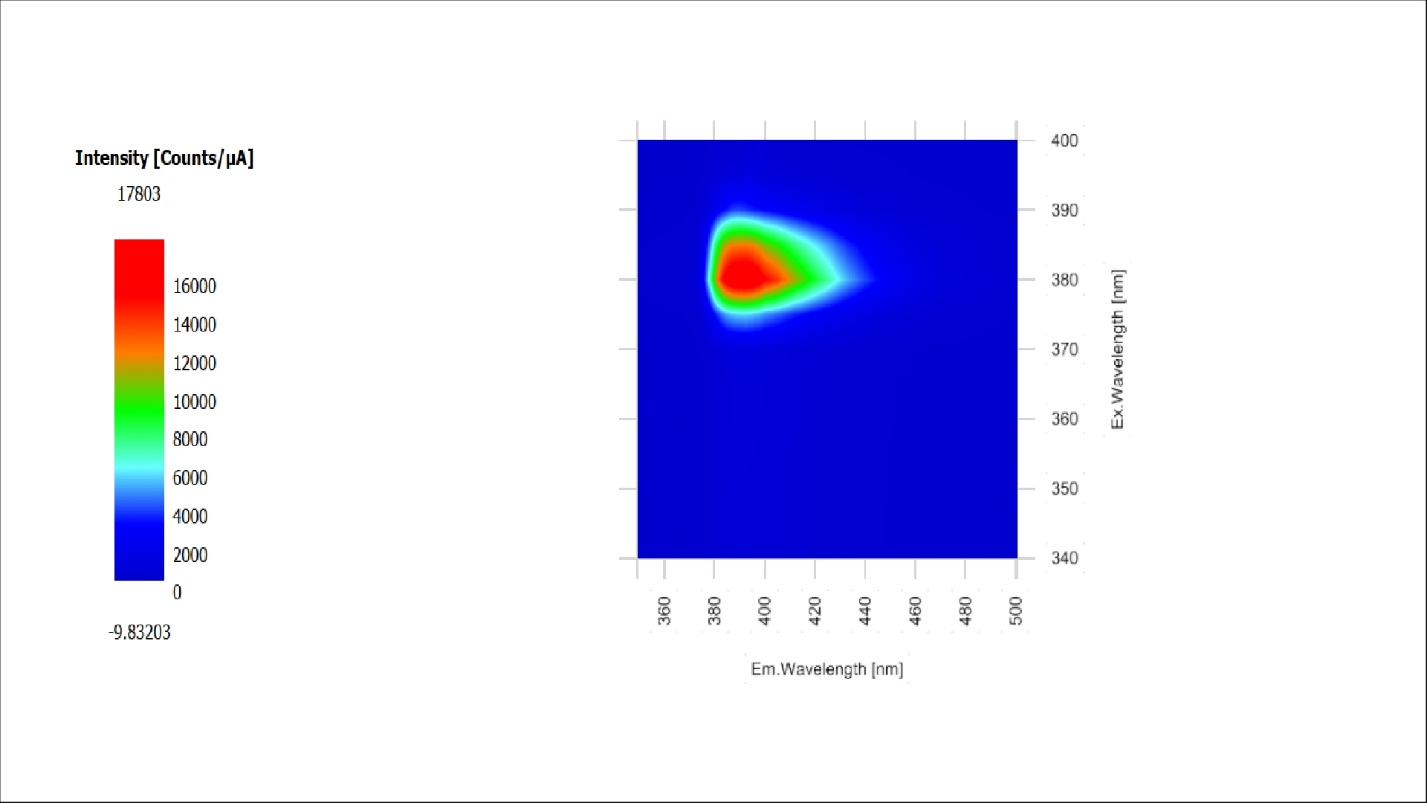


Figure S32: Excitation-emission map of BH_2_-PhOC_8_ (toluene).


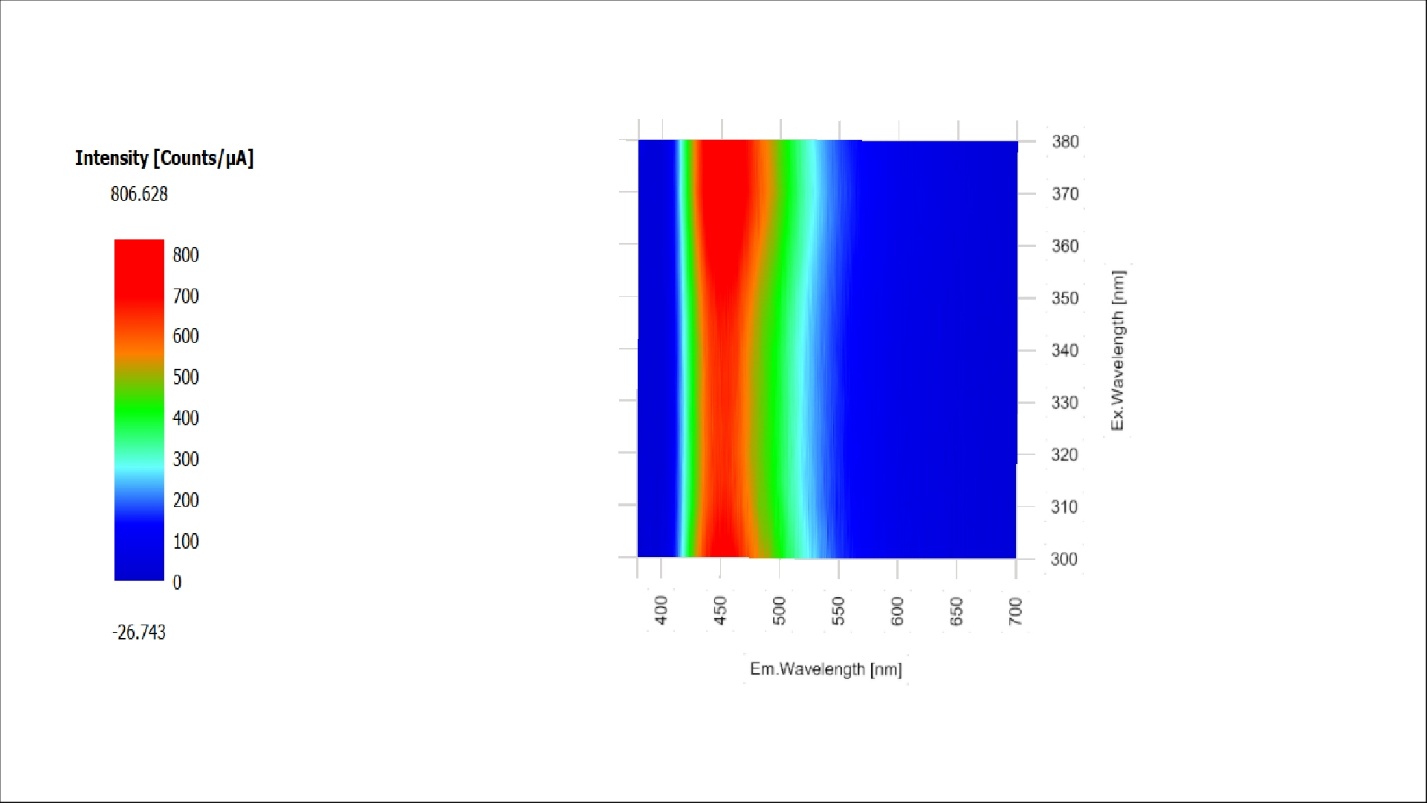


Figure S33: Excitation-emission map of BH_2_-PhOC_8_ (solid).


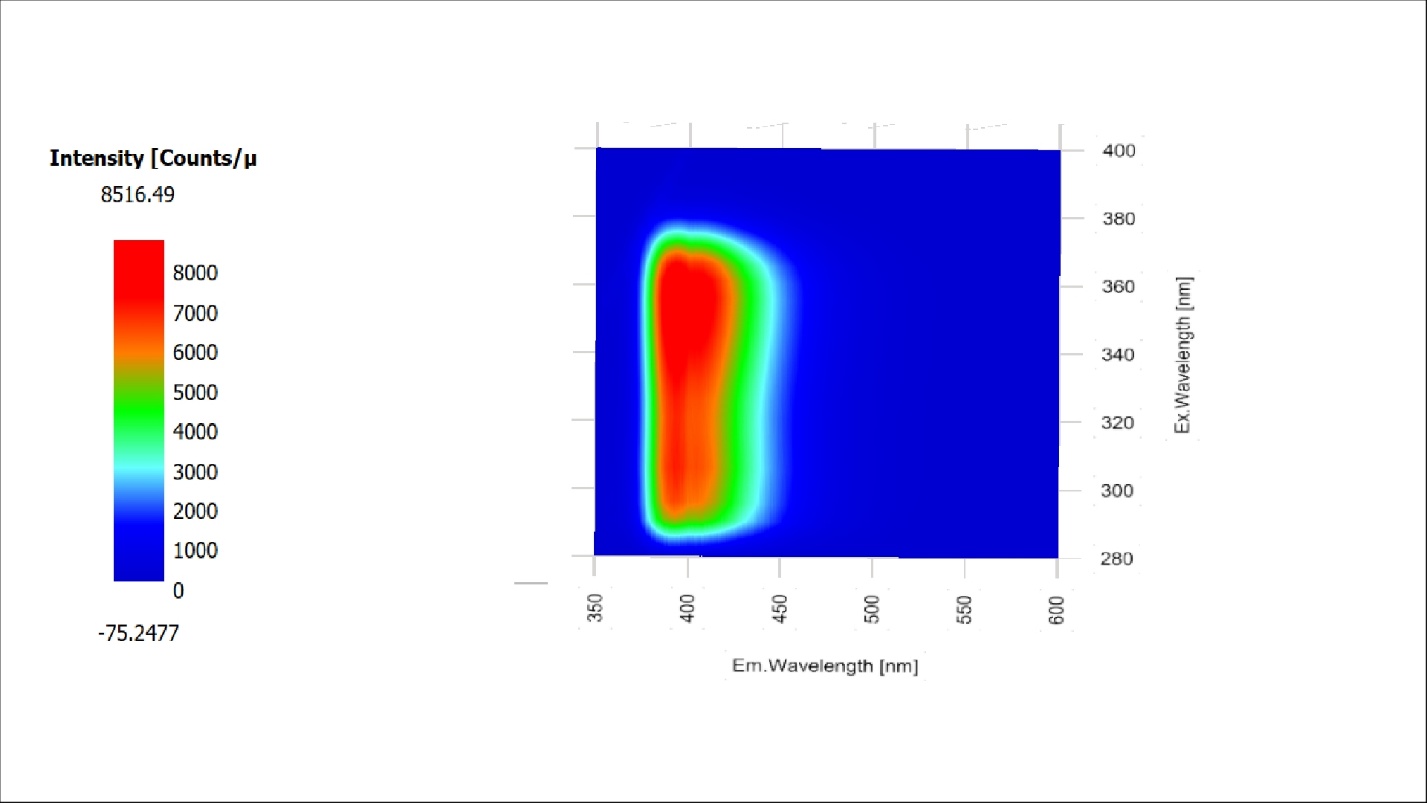


Figure S34: Excitation-emission map of C_12_OPh-BH_2_ (toluene).


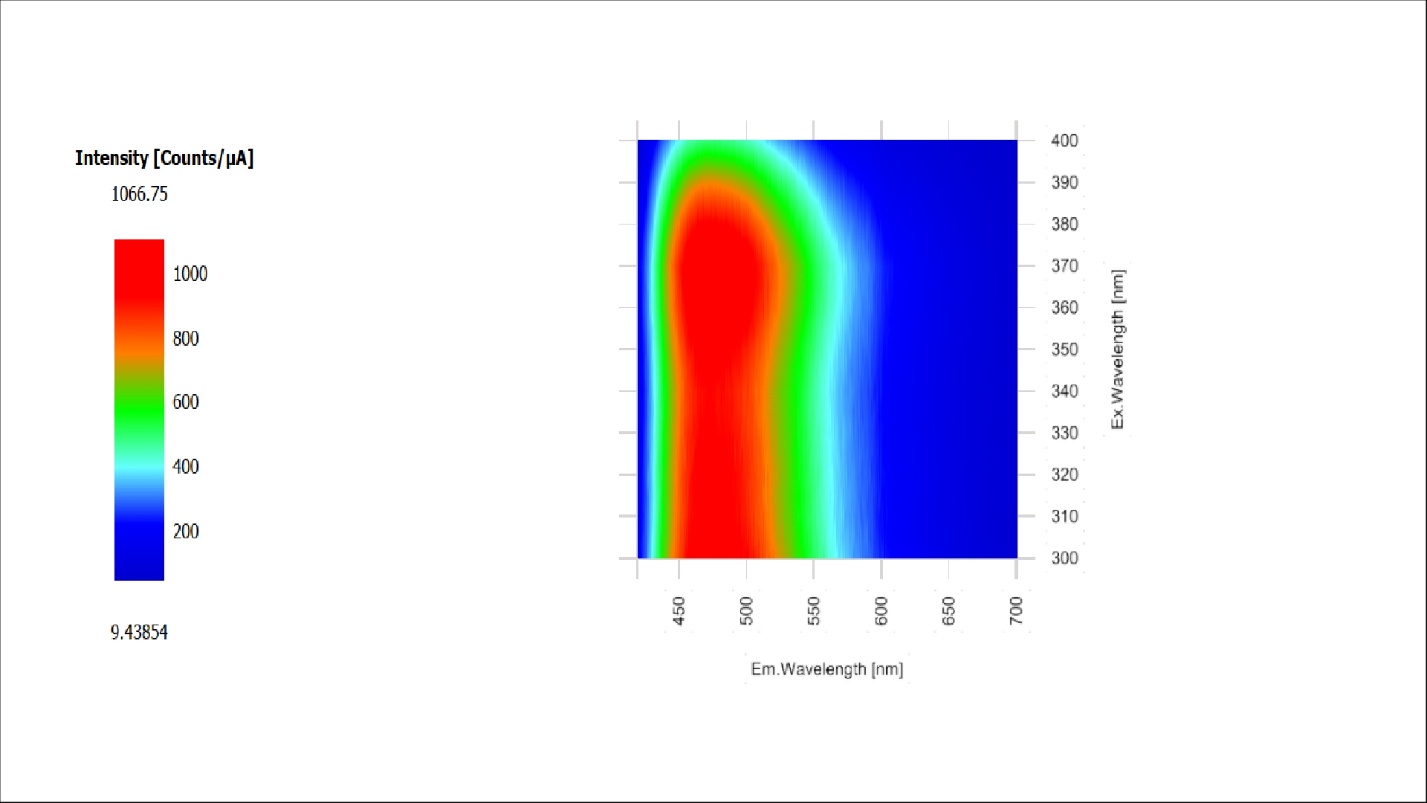


Figure S35: Excitation-emission map of C_12_OPh-BH_2_ (solid).


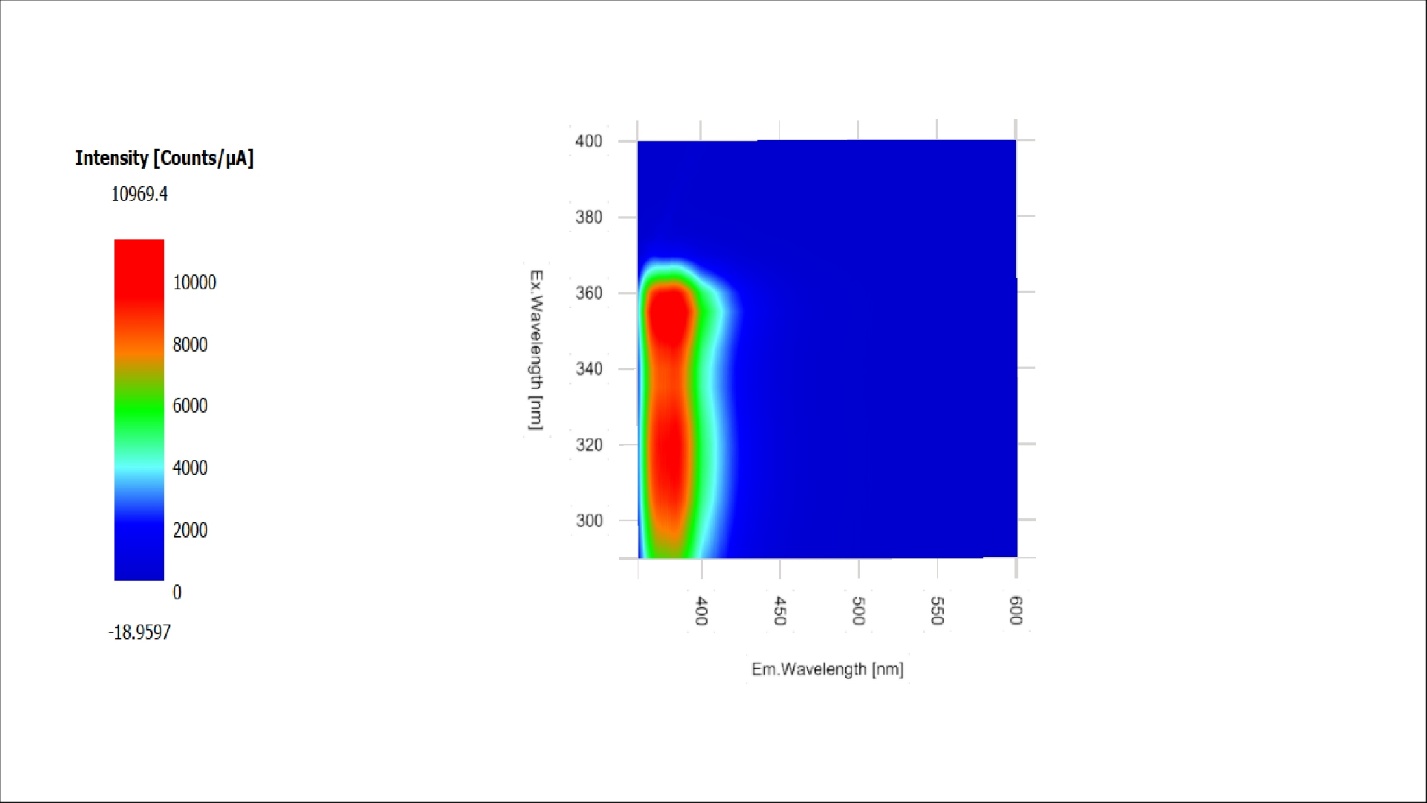


Figure S36: Excitation-emission map of BMe_2_-PhC_3_OCC_7_^F^ (toluene).


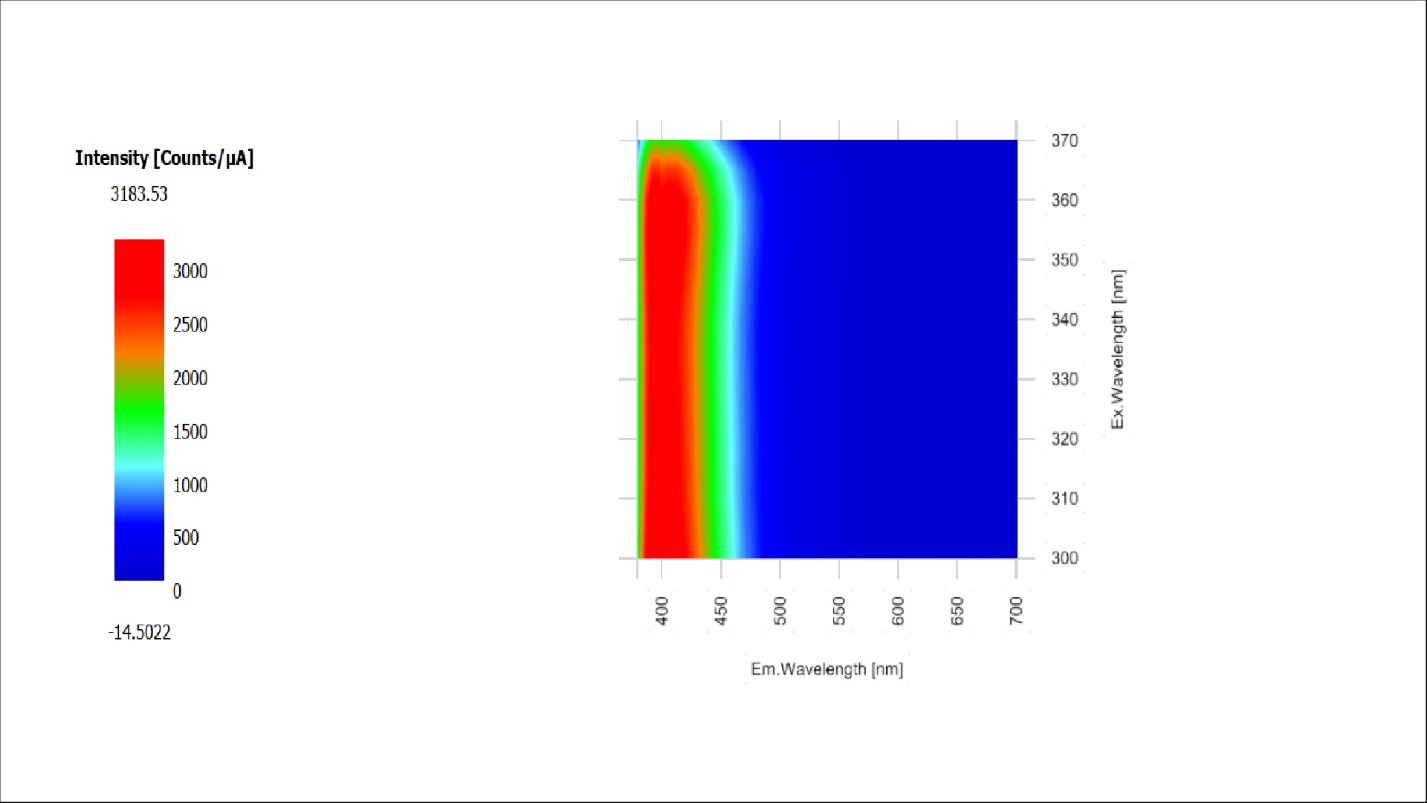


Figure S37: Excitation-emission map of BMe_2_-PhC_3_OCC_7_^F^ (solid).


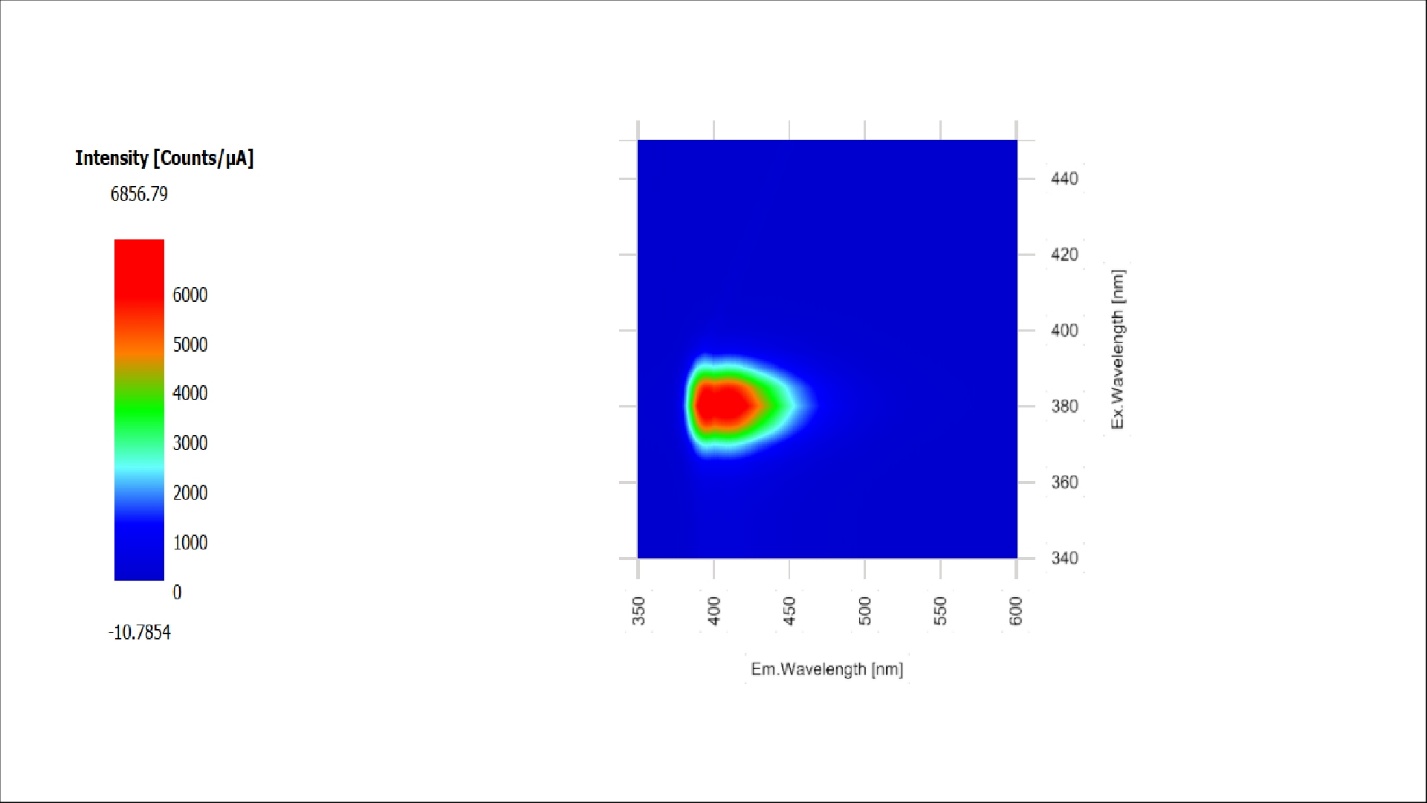


Figure S38: Excitation-emission map of BH_2_-Ph(OC_12_)_2_ (toluene).


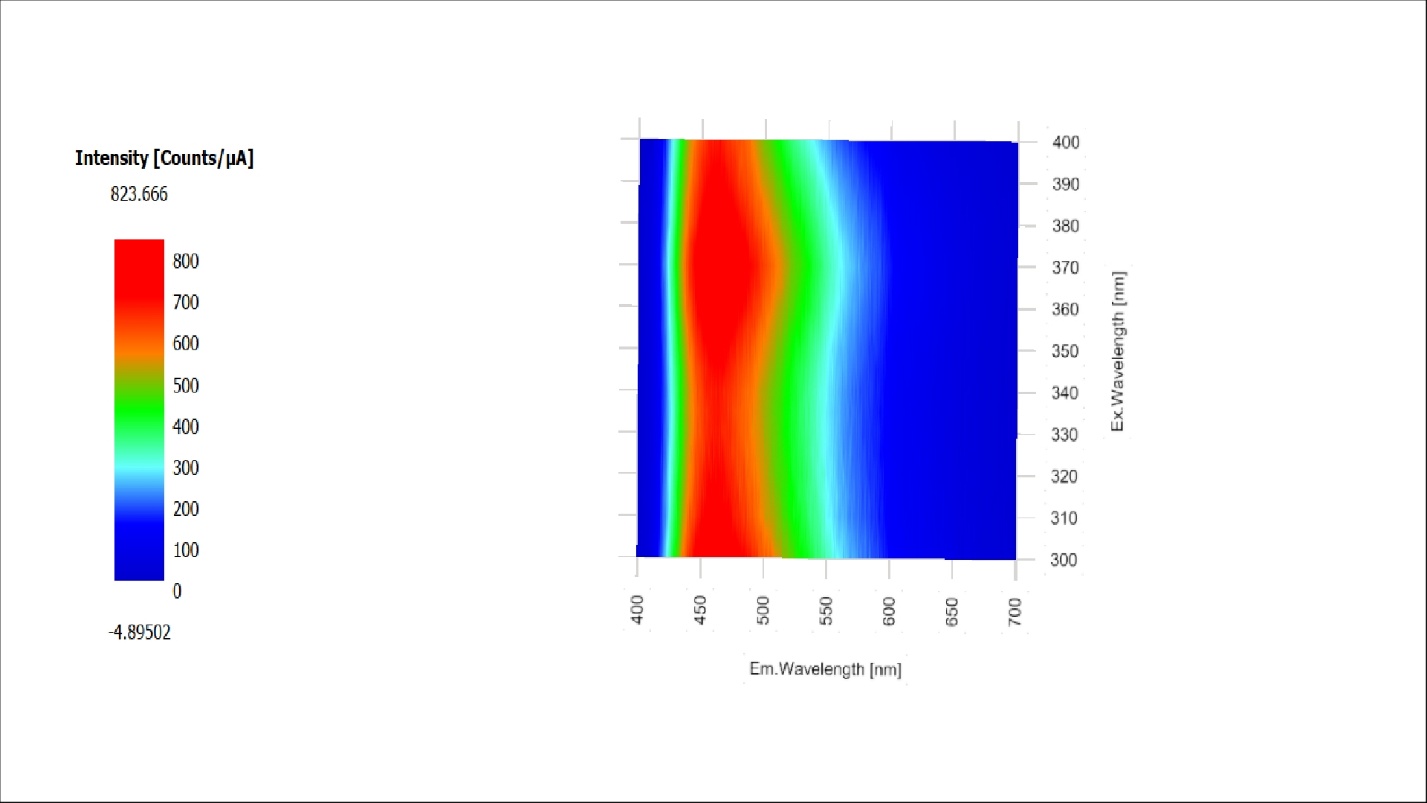


Figure S39: Excitation-emission map of BH_2_-Ph(OC_12_)_2_ (solid).


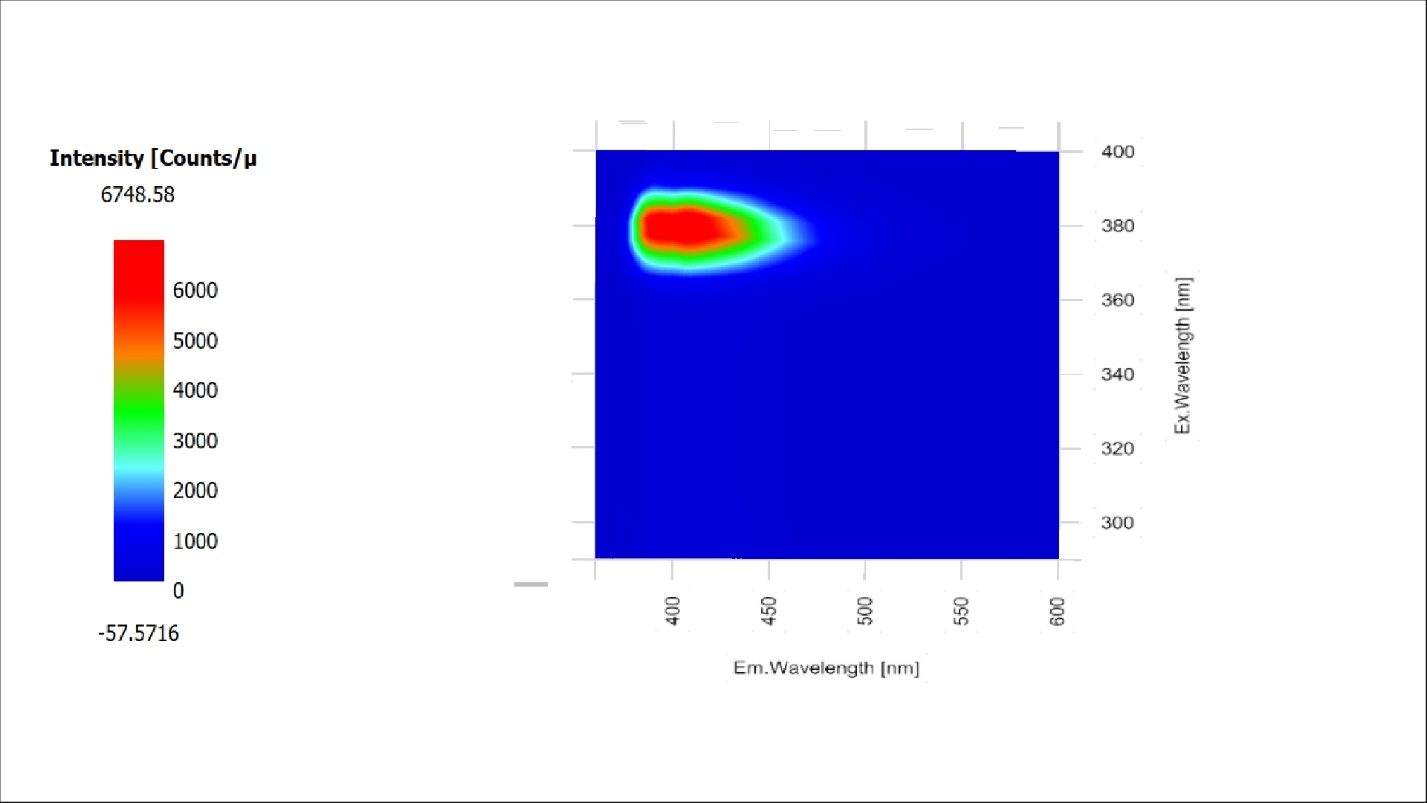


Figure S40: Excitation-emission map of BMe_2_-Ph(OC_12_)_3_ (toluene).


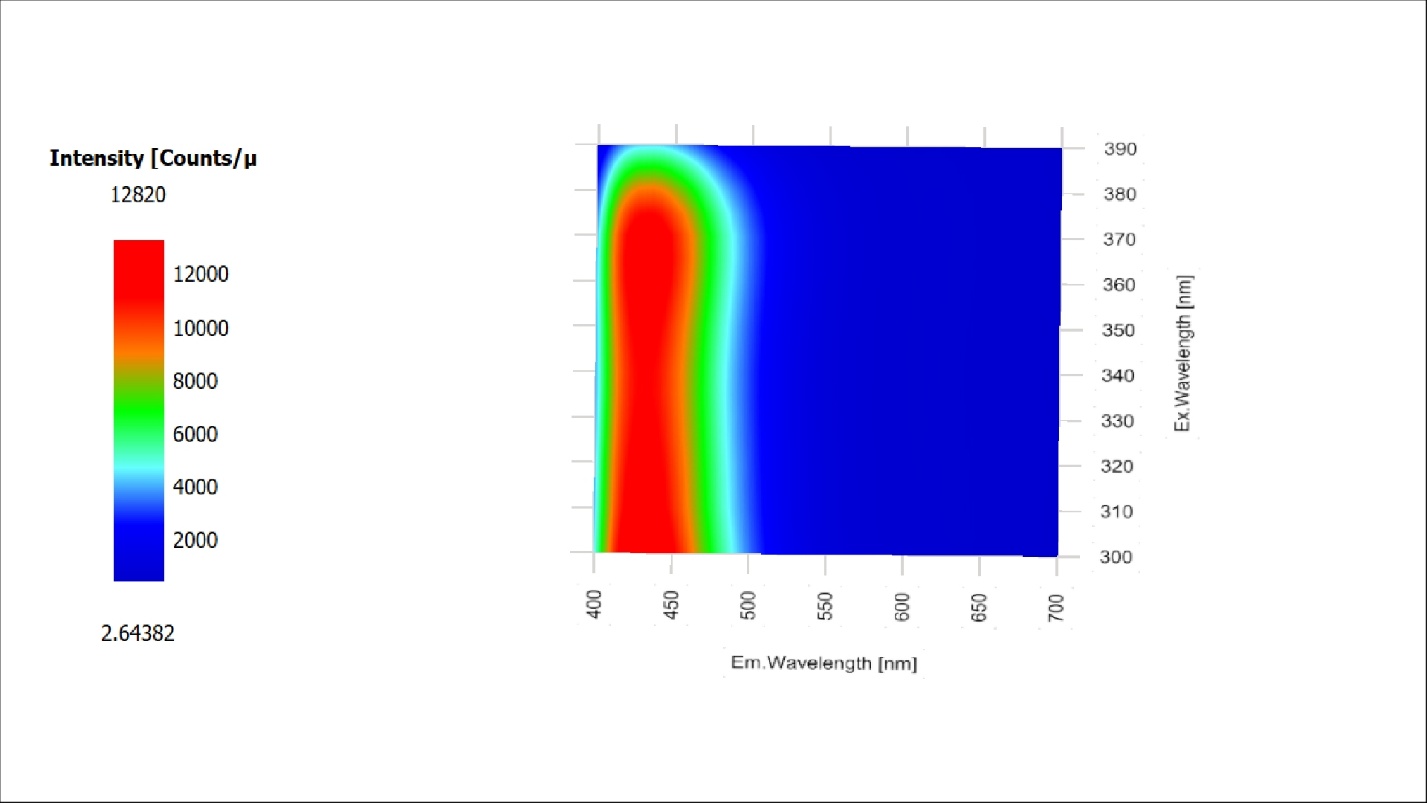


Figure S41: Excitation-emission map of BMe_2_-Ph(OC_12_)_3_ (solid).


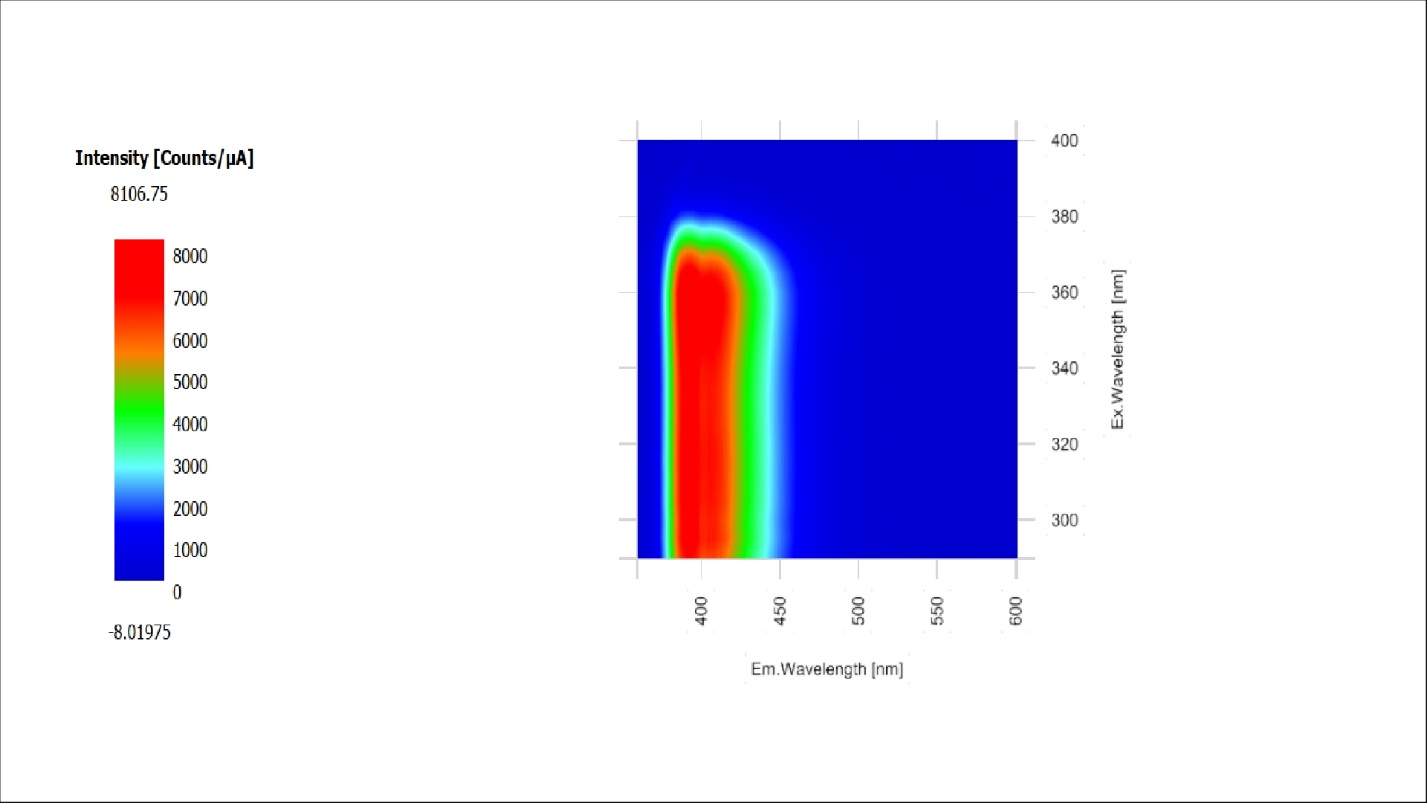


Figure S42: Excitation-emission map of BMe_2_-Ph(OC_12)2_ (toluene).


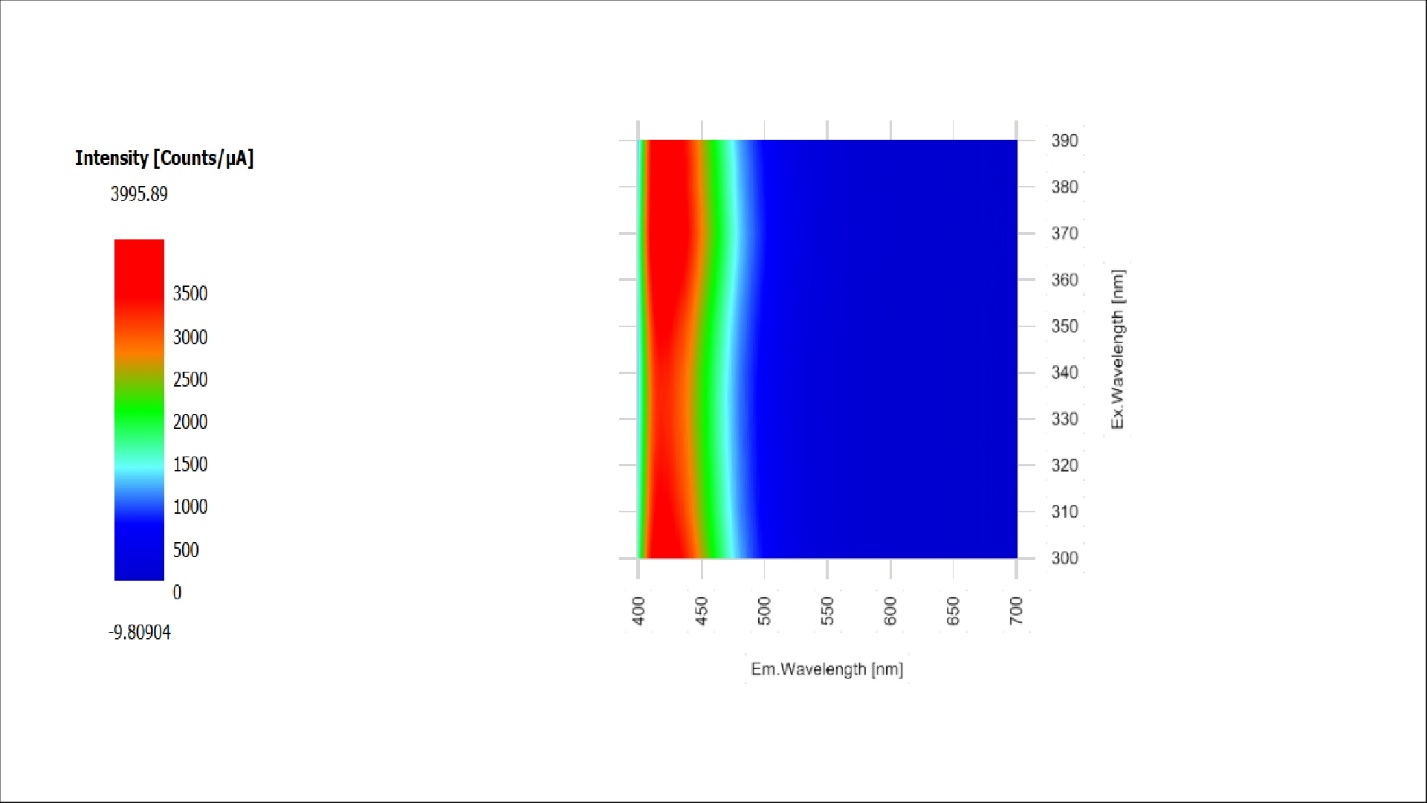


Figure S43: Excitation-emission map of BMe_2_-Ph(OC_12)2_ (solid).


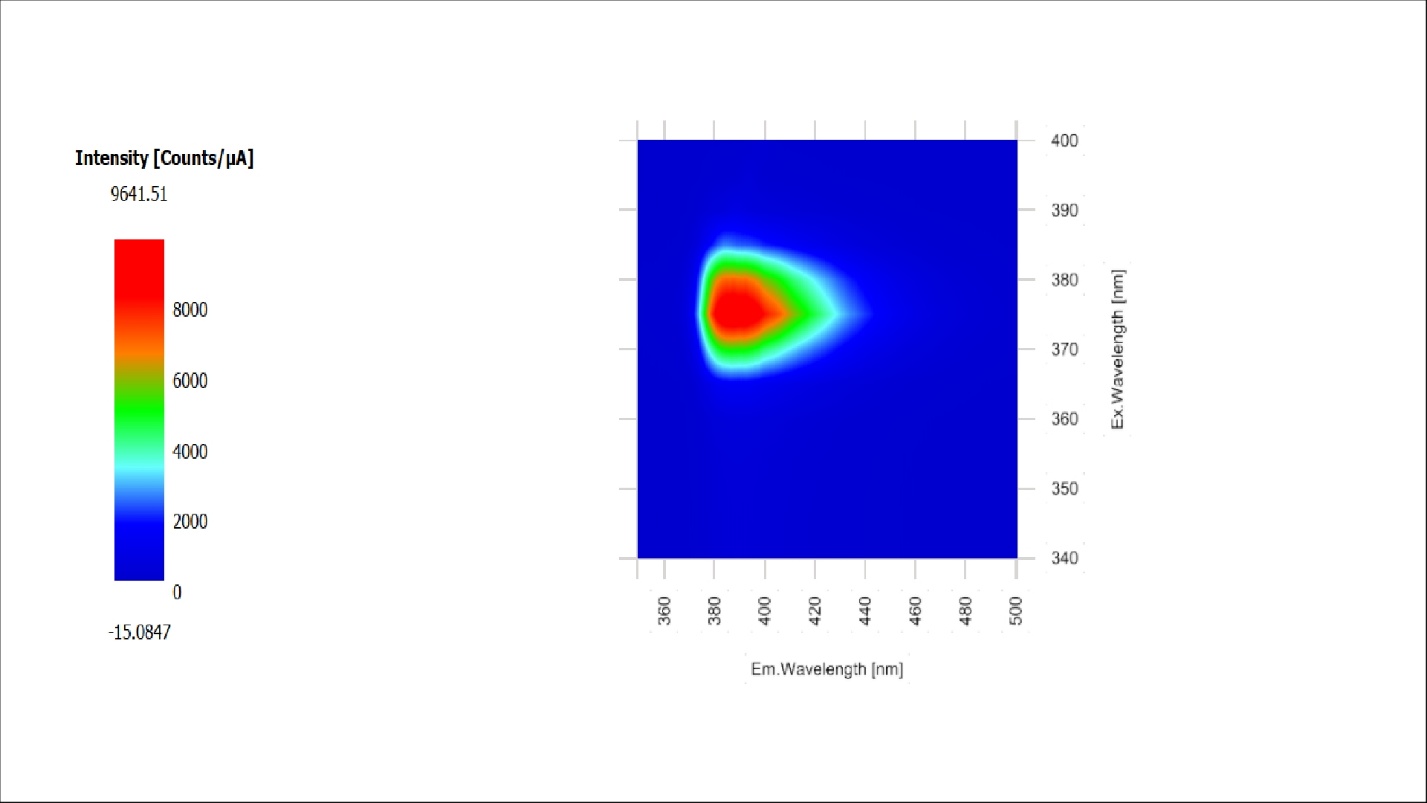


Figure S44: Excitation-emission map of BH_2_-PhOC_4_ (toluene).


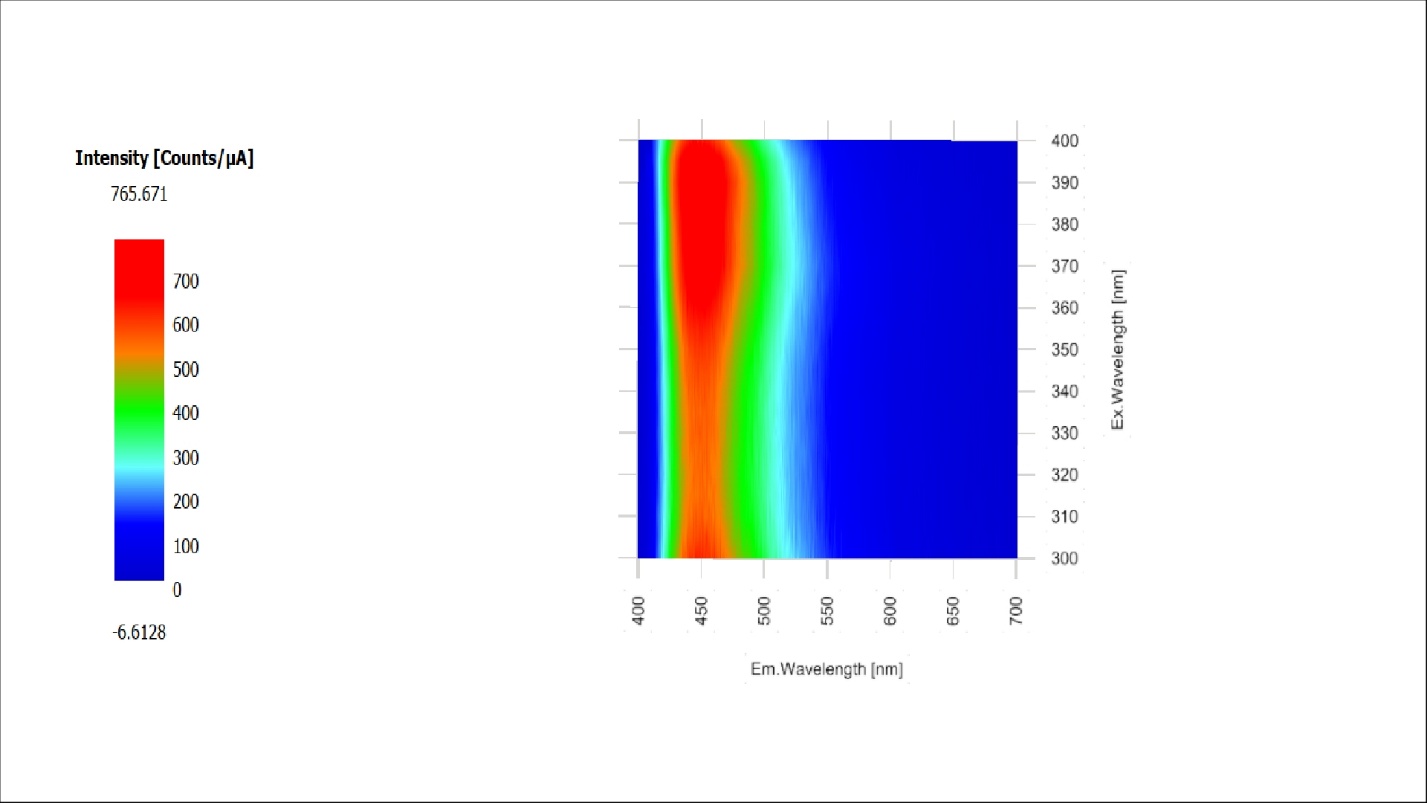


Figure S45: Excitation-emission map of BH_2_-PhOC_4_ (solid).


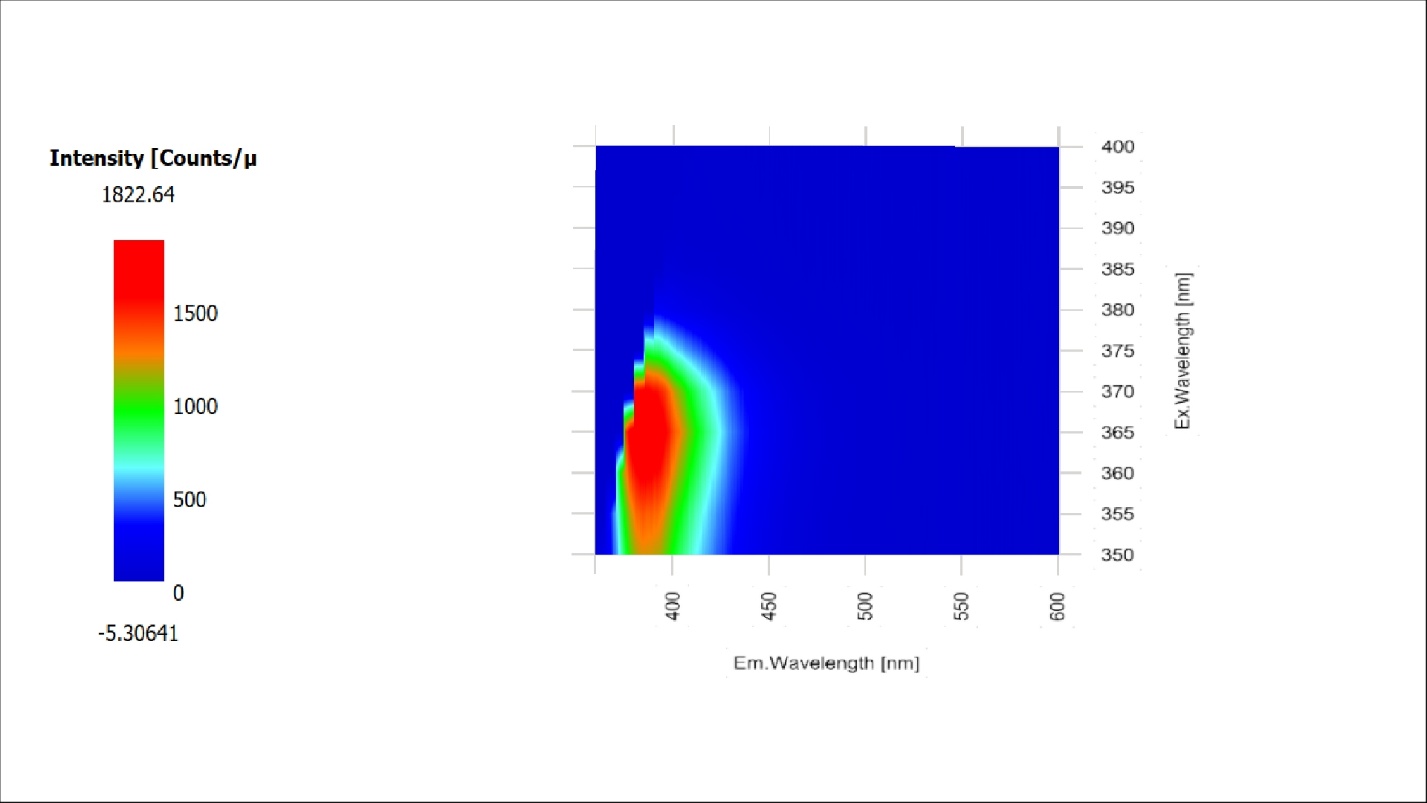


Figure S46: Excitation-emission map of BMe_2_-PhOC_12_ (toluene).


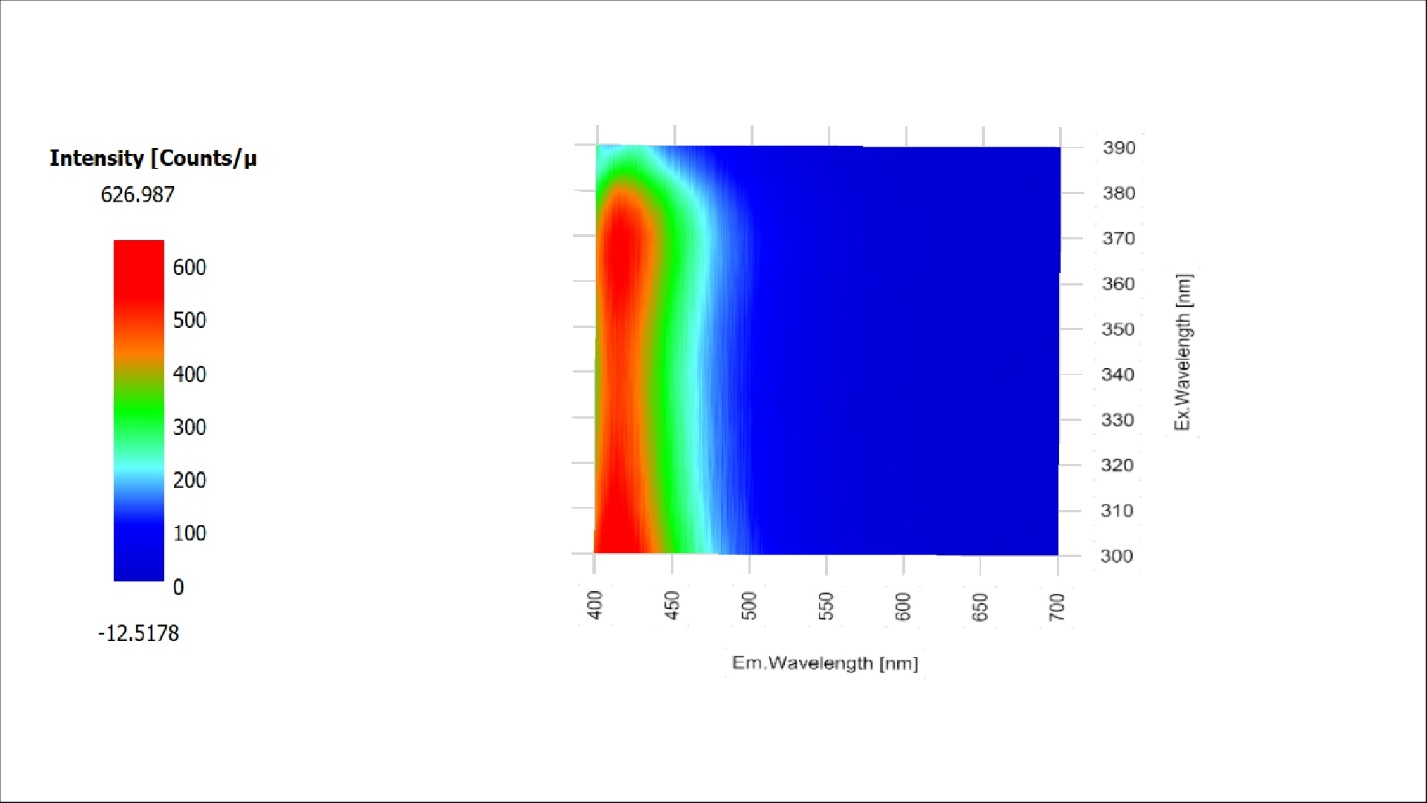


Figure S47: Excitation-emission map of BMe_2_-PhOC_12_ (solid).


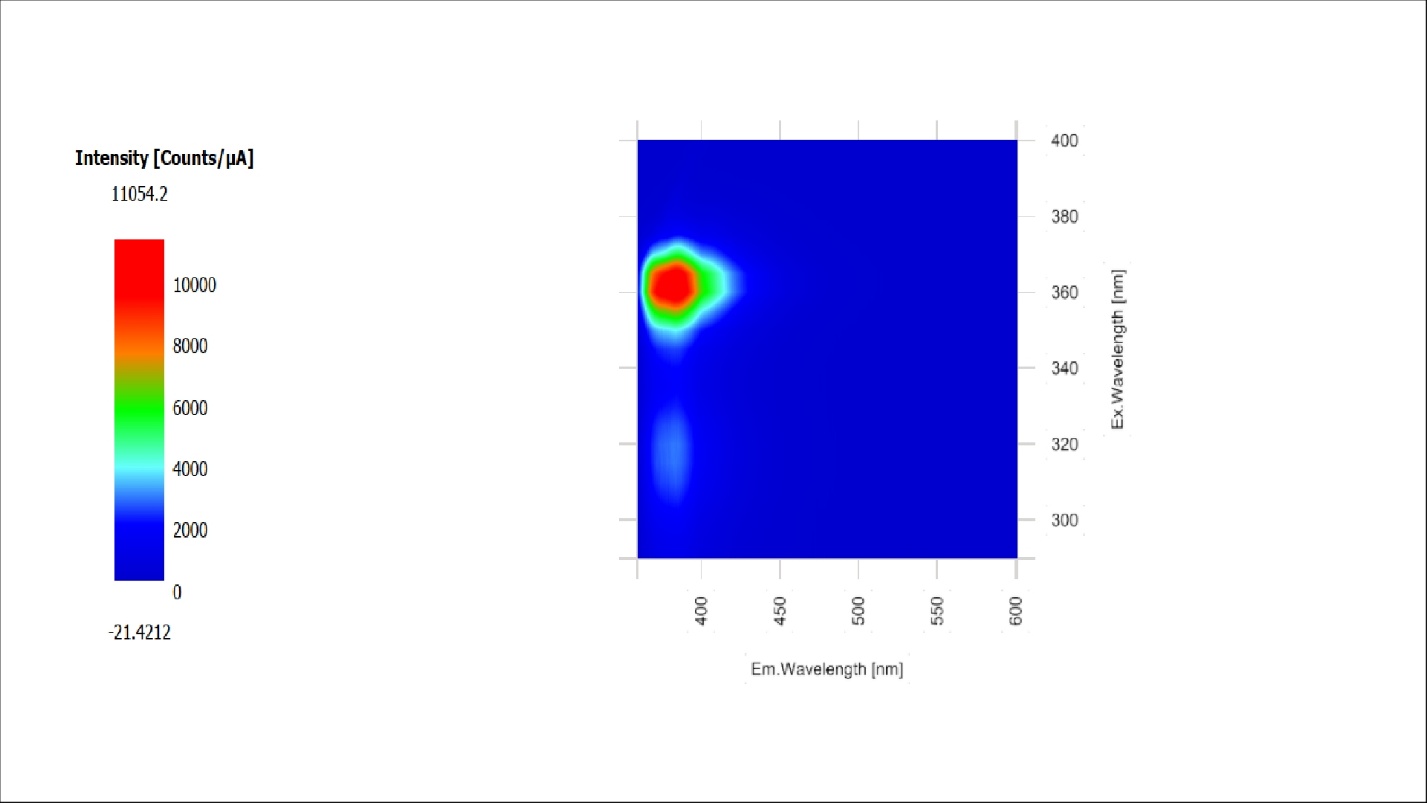


Figure S48: Excitation-emission map of BMe_2_-PhC_4_ (toluene).


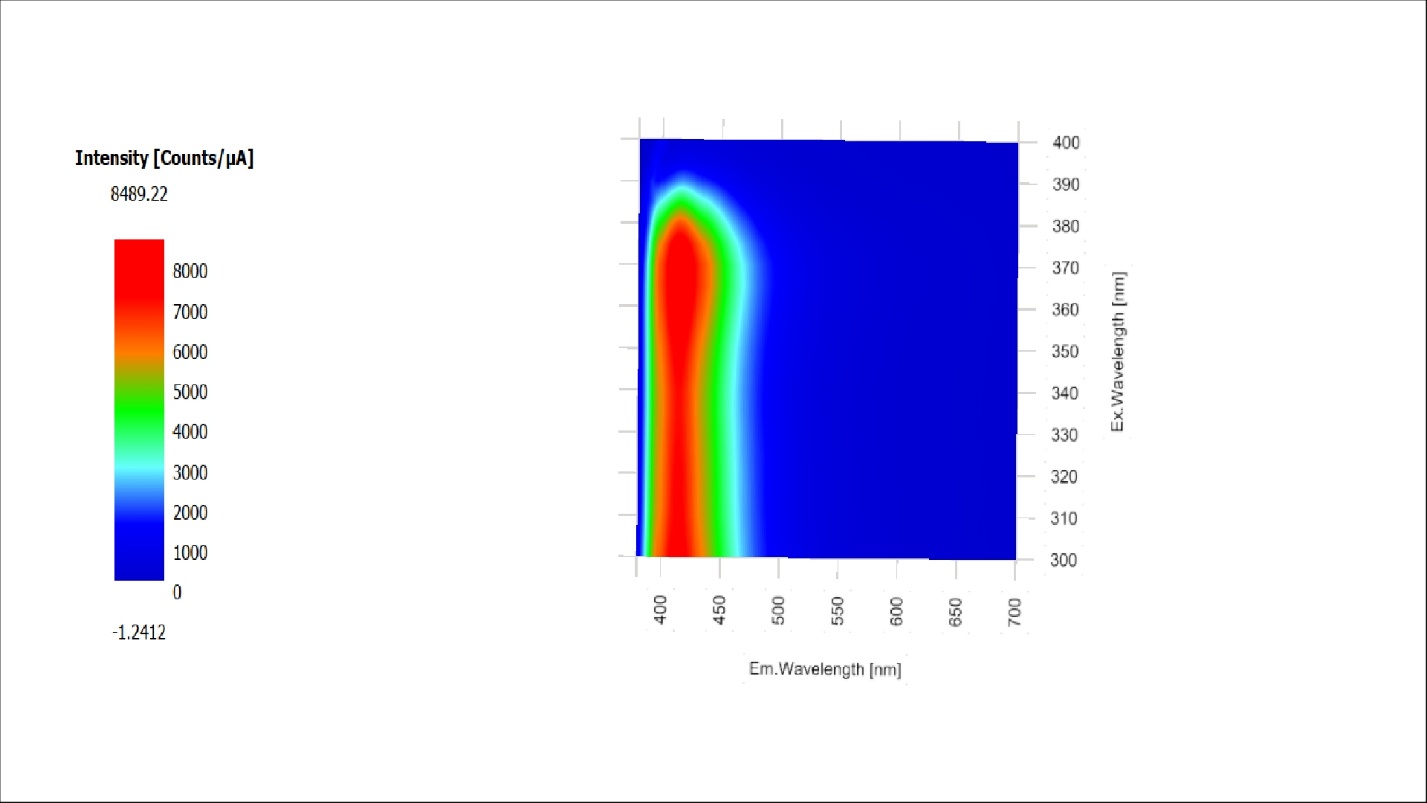


Figure S49: Excitation-emission map of BMe_2_-PhC_4_ (solid).


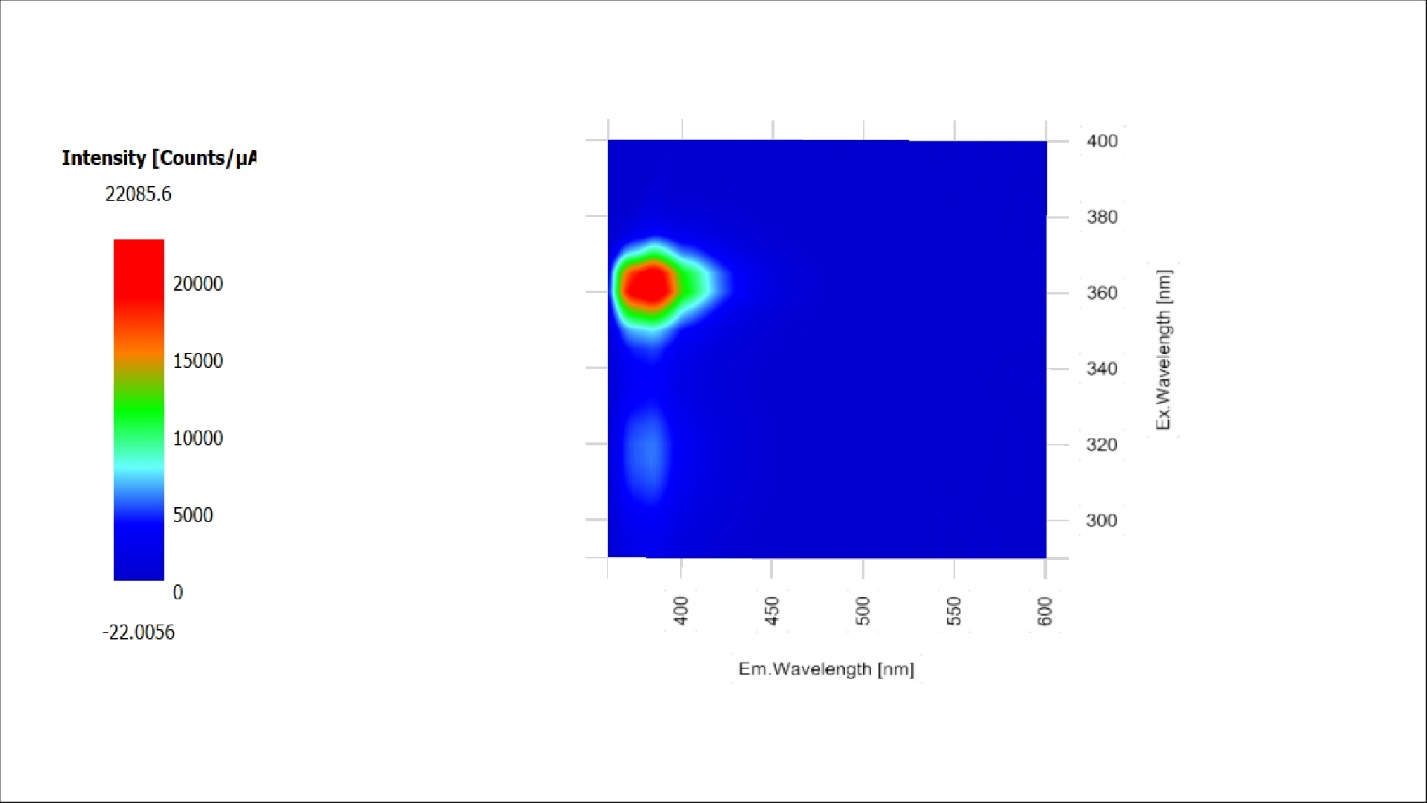


Figure S50: Excitation-emission map of BH_2_-PhC_12_ (toluene).


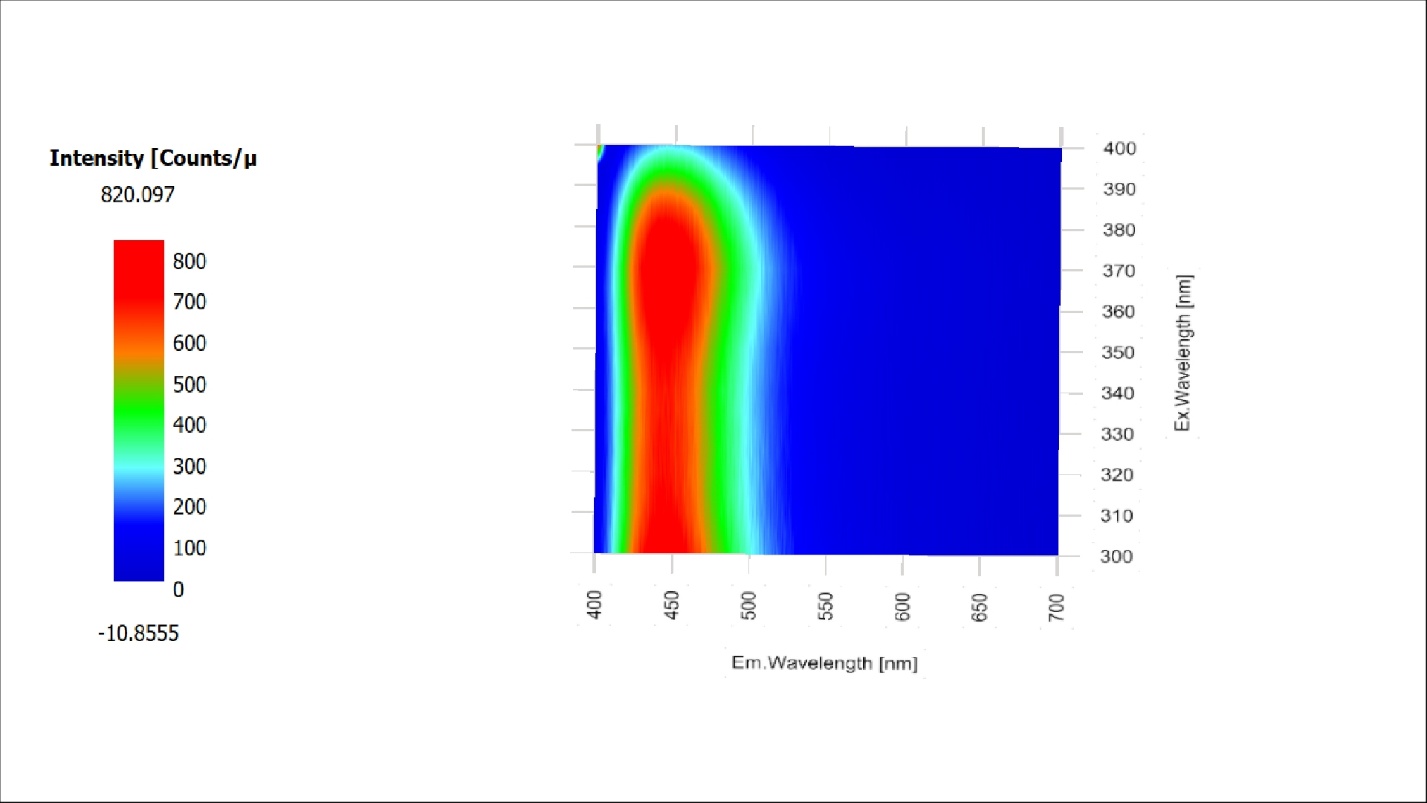


Figure S51: Excitation-emission map of BH_2_-PhC_12_ (solid).

- 1. Lifetime measurements


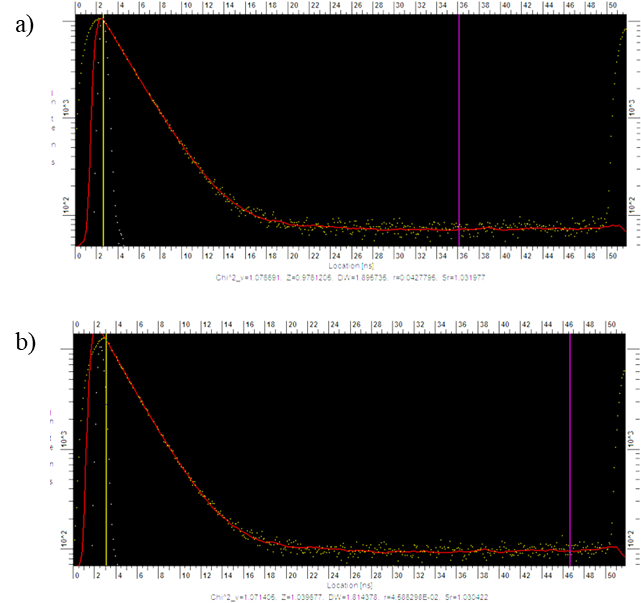


Figure S52: Integrated excited state emission decays (λ_exc_ = 375 nm) in toluene of a) BH_2_-PhC_4_, b) BH_2_-PhC_12_.


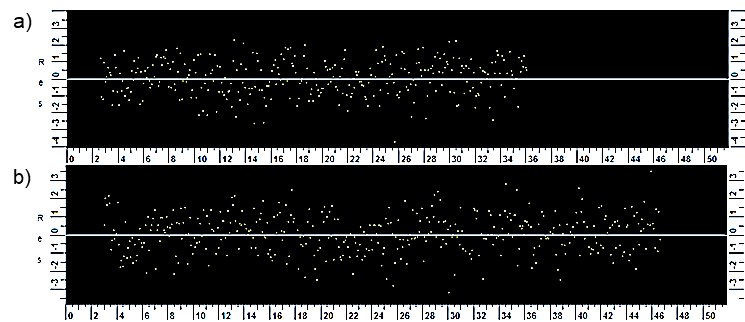


Figure S53: Residual distribution of the fit for Emission-lifetime measurements in toluene of a) BH_2_-PhC_4_, b) BH_2_-PhC_12_.


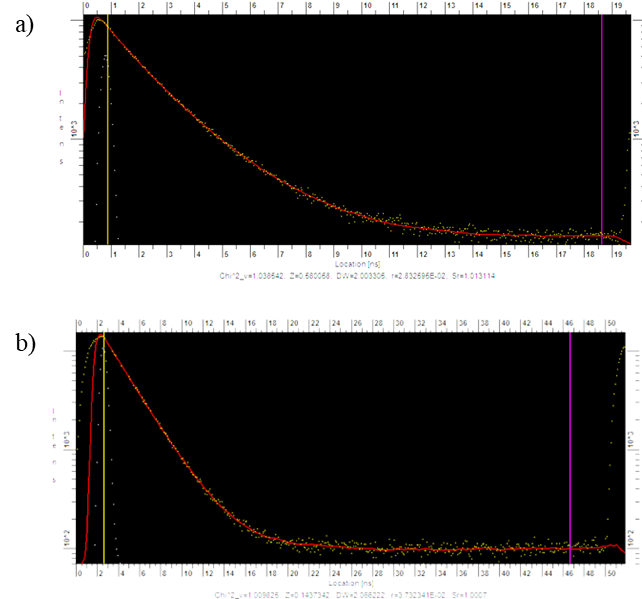


Figure S54: Emission-lifetime measurements in toluene of a) BMe_2_-PhC_4_, b) BH_2_-PhOC_12_.


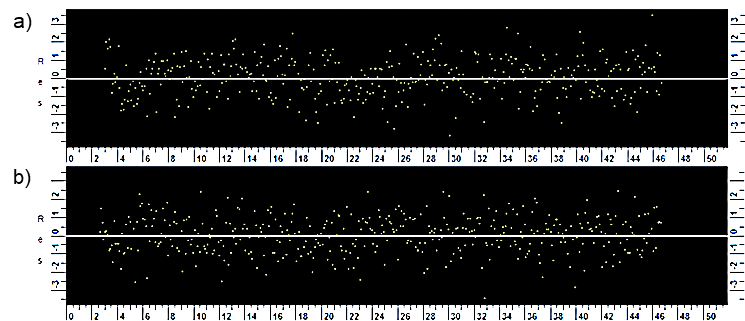


Figure S55: Residual distribution of the fit for Emission-lifetime measurements in toluene of a) BMe_2_-PhC_4_, b) BH_2_-PhOC_12_.


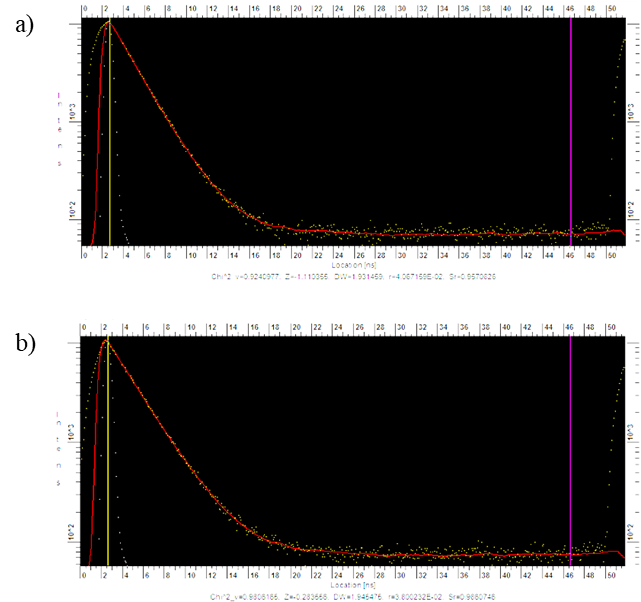


Figure S56: Emission-lifetime measurements in toluene of a) BMe_2_-PhOC_12_, b) BH_2_-PhOC_8_.


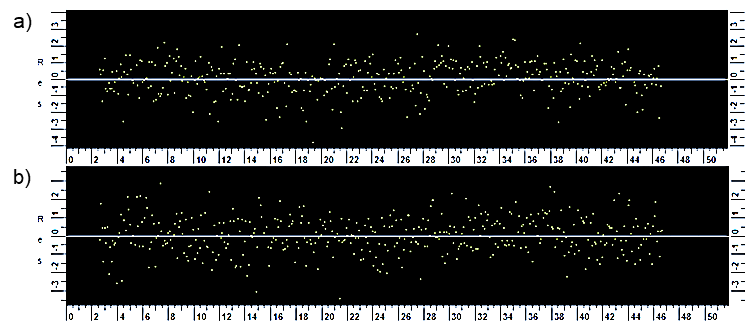


Figure S57: Residual distribution of the fit for Emission-lifetime measurements in toluene of a) BMe_2_-PhOC_12_, b) BH_2_-PhOC_8_.


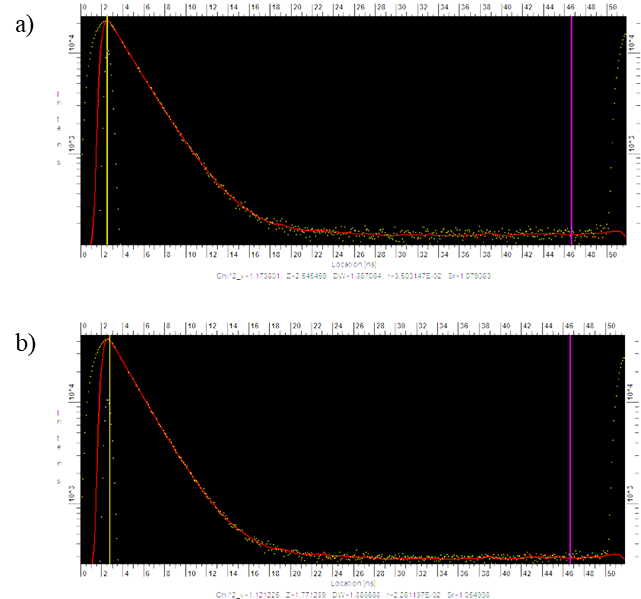


Figure S58: Emission-lifetime measurements in toluene of a) BH_2_-PhOC_4_, b) BMe_2_-Ph(OC_12)2_.


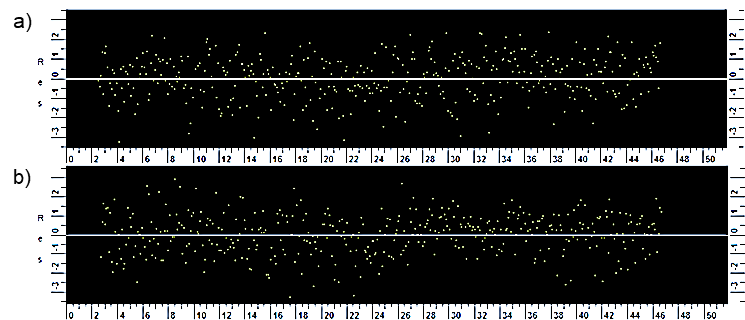


Figure S59: Residual distribution of the fit for Emission-lifetime measurements in toluene of a) BH_2_-PhOC_4_, b) BMe_2_-Ph(OC_12)2_.


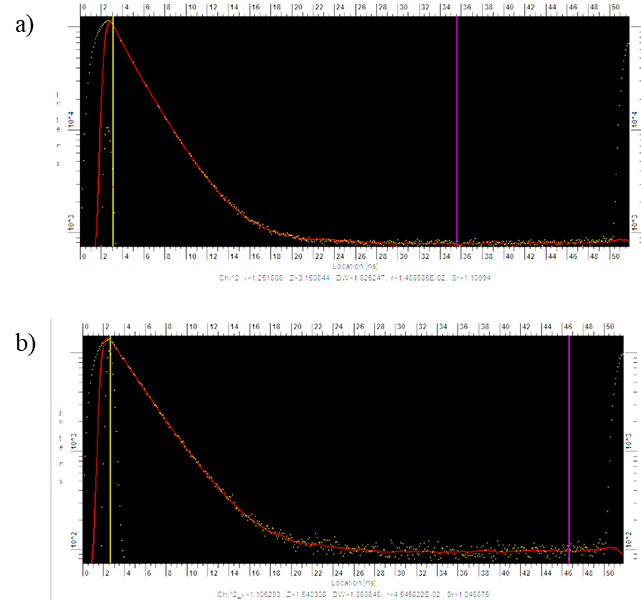


Figure S60: Emission-lifetime measurements in toluene of a) BMe_2_-Ph(OC_12_)_3_, b) BH_2_-Ph(OC_12_)_2_.


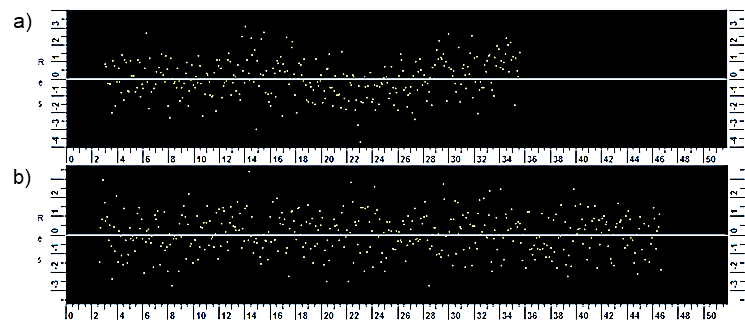


Figure S61: Residual distribution of the fit for Emission-lifetime measurements in toluene of a) BMe_2_-Ph(OC_12_)_3_, b) BH_2_-Ph(OC_12_)_2_.


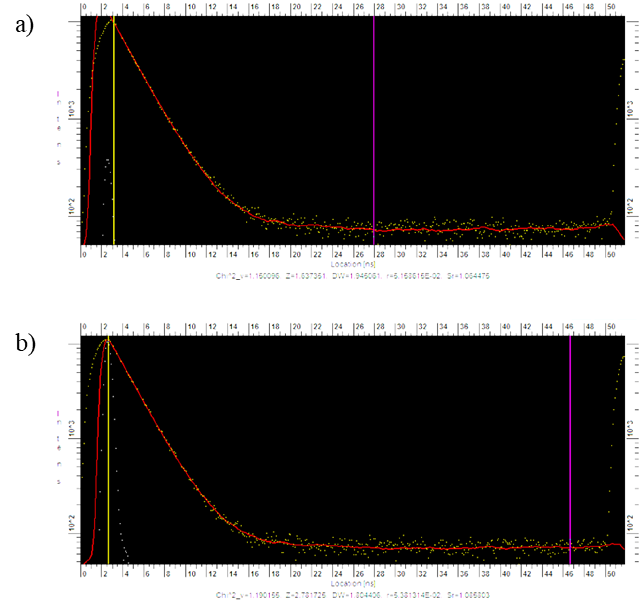


Figure S62: Emission-lifetime measurements in toluene of a) BMe_2_-PhC_3_OCC_7_^F^, b) C_12_OPh-BH_2_.


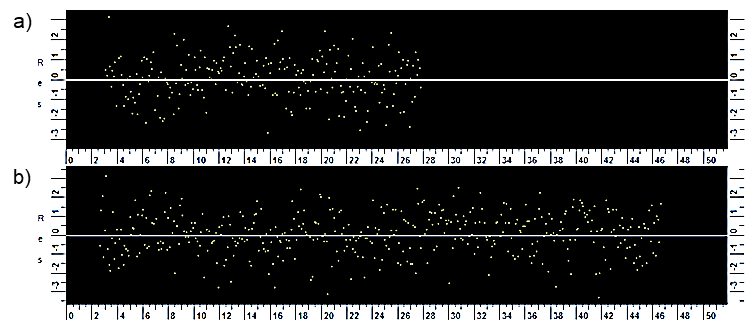


Figure S63: Residual distribution of the fit for Emission-lifetime measurements in toluene of a) BMe_2_-PhC_3_OCC_7_^F^, b) C_12_O-BH_2_.

- 1. Solvatochromism

Figure S64: Emission spectra of BH_2_-PhC_4_ in methylcyclohexane (MeCy), toluene, dichloromethane (CH_2_Cl_2_), tetrahydrofuran (THF) and butyronitrile (BuCN) (*c* = 0.02 mM, λ_exc_ = 300 nm).

Table S4: Emission maxima and FWHM of BH_2_-PhC_4_ in methylcyclohexane (MeCy=, toluene, dichloromethane (CH_2_Cl_2_), tetrahydrofuran (THF) and butyronitrile (BuCN) (*c* = 0.02 mM, λ_exc_ = 300 nm).

| solvent | λ_em_/ nm (FWHM / [nm] (cm^-1^)) |
| --- | --- |
| MeCy | 384 [47] (3096) |
| Toluene | 386 [45] (2992) |
| CH_2_Cl_2_ | 387 [46] (3021) |
| THF | 385 [46] (3074) |
| BuCN | 385 [43] (2880) |

- 1. Emission in the Mesophase

Figure S65: Emission spectra in the mesophase of a) BH_2_-PhC_12_ (90 °C, measured in cooling at the POM), b) BH_2_-PhOC_4_ (light blue: 90 °C, measured in cooling, dark blue: 120 °C, measured in cooling at the POM).

Figure S66: Emission spectra in the mesophase of a) BH_2_-Ph(OC_12_)_2_ (40 °C, measured in cooling at the POM), b) BMe_2_-PhC_3_OCC_7_^F^ (30 °C, measured in cooling at the POM).

Figure S67: Emission spectra in the mesophase of a) C_12_OPh-BH_2_ (100 °C, measured in cooling at the POM), b) BH_2_-PhOC_8_ (120 °C, measured in cooling at the POM).

Figure S68: Emission spectra in the mesophase of a) BH_2_-OC_12_ (110 °C, measured in cooling at the POM), b) BH_2_-PhC_4_ (60 °C, measured in cooling at the POM).

Table S5: Emission maxima and FWHM of the mesophase emission of boron *C,N*-chelates BH_2_-Ar, BMe_2_-Ar, Ar-BH_2_.

| compound | λ_em_ (mesophase) /nm  FWHM / [nm] (cm^-1^) | temperature / °C |
| --- | --- | --- |
| **BH_2_-PhC_4_** | 537 [111] (3799) | 60 |
| **BH_2_-PhC_12_** | 461 [60] (26059 | 90 |
| **BH_2_‑PhOC_4_** | 522 [93] (3668)  516 [82] (2763) | 90 (SmA)  120 (N) |
| **BH_2_‑PhOC_8_** | 488 [93] (3877) | 120 |
| **BH_2_‑PhOC_12_** | 514 [100] (3713) | 110 |
| **BH_2_‑Ph(OC_12_)_2_** | 476 [82] (3413) | 40 |
| **C_12_OPh-BH_2_** | 476 [93] (3785) | 100 |
| **BMe_2_-PhC_3_OCC_7_^F^** | 475 [90] (3876) | 30 |

Table S6: Detailed Photophysical properties (absorption maxima λ_max_ and emission maxima λ_em_ with FWHM_,_ stokes shifts Δ𝑣̅, quantum yields Φ, lifetimes τ, χ^2^ and *k* values) of boron *C,N*-chelates BH_2_-Ar, BMe_2_-Ar, Ar-BH_2_.

| compound | λ_max_ (toluene) /nm  (ε / mol^-1^ cm^-1^) | λ_em_ (toluene) /nm  FWHM [nm], (cm^-1^) | λ_em_ (solid) /nm FWHM [nm], (cm^-1^) | Δ𝑣̅ / cm^-^ | Φ_tol_ / % | Φ_solid_ / % | τ / ns | χ^2^ | *k*_r_ /  10^7^ s^-1^ | *k*_nr_ /  10^7^ s^-1^ |
| --- | --- | --- | --- | --- | --- | --- | --- | --- | --- | --- |
| **BH_2_-PhC_4_** | 342 (17000) | 385 [34] (2283) | 473 [69] (3485) | 3266 | 66 | 7 | 2.33 | 1.08 | 28.33 | 14.59 |
| **BH_2_-PhC_12_** | 338 (13000) | 385 [39] (2679) | 445 [68] (3377) | 3612 | 61 | 10 | 2.27 | 1.07 | 26.87 | 17.18 |
| **BH_2_‑PhOC_4_** | 346 (19000) | 386 [41] (2577) | 444 [77] (3636) | 2995 | 86 | 8 | 2.47 | 1.17 | 34.82 | 5.67 |
| **BH_2_‑PhOC_8_** | 346 (17000) | 391 [39] (2449) | 452[86] (4009) | 3326 | 69 | 10 | 2.53 | 0.98 | 27.38 | 12.30 |
| **BH_2_‑PhOC_12_** | 346 (18000) | 389 [41] (2577) | 447 [85] (4009) | 3195 | 79 | 14 | 2.46 | 1.01 | 32.11 | 8.54 |
| **BH_2_‑Ph(OC_12_)_2_** | 346 (16000) | 395 [56] (3293) | 462 [101] (4423) | 3585 | 75 | 9 | 2.73 | 1.11 | 27.47 | 9.16 |
| **C_12_OPh-BH_2_** | 346 (21000) | 393 [54] (3285) | 473 [107] (4498) | 3456 | 100 | 10 | 2.11 | 1.19 | 47.14 | 0.48 |
| **BMe_2_-PhC_4_**^[a]^ | 336 (18000) | 384 [38] (2555) | 414 [62] (3477) | 3720 | 77 | 29 | 1.79 | 1.04 | 43.02 | 12.85 |
| **BMe_2_‑PhOC_12_** | 342 (20000) | 385 [40] (2619) | 416 [64] (3634) | 3266 | 81 | 5 | 2.27 | 0.92 | 35.68 | 8.37 |
| **BMe_2_‑Ph(OC_12_)_2_** | 352 (19000) | 392 [53] (3241) | 417 [60] (3233) | 2899 | 81 | 50 | 2.36 | 1.12 | 34.32 | 8.05 |
| **BMe_2_‑Ph(OC_12_)_3_**^[a]^ | 342 (20000) | 410 [68] (3942) | 431 [71] (3698) | 4850 | 85 | 70 | 2.30 | 1.25 | 36.96 | 6.52 |
| **BMe_2_-PhC_3_OC_7_^F^** | 338 (10000) | 384 [39] (2693) | 394 [86] (3984) | 3544 | 80 | 30 | 2.18 | 1.15 | 36.70 | 9.17 |

[a] emission decays were fitted with two components. τ_av_ , calculated as τ_av_ = (a_1_τ_1_^2^+a_2_τ_2_^2^ )/(a_1_τ_1_+a_2_τ_2_) is given.

1. NMR Spectra


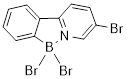


Figure S69: BBr_2_-Br ^1^H NMR.


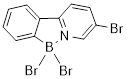


Figure S70: BBr_2_-Br ^11^B NMR.


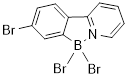


Figure S71: Br-BBr_2_ ^1^H NMR.


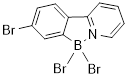


Figure S72: Br-BBr_2_ ^11^B NMR.


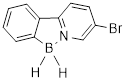


Figure S73: BH_2_-Br ^1^H NMR.


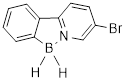


Figure S74: BH_2_-Br ^13^C NMR.


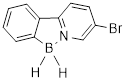


Figure S75: BH_2_-Br ^11^B NMR.


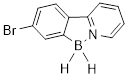


Figure S76: Br-BH_2_ ^1^H NMR.


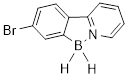


Figure S77: Br-BH_2_ ^13^C NMR.


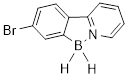


Figure S78: Br-BH_2_ ^11^B NMR.


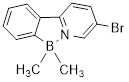


Figure S79: BMe_2_-Br ^1^H NMR.


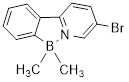


Figure S80: BMe_2_-Br ^13^C NMR.


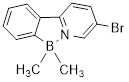


Figure S81: BMe_2_-Br ^11^B NMR.


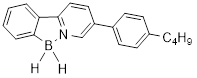


Figure S82: BH_2_-PhC_4_ ^1^H NMR.


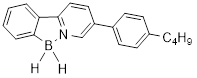


Figure S83: BH_2_-PhC_4_ ^13^C NMR.


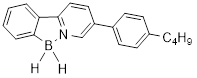


Figure S84: BH_2_-PhC_4_ ^11^B NMR.


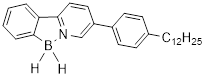


Figure S85: BH_2_-PhC_12_ ^1^H NMR.


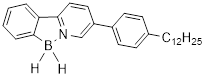


Figure S86 BH_2_-PhC_12_ ^13^C NMR.


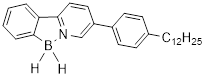


Figure S87: BH_2_-PhC_12_ ^11^B NMR.


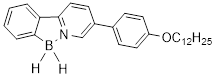


Figure S88: BH_2_-PhOC_12_ ^1^H NMR.


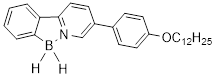


Figure S89: BH_2_-PhOC_12_ ^13^C NMR.


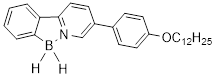


Figure S90: BH_2_-PhOC_12_ ^11^B NMR.


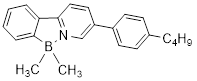


Figure S91: BMe_2_-PhC_4_ ^1^H NMR.


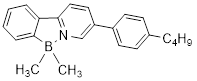


Figure S92: BMe_2_-PhC_4_ ^13^C NMR.


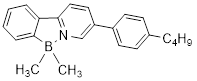


Figure S93: BMe_2_-PhC_4_ ^11^B NMR.


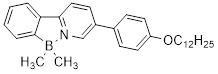


Figure S94: BMe_2_-PhOC_12_ ^1^H NMR.


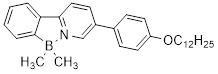


Figure S95: BMe_2_-PhOC_12_ ^13^C NMR.


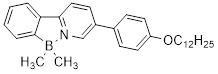


Figure S96: BMe_2_-PhOC_12_ ^11^B NMR.


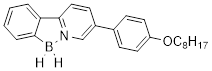


Figure S97: BMe_2_-PhOC_12_ ^1^H NMR.


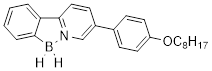


Figure S98: BH_2_-PhOC_8_ ^13^C NMR


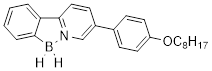


Figure S99: BH_2_-PhOC_8_ ^11^B NMR.


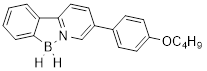


Figure S100: BH_2_-PhOC_4_ ^1^H NMR.


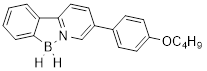


Figure S101: BH_2_-PhOC_4_ ^13^C NMR.


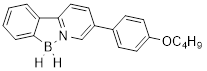


Figure S102: BH_2_-PhOC_4_ ^11^B NMR.


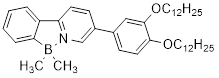


Figure S103: BMe_2_-Ph(OC_12)2_ ^1^H NMR.


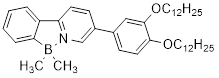


Figure S104: BMe_2_-Ph(OC_12)2_ ^13^C NMR.


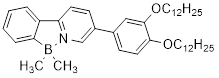


Figure S105: BMe_2_-Ph(OC_12)2_ ^11^B NMR.


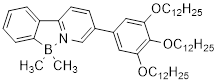


Figure S106: BMe_2_-Ph(OC_12)3_ ^1^H NMR.


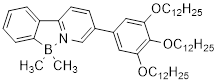


Figure S107: BMe_2_-Ph(OC_12_)_3_ ^13^C NMR.


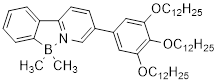


Figure S108: BMe_2_-Ph(OC_12_)_3_ ^11^B NMR.


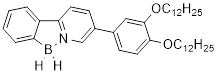


Figure S109: BH_2_-Ph(OC_12_)_2_ ^1^H NMR.


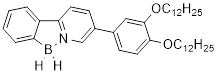


Figure S110: BH_2_-Ph(OC_12_)_2_ ^13^C NMR.


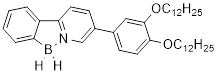


Figure S111: BH_2_-Ph(OC_12_)_2_ ^11^B NMR.


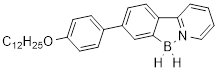


Figure S112: C_12_OPh-BH_2_ ^1^H NMR.


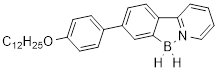


Figure S113: C_12_OPh-BH_2_ ^13^C NMR.


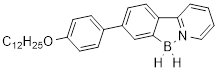


Figure S114: C_12_OPh-BH_2_ ^11^B NMR


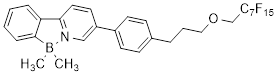


Figure S115: BMe_2_-PhC_3_OCC_7_^F^ ^1^H NMR.

Figure S116: BMe_2_-PhC_3_OCC_7_^F^ ^13^C NMR.


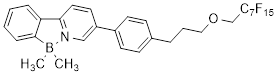


Figure S117: BMe_2_-PhC_3_OCC_7_^F^ ^11^B NMR.

**Figure S113:** **15** ^1^H NMR.

**Figure S114:** **15** ^13^C NMR.

**Figure S115:** **15** ^19^F NMR.

**Figure S116:** **BPin-8** ^1^H NMR.

**Figure S117:** **BPin-8** ^13^C NMR.

**Figure S118:** **BPin-8** ^11^B NMR.

**Figure S119:** **BPin-7** ^19^F NMR.

1. References

[49] G. R. Fulmer, A. J. M. Miller, N. H. Sherden, H. E. Gottlieb, A. Nudelman, B. M. Stoltz, J. E. Bercaw, K. I. Goldberg, “NMR Chemical Shifts of Trace Impurities: Common Laboratory Solvents, Organics, and Gases in Deuterated Solvents Relevant to the Organometallic Chemist” *Organometallics* **2010**, *29*, 2176–2179.

[50] T. K. Ellis, M. Galerne, J. J. Armao IV, A. Osypenko, D. Martel, M. Maaloum, G. Fuks, O. Gavat, E. Moulin, N. Giuseppone, “Supramolecular Electropolymerization” *Angewandte Chemie International Edition* **2018**, *57*, 15749–15753.

[51] C.-J. Zhao, D. Xue, Z.-H. Jia, C. Wang, J. Xiao, “Methanol-Promoted Borylation of Arylamines: A Simple and Green Synthetic Method to Arylboronic Acids and Arylboronates” *Synlett* **2014**, *25*, 1577–1584.

[52] M. Kaller, S. Tussetschläger, P. Fischer, C. Deck, A. Baro, F. Giesselmann, S. Laschat, “Columnar Mesophases Controlled by Counterions in Potassium Complexes of Dibenzo[18]crown-6 Derivatives” *Chemistry – A European Journal* **2009**, *15*, 9530–9542.

[53] T. Wöhrle, R. Gündemir, W. Frey, F. Knecht, A. Köhn, S. Laschat, “Thermotropic MIDA Boronates as a Case Study for the Role of Dipolar Interactions in Liquid Crystalline Self-Assembly” *Chemistry – A European Journal* **2017**, *23*, 4149–4159.

[54] T. Yasuda, T. Shimizu, F. Liu, G. Ungar, T. Kato, “Electro-Functional Octupolar π-Conjugated Columnar Liquid Crystals” *J. Am. Chem. Soc.* **2011**, *133*, 13437–13444.

[55] H. Maeda, Y. Haketa, T. Nakanishi, “Aryl-Substituted C3-Bridged Oligopyrroles as Anion Receptors for Formation of Supramolecular Organogels” *J. Am. Chem. Soc.* **2007**, *129*, 13661–13674.

[56] J. A. Knöller, F. Müller, T. Matulaitis, J. M. Dos Santos, A. K. Gupta, E. Zysman-Colman, S. Laschat, “MR-TADF liquid crystals: towards self assembling host–guest mixtures showing narrowband emission from the mesophase” *Chem. Sci.* **2024**, *15*, 18022–18030.

[57] D. V. Francis, D. H. Miles, A. I. Mohammed, R. W. Read, X. Wang, “Towards functional fluorous surfactants. Synthesis of hydrophilic fluorous 1,2,3-triazolylmethyl ethers and di(1,2,3-triazolylmethyl) ethers” *Journal of Fluorine Chemistry* **2011**, *132*, 898–906.

[58] S. Song, P. Zhang, H. Liu, X. Zhu, X. Feng, Z. Zhao, B. Z. Tang, “Conformation-dependent mechanochromic delayed fluorescence of AIE-active tetra-coordinated B–N complexes” *Dyes and Pigments* **2021**, *196*, 109776.

[59] Z. Feng, Y. Yu, X. Yang, Y. Sun, D. Zhong, X. Deng, G. Zhou, Z. Wu, “Manipulating MLCT transition character with ppy-type four-coordinate organoboron skeleton for highly efficient long-wavelength Ir-based phosphors in organic light-emitting diodes” *J. Mater. Chem. C* **2021**, *9*, 12650–12660.

[60] Y. Sun, Z. Wang, H. Xu, W. Ma, C.-L. Sun, J. Wu, X. Pan, “Highly Sensitive Solid Ratiometric Luminescent Thermometer Based on N,C-Chelating Four-Coordinate Organoboron Compounds” *Inorg. Chem.* **2024**, *63*, 22688–22698.

[61] P. Pracht, F. Bohle, S. Grimme, “Automated exploration of the low-energy chemical space with fast quantum chemical methods” *Phys. Chem. Chem. Phys.* **2020**, *22*, 7169–7192.

[62] S. Grimme, “Exploration of Chemical Compound, Conformer, and Reaction Space with Meta-Dynamics Simulations Based on Tight-Binding Quantum Chemical Calculations” *J. Chem. Theory Comput.* **2019**, *15*, 2847–2862.

[63] C. Bannwarth, S. Ehlert, S. Grimme, “GFN2-xTB—An Accurate and Broadly Parametrized Self-Consistent Tight-Binding Quantum Chemical Method with Multipole Electrostatics and Density-Dependent Dispersion Contributions” *J. Chem. Theory Comput.* **2019**, *15*, 1652–1671.

[64] S. G. Balasubramani, G. P. Chen, S. Coriani, M. Diedenhofen, M. S. Frank, Y. J. Franzke, F. Furche, R. Grotjahn, M. E. Harding, C. Hättig, A. Hellweg, B. Helmich-Paris, C. Holzer, U. Huniar, M. Kaupp, A. Marefat Khah, S. Karbalaei Khani, T. Müller, F. Mack, B. D. Nguyen, S. M. Parker, E. Perlt, D. Rappoport, K. Reiter, S. Roy, M. Rückert, G. Schmitz, M. Sierka, E. Tapavicza, D. P. Tew, C. van Wüllen, V. K. Voora, F. Weigend, A. Wodyński, J. M. Yu, “TURBOMOLE: Modular program suite for ab initio quantum-chemical and condensed-matter simulations” *The journal of chemical physics* **2020**, *152*, 184107.

[65] Y. J. Franzke, C. Holzer, J. H. Andersen, T. Begušić, F. Bruder, S. Coriani, F. Della Sala, E. Fabiano, D. A. Fedotov, S. Fürst, S. Gillhuber, R. Grotjahn, M. Kaupp, M. Kehry, M. Krstić, F. Mack, S. Majumdar, B. D. Nguyen, S. M. Parker, F. Pauly, A. Pausch, E. Perlt, G. S. Phun, A. Rajabi, D. Rappoport, B. Samal, T. Schrader, M. Sharma, E. Tapavicza, R. S. Treß, V. Voora, A. Wodyński, J. M. Yu, B. Zerulla, F. Furche, C. Hättig, M. Sierka, D. P. Tew, F. Weigend, “TURBOMOLE: Today and Tomorrow” *J. Chem. Theory Comput.* **2023**, *19*, 6859–6890.

[66] Y. Zhao, D. G. Truhlar, “The M06 suite of density functionals for main group thermochemistry, thermochemical kinetics, noncovalent interactions, excited states, and transition elements: two new functionals and systematic testing of four M06-class functionals and 12 other functionals” *Theor Chem Account* **2008**, *120*, 215–241.

[67] R. Al-Saadon, T. Shiozaki, G. Knizia, “Visualizing Complex-Valued Molecular Orbitals” *J. Phys. Chem. A* **2019**, *123*, 3223–3228.
